# Correction: A novel nematode species from the Siberian permafrost shares adaptive mechanisms for cryptobiotic survival with *C*. *elegans* dauer larva

**DOI:** 10.1371/journal.pgen.1010943

**Published:** 2023-09-14

**Authors:** The PLOS Genetics Staff

## Notice of republication

This article was republished on August 31, 2023, to include previously ommitted details concerning taxonomic registration. Please download this article again to view the correct version.
