# Supplementary figures and images for: A novel nematode species from the Siberian permafrost shares adaptive mechanisms for cryptobiotic survival with C. elegans dauer larva
Source: PLoS Genet. 2023 Jul 27;19(7):e1010798. doi: 10.1371/journal.pgen.1010798 (PMC10374039; doi:10.1371/journal.pgen.1010798)

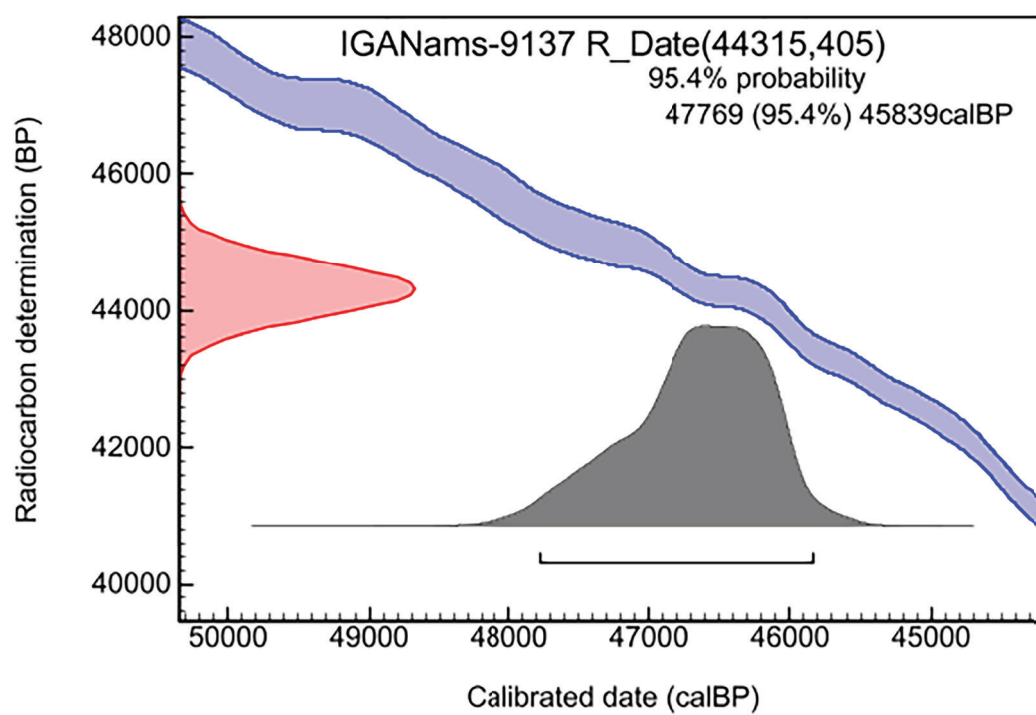

Supplement: S1 Fig — Radiocarbon date (44,315±405 BP) and calibrated age (45,839–47,769 cal BP) of plant material collected from buried borrow P-1320. Radiocarbon ages were converted to calendar age equivalents with the OxCal V.4.4 program using the IntCal20 calibration curve. Pink-shaded area—radiocarbon date with standard deviation; grey-shaded area—radiocarbon date projection on the calibration curve with 95.4% probability. (PDF) [file pgen.1010798.s001.pdf]

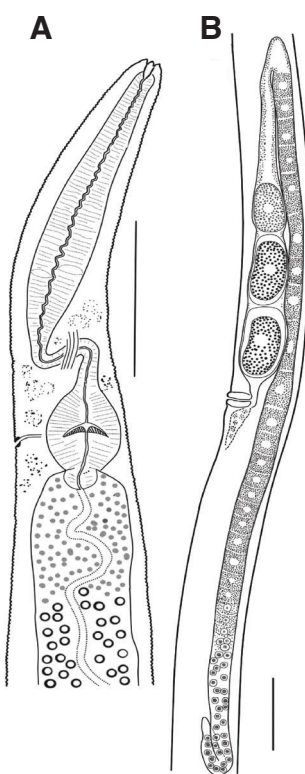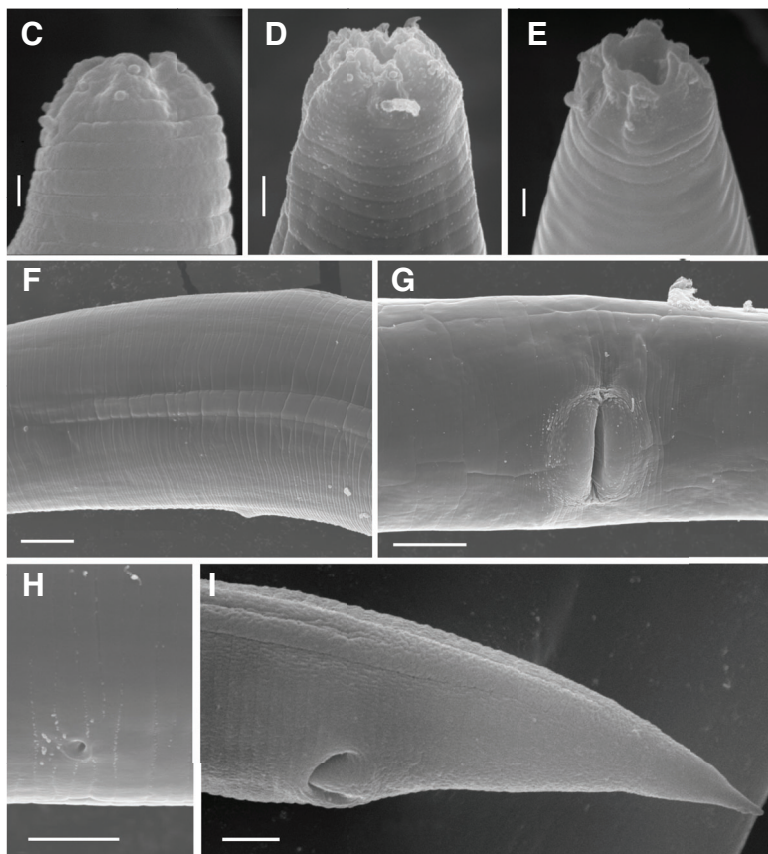

Supplement: S2 Fig — Graphic presentations of holotype (A, B) and SEM pictures (C-I): a) anterior body, B) female reproductive branch, C–E) anterior end of three different female specimens, F) anterior part of the lateral ridge, G) vulva, H) ventral excretory/secretory pore, I) posterior body with anus and lateral ridge. Scale bars: a,b—50 μm, c,i—3 μm, d—2 μm, e –1 μm, f,h—5 μm, g—10 μm,. (PDF) [file pgen.1010798.s002.pdf]

**A**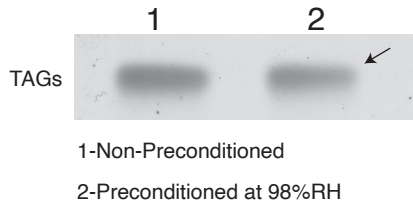**B**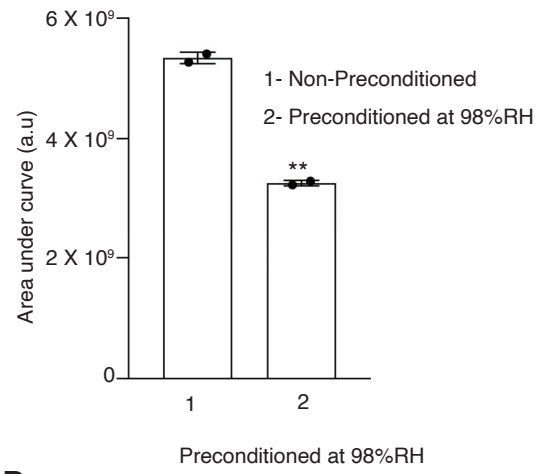**C**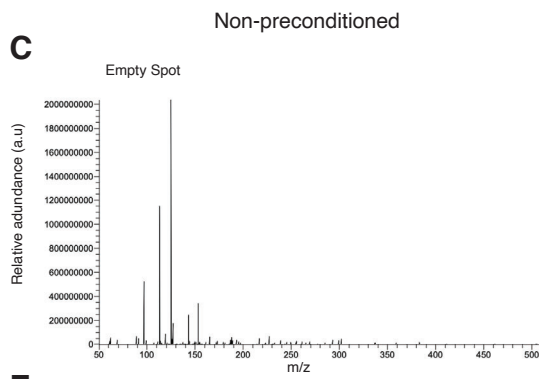**D**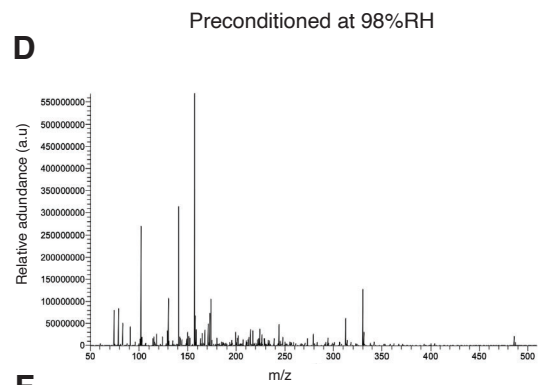**E**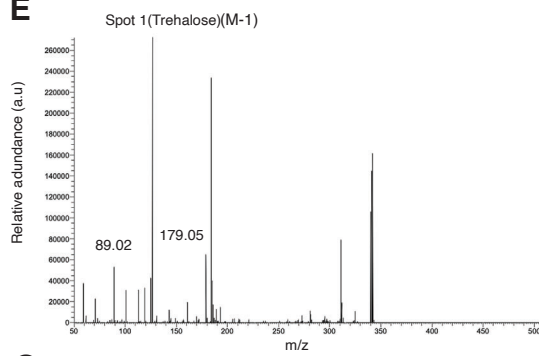**F**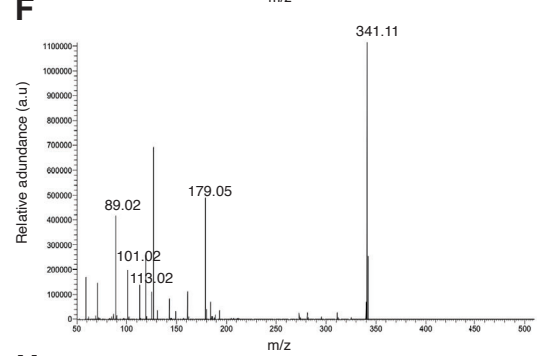**G**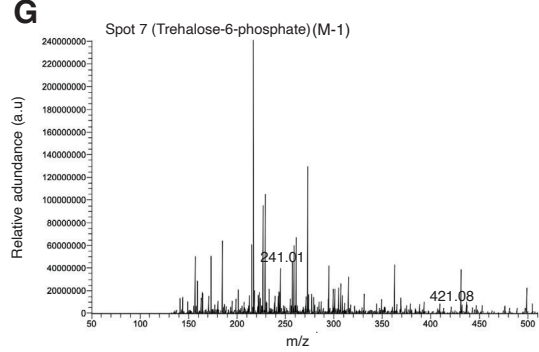**H**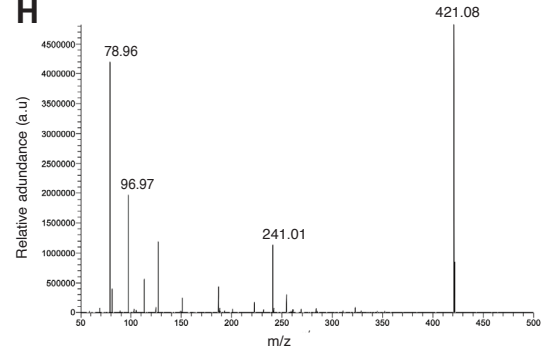

Supplement: S5 Fig — A) 1D-Thin layer chromatography of acetate labelled organic fractions of non-preconditioned (1) and preconditioned (2) P. kolymaensis. B) Mass spectrometric quantification of TAG levels of non-preconditioned (1) and preconditioned (2) P. kolymaensis. Error bars indicate standard deviation of two biological replicates with two technical replicates. Statistical analysis was performed using unpaired t-test with Welch correction **p< 0.001. C-D) non-preconditioned and preconditioned mass spectrum of an empty region, e-f) spot 1 (trehalose), G-H) spot 7 (trehalose-6-phosphate) scraped out and extracted from the 2D-TLC. (PDF) [file pgen.1010798.s005.pdf]

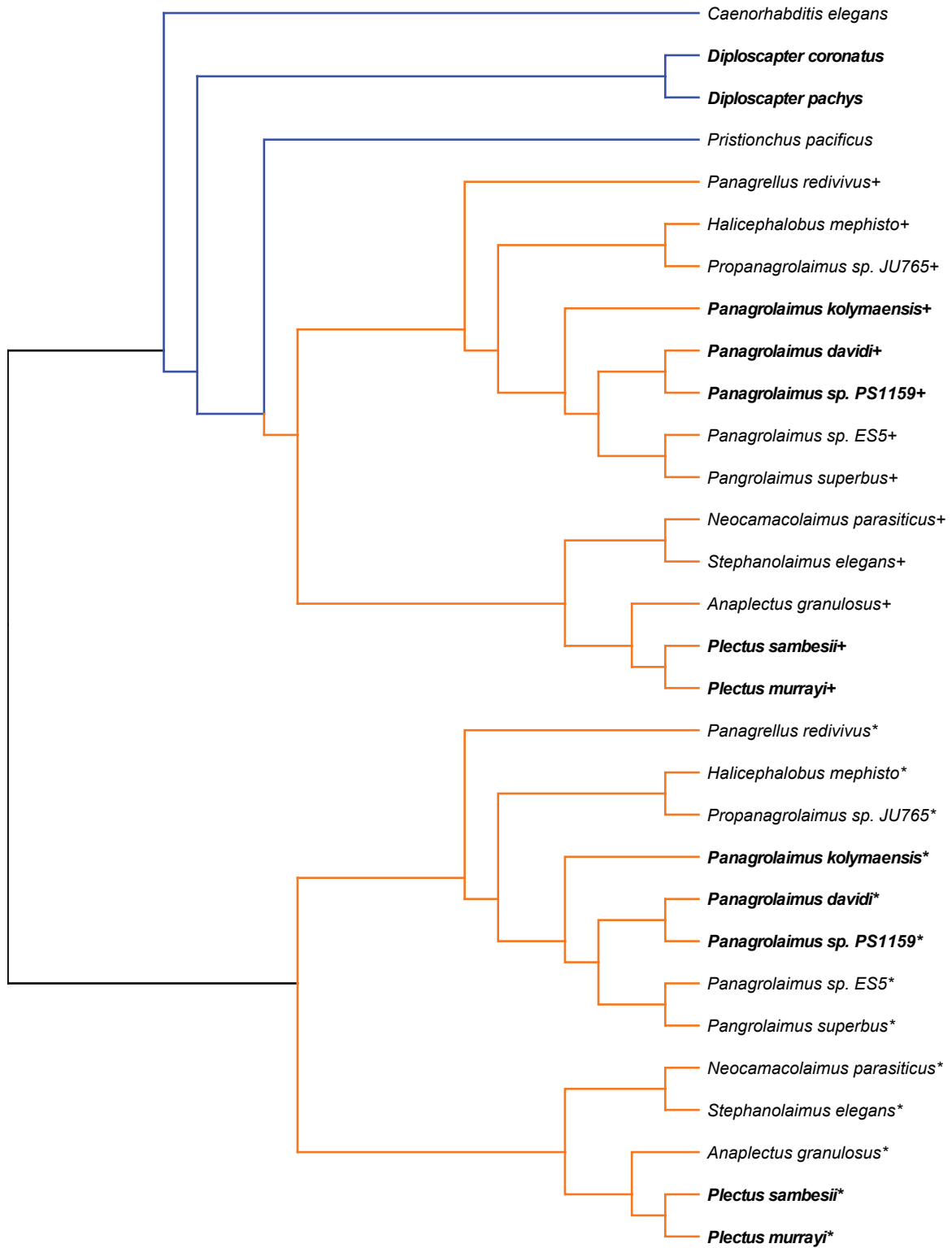

Supplement: S6 Fig — Gene-tree reconciliation was used to determine whether extra sets of proteins across orthogroups originate through auto- or allopolyploidy. Different copies of proteins (designated by ‘+’ and ‘*’) suggest an allopolyploid origin. Parthenogenetic species are highlighted in bold. (PDF) [file pgen.1010798.s006.pdf]
