# Supplementary material for: A novel nematode species from the Siberian permafrost shares adaptive mechanisms for cryptobiotic survival with C. elegans dauer larva
Source: PLoS Genet. 2023 Jul 27;19(7):e1010798. doi: 10.1371/journal.pgen.1010798 (PMC10374039; doi:10.1371/journal.pgen.1010798)
Supplement: S4 Fig — A) Desiccated dauer larvae manifest enhanced survival rate to heat stress (34°C). Error bars indicate standard deviation of two independent experiments with two technical replicates. B) Desiccated dauer larvae display enhances survival rate to anoxia. Error bars indicate standard deviation of two independent experiments with two technical replicates. Statistical comparison was performed by paired two tailed t-test. *p<0.05. C) P. kolymaensis possesses gene orthologs to most genes implicated in dauer formation and metabolism in C. elegans. Black filled circles: Ortholog presence suggested by orthogroup clustering, phylogenetic analysis, and domain architecture. White filled circles: No ortholog found via current analysis (in all cases these C. elegans genes did not cluster with any Panagrolaimus genes in the orthogroup clustering). Label: C. elegans enzyme names and orthogroup that contains that gene according to our orthogroup clustering. (PDF) [file pgen.1010798.s004.pdf]

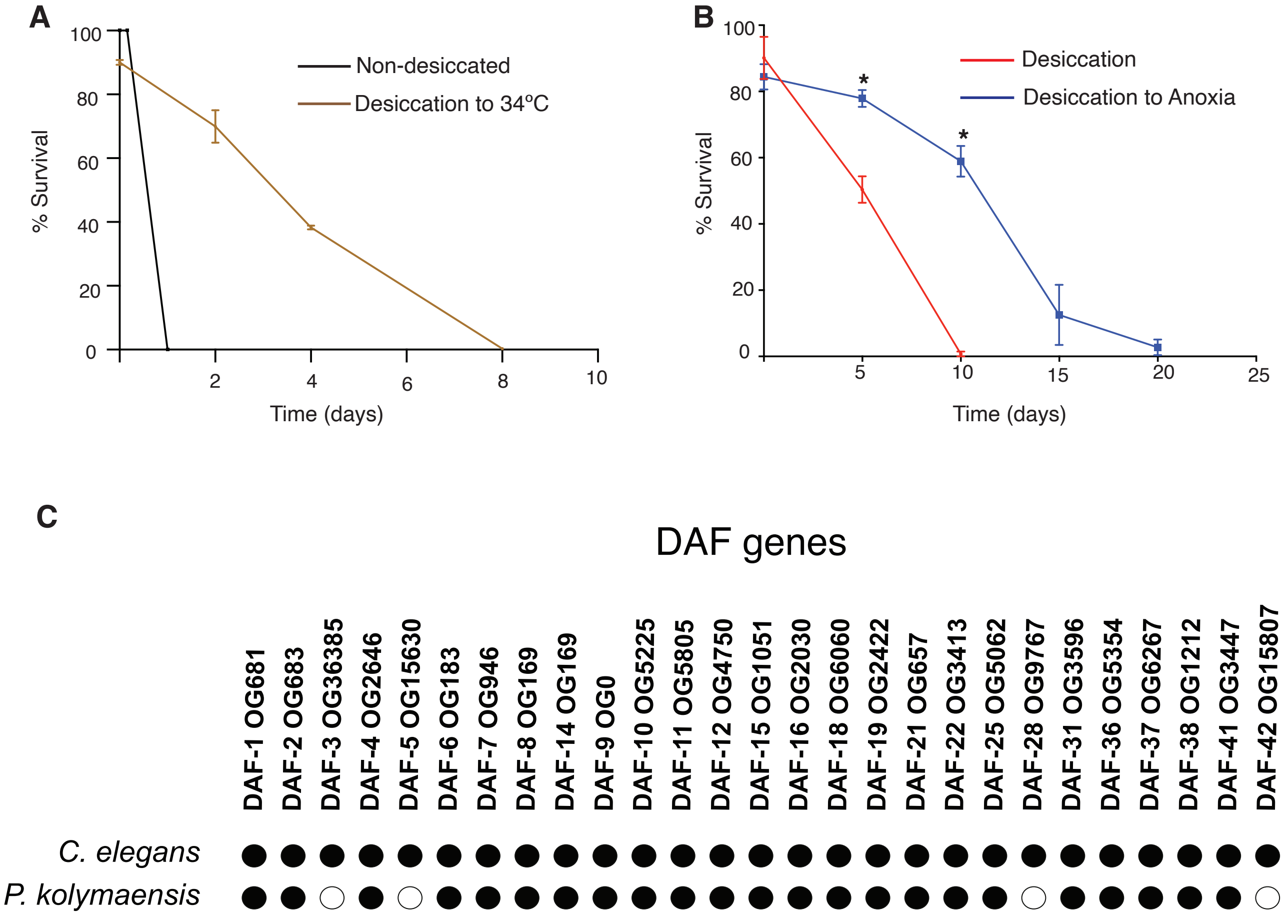
A
100
80
60
40
20
0
% Survival
Non-desiccated
Desiccation to 34ºC
2
4
6
8
10
Time (days)
B
100
80
60
40
20
0
% Survival
Desiccation
Desiccation to Anoxia
*
*
5
10
15
20
25
Time (days)
C
DAF genes
DAF-1 OG681
DAF-2 OG683
DAF-3 OG36385
DAF-4 OG2646
DAF-5 OG15630
DAF-6 OG183
DAF-7 OG946
DAF-8 OG169
DAF-14 OG169
DAF-9 OG0
DAF-10 OG5225
DAF-11 OG5805
DAF-12 OG4750
DAF-15 OG1051
DAF-16 OG2030
DAF-18 OG6060
DAF-19 OG2422
DAF-21 OG657
DAF-22 OG3413
DAF-25 OG5062
DAF-28 OG9767
DAF-31 OG3596
DAF-36 OG5354
DAF-37 OG6267
DAF-38 OG1212
DAF-41 OG3447
DAF-42 OG15807
C. elegans
P. kolymaensis
