# Supplementary material for: A novel nematode species from the Siberian permafrost shares adaptive mechanisms for cryptobiotic survival with C. elegans dauer larva
Source: PLoS Genet. 2023 Jul 27;19(7):e1010798. doi: 10.1371/journal.pgen.1010798 (PMC10374039; doi:10.1371/journal.pgen.1010798)
Supplement: S1 Text — (PDF) [file pgen.1010798.s007.pdf]

S1 Text

Table A: Morphometrics of *P. kolymaensis*

Characters	Holotype female	All the type female specimens				
		n	min–max	mean	SD	CV
Body length, µm	944	19	813–979	896	35.3	3.93
Body diameter at the level of anterior sensilla, µm	8	19	6.5–8	7.4	0.47	6.35
Body diameter at the level of nerve ring, µm	29	19	24–33	29	1.99	6.78
Body diameter at the level of cardia, µm	31	19	30–35	33	1.59	4.88
Body diameter at the level of midbody, µm	44	19	37–44	40	1.82	4.52
Body diameter at the level of anus, µm	24	19	16–27	23	2.25	9.66
a	21.5	19	21–26	22	1.26	5.69
b	6.84	18	5.66–6.84	6.18	0.31	5.02
c	17.8	16	16–21	18	1.52	8.45
c’	2.18	16	1.78–2.58	2.12	0.19	8.96
V. %	61.3	18	57–68	61	2.22	3.67
Procorpus length, µm	98	18	85–103	93	5.71	6.12
Terminal bulb length, µm	26	19	23–33	26	2.48	9.51
Terminal bulb width, µm	20	19	17–21	18	1.13	6.11

Remarks: a – body length divided by maximum body diameter; b – body length divided by length of the pharynx; c – body length divided by tail length; c’ – tail length divided by anal body diameter; CV – coefficient of variation; min–max – range; n – number of individuals measured; SD – standard deviation; V – distance of vulva from anterior end as percentage of body length (%).

Table B: All taxa used for inferring the species tree. Accession numbers for each gene are displayed. Members of *Panagrolaimus* who form a monophyletic clade with definitive *Propanagrolaimus* taxa have been renamed, and are designated with "*".

Taxon	18S Accession number	28S Accession number
Ancestral panagrolaimus	-	-
Panagrolaimus sp. ES6	FJ590961.1	FJ590996.1
Panagrolaimus rigidus	FJ590974.1	FJ591009.1
Panagrolaimus sp. ES2	FJ590962.1	FJ590997.1
Panagrolaimus sp. ES1	FJ590960.1	FJ590995.1
Panagrolaimus superbus	FJ590973.1	FJ591008.1
Panagrolaimus sp. ES5	FJ590972.1	FJ591007.1
Panagrolaimus sp. PS443	FJ590978.1	FJ591013.1
Panagrolaimus sp. DL0180	FJ590990.1	FJ591025.1
Panagrolaimus sp. DL0128	FJ590987.1	FJ591022.1
Panagrolaimus sp. ES3	FJ590963.1	FJ590998.1
Panagrolaimus sp. DL0117	FJ590986.1	FJ591021.1
Panagrolaimus sp. PS1579	FJ590976.1	FJ591011.1
Panagrolaimus sp. PS3966	FJ590983.1	FJ591018.1
Panagrolaimus davidi	FJ590981.1	FJ591016.1
Panagrolaimus sp. PS1159	FJ590977.1	FJ591012.1
Panagrolaimus sp. DL0139	FJ590989.1	FJ591024.1
Panagrolaimus sp. DL0137	FJ590988.1	FJ591023.1
Panagrolaimus sp. DL0072	FJ590985.1	FJ591020.1
Panagrolaimus sp. DL0050	FJ590984.1	FJ591019.1
Panagrolaimus sp. JB051	FJ590971.1	FJ591006.1
Panagrolaimus sp. SN103	FJ590970.1	FJ591005.1
Panagrolaimus sp. JB115	FJ590969.1	FJ591004.1
Panagrolaimus sp. JB131	FJ590968.1	FJ591003.1
Panagrolaimus sp. PS1162	FJ590958.1	FJ590993.1
Panagrolaimus sp. PS1806	FJ590957.1	FJ590992.1
*Propanagrolaimus sp. PS1732**	FJ590959.1	FJ590994.1
*Propanagrolaimus sp. JU765**	FJ590956.1	FJ590991.1*
Propanagrolaimus sp. WTM1	KJ434176.1	KJ434176.1
Propanagrolaimus sp. LC91	KJ434175.1	KJ434175.1
*Propanagrolaimus detritophagus**	FJ590980.1	FJ591015.1
*Propanagrolaimus paetzoldi**	FJ590979.1	FJ591014.1
Panagrellus redivivus	AF083007.1	KU180687.1
Halicephalobus gingivalis	MK087058.1	DQ145685.1
Halicephalobus sp. AA4	MF470238.1	MG051261.1
Halicephalobus sp. FTL-5A-2	MF470221.1	MG051244.1
Halicephalobus sp. G228	MF470235.1	MG051259.1
Halicephalobus sp. KW-1A-7	MF470228.1	MG051253.1
Halicephalobus sp. SRC-14A	MF470211.1	MG051231.1

Halicephalobus mephisto	Mined from genome	Mined from genome
Halicephalobus sp. LD6	FJ590965.1	FJ591000.1
Halicephalobus sp. LD8	FJ590967.1	FJ591002.1
Halicephalobus sp. LD7	FJ590966.1	FJ591001.1
Halicephalobus sp. LD3	FJ590964.1	FJ590999.1
Caenorhabditis briggsae	U13929.1	FJ591017.1

Table C: Estimated repeat content of sequences from *P. kolymaensis* genome assembly.

A total of 22.3% of the *P. kolymaensis* genome assembly consists of repetitive sequences. Simple repeats comprise the largest portion of the repetitive genome.

Repeat class	% of the assembly
SINE	0.11%
LINE	0.23%
LTR	0.73%
DNA	0.97%
Simple repeats	14.30%
Unclassified repeats	7.23%
TOTAL	22.29%

Table D: All 60 genes used for phylogenetic analyses and their best-fit models of sequence evolution.

Gene	Best fit model
ahcy-1	TPM2+F+G4
alh-8	TIM2e+I+G4
aps-3	TIM3+F+R5
arx-2	GTR+F+I+G4
atg-3	TIM3e+I+G4
bud-31	SYM+I+G4
calm-1	SYM+I+G4
ccb-1	TIM2+F+I+G4
cni-1	TIM2+F+I+G4
csq-1	TIM3+F+I+G4
dlc-1	TIM3e+I+G4
dnj-30	TIM3+F+G4
eif-6	TN+F+G4
fbp-1	GTR+F+I+G4
gpa-12	GTR+F+G4
hint-1	GTR+F+I+G4
inf-1	TIM2+F+I+G4
let-522	K2P+G4
let-60	TNe+I+G4
mdh-1	TIM2+F+I+G4
mlt-8	TPM2u+F+I+G4
ndk-1	TIM3+F+R4
nuo-1	TVMe+R4
oig-2	GTR+F+I+G4
oig-3	TVMe+I+G4
pas-4	TNe+I+G4
pbs-3	TIM2+F+R5
pbs-5	TPM2u+F+I+G4
plpr-1	TIM2+F+R4
rab-14	TIM2e+I+G4
rab-7	TIM2e+G4
ran-1	TIM3+F+G4
rap-2	HKY+F+I+G4
ric-4	TIM2e+R4
rpb-10	GTR+F+R5
rpl-12	TIM2+F+I+G4
rpl-15	SYM+I+G4
rpl-2	TNe+I+G4
rpl-26	GTR+F+I+G4
rpl-31	TIM3e+I+G4
rpl-9	HKY+F+I+G4
rps-13	TPM2+F+I+G4
rps-15	TIM2+F+I+G4

rps-20	TIM2e+I+G4
rps-3	GTR+F+I+G4
rps-9	TIM2+F+I+G4
sar-1	TIM2e+I+G4
sftb-1	TNe+G4
snr-7	GTR+F+R5
snx-3	TIM2+F+R4
tin-10	TIM2+F+I+G4
tpi-1	HKY+F+G4
uba-5	GTR+F+R5
ufc-1	TN+F+I+G4
ufm-1	TIM2+F+I+G4
unc-69	TIM3+F+I+G4
usp-46	GTR+F+R4
vha-10	TPM2+F+I+G4
vha-14	TIM2+F+I+G4
vha-4	SYM+R4

Table E: All genomes used for phylogenetic analysis, their class, and the number of genes used for each species are displayed.

Acanthocheilonema viteae	Nematoda	33
Acrobeloides nanus	Nematoda	55
Ancylostoma caninum	Nematoda	68
Ancylostoma ceylanicum	Nematoda	87
Ancylostoma duodenale	Nematoda	57
Angiostrongylus cantonensis	Nematoda	65
Angiostrongylus costaricensis	Nematoda	78
Anisakis simplex	Nematoda	44
Ascaris lumbricoides	Nematoda	45
Ascaris suum	Nematoda	46
Brugia malayi	Nematoda	92
Brugia pahangi	Nematoda	40
Brugia timori	Nematoda	13
Bursaphelenchus xylophilus	Nematoda	57
Caenorhabditis elegans	Nematoda	133
Caenorhabditis remanei	Nematoda	129
Caenorhabditis sp34	Nematoda	131
Dictyocaulus viviparus	Nematoda	76
Diploscapter coronatus	Nematoda	104
Diploscapter pachys	Nematoda	91
Dirofilaria immitis	Nematoda	37
Ditylenchus destructor	Nematoda	44
Ditylenchus dipsaci	Nematoda	30
Dracunculus medinensis	Nematoda	34
Elaeophora elaphi	Nematoda	37
Enterobius vermicularis	Nematoda	42
Globodera pallida	Nematoda	27
Globodera rostochiensis	Nematoda	37
Gongylonema pulchrum	Nematoda	10
Haemonchus contortus	Nematoda	103
Haemonchus placei	Nematoda	80
Halicephalobus mephisto	Nematoda	63
Heligmosomoides polygyrus	Nematoda	77
Heterodera glycines	Nematoda	37
Heterorhabditis bacteriophora	Nematoda	17
Litomosoides sigmodontis	Nematoda	42
Loa loa	Nematoda	129
Meloidogyne arenaria	Nematoda	41
Meloidogyne enterolobii	Nematoda	24
Meloidogyne floridensis	Nematoda	11
Meloidogyne graminicola	Nematoda	20
Meloidogyne hapla	Nematoda	24
Meloidogyne incognita	Nematoda	37
Meloidogyne javanica	Nematoda	39
Mesorhabditis belari	Nematoda	91

Micoletzkya japonica	Nematoda	65
Necator americanus	Nematoda	130
Nippostrongylus brasiliensis	Nematoda	74
Oesophagostomum dentatum	Nematoda	42
Onchocerca flexuosa	Nematoda	20
Onchocerca ochengi	Nematoda	33
Onchocerca volvulus	Nematoda	46
Oscheius tipulae	Nematoda	97
Panagrellus redivivus	Nematoda	65
Panagrolaimus superbus	Nematoda	37
Panagrolaimus sp. DL0137	Nematoda	59
Panagrolaimus sp. ES5	Nematoda	55
Panagrolaimus sp. PS1159	Nematoda	54
Panagrolaimus sp. PS1579	Nematoda	52
Parapristionchus giblindavisi	Nematoda	62
Parascaris univalens	Nematoda	59
Parastrongyloides trichosuri	Nematoda	47
Plectus sambesii	Nematoda	49
Pristionchus arcanus	Nematoda	69
Pristionchus entomophagus	Nematoda	65
Pristionchus exspectatus	Nematoda	68
Pristionchus fissidentatus	Nematoda	72
Pristionchus japonicus	Nematoda	74
Pristionchus maxplancki	Nematoda	70
Pristionchus mayeri	Nematoda	65
Pristionchus pacificus	Nematoda	74
Propanagrolaimus sp. JU765	Nematoda	68
Romanomermis culicivorax	Nematoda	13
Soboliphyme baturini	Nematoda	11
Steinernema carpocapsae	Nematoda	66
Steinernema feltiae	Nematoda	69
Steinernema glaseri	Nematoda	67
Steinernema monticolum	Nematoda	61
Steinernema scapterisci	Nematoda	73
Strongyloides papillosus	Nematoda	46
Strongyloides ratti	Nematoda	44
Strongyloides stercoralis	Nematoda	38
Strongyloides venezuelensis	Nematoda	43
Strongylus vulgaris	Nematoda	20
Syphacia muris	Nematoda	39
Teladorsagia circumcincta	Nematoda	43
Thelazia callipaeda	Nematoda	40
Toxocara canis	Nematoda	49
Trichinella britovi	Nematoda	13
Trichinella murrelli	Nematoda	12
Trichinella nativa	Nematoda	11
Trichinella nelsoni	Nematoda	13
Trichinella papuae	Nematoda	13

Trichinella patagoniensis	Nematoda	14
Trichinella spiralis	Nematoda	103
Trichinella zimbabwensis	Nematoda	11
Trichuris muris	Nematoda	17
Trichuris suis	Nematoda	11
Trichuris trichiura	Nematoda	15
Wuchereria bancrofti	Nematoda	29
Macrostomum lignano	Platyhelminthes	11

Supplementary methods

Sampling site

The sampling site was in the Kolyma Lowland of northeast Siberia, Russia, in the continuous permafrost zone (Fig 1A). Sample P-1320 was collected by Dr.S.Gubin in summer 2002 from the frozen wall of the outcrop Duvanny Yar located on the right bank of the Kolyma River (68° 37′ 739″ N, 159° 11′ 678″ E). The outcrop Duvanny Yar is a cliff about 9 km long and about 55 m high above the river water level and is described in detail as a key section of late Pleistocene deposits (Ice complex) in the northeastern Arctic (Fig 1B)[1][2] These deposits accumulated syngenetically, i.e., simultaneously freezing from below and never thawed since the moment of formation. Frozen silty sediments include sandy alluvial layers, peat lenses, buried paleosols, Pleistocene rodent burrows and wide variety of plant and vertebrate fossils (Fig 1C)[3] [4] . The permafrost deposits have high ice content (35–80%) and mean annual temperature varies from -3°C to -11°C depending on the depth[5]. Maximal depth of seasonal thawing of permafrost-affected soils at the sites does not exceed 70 cm, while permafrost thickness is over 500 meters[2].

Fossil rodent burrows

Studied burrow P-1320 was taken from the frozen outcrop wall at a depth of about 40 m below the surface and about 11 m above river water level in undisturbed and never thawed late Pleistocene permafrost deposits. The fossil burrow left by arctic gophers *Citellus (Urocitellus) parrii* Richardson, 1825, consists of entrance tunnel and a large nesting chamber up to 25 cm in diameter (Fig 1D)[6]. The material of fossil burrows represented by the well-preserved remnants of herbaceous plants, included seeds of higher plants, rodent bones and coprolites, insects, feathers, hairs of large animals and other material brought from the surface, and are part of the biogeocenoses formed in the immediate vicinity of the rodent's dwelling. Biological material in the ancient burrows is quite well preserved due to fastest burial and freezing into aggrading permafrost sediment[4].

Sampling procedure

Sampling, storage and transportation of the frozen samples under sterile conditions, as well as contamination testing were performed using protocols that have previously been described for microbiological purposes[7] [8] [9] [10]. Samples were taken from the frozen outcrops wall. Melting parts of burrows were removed until the unthawed layer exposed. The whole ice-

cemented chamber was removed from the frozen sediment, placed in the plastic bag and kept frozen during field and transportation to the laboratory in Pushchino. In the laboratory surface of frozen chamber was cleaned, the internal part was aseptically separated and divided into pieces inside a laminar airflow cabinet, placed in sterile plastic bags and stored at -20°C until analyses.

Age of *P. kolymaensis*

According the radiocarbon dating, stratigraphic and spore-pollen analysis the permafrost deposits of Duvanny Yar were accumulated under tundra-steppe landscape during the marine isotopic stages (MIS) 2 and 3 (15.5 - 55 kya BP)[2]. Earlier, more than 30 similar burrows of fossil rodents were described in the layer of permafrost sediments at depths of 10-20 m above river level[3,4]. Series of radiocarbon dating of animal and plant material from permafrost sediments, buried borrows and paleosols from this layer were obtained previously, amounting between about 30 and up to 50 kyr BP depending on the depth[2,10–12] . In the current study the Accelerator Mass Spectrometry (AMS) radiocarbon dating of plant material obtained from the burrow P-1320 was provided in the Laboratory for Radiocarbon Dating and Electron Microscopy, Institute of Geography, RAS, and determined a direct ^{14}C age of 44,315±405 BP ($IGAN_{AMS}$ 9137) and calibrated age range is 45,839 – 47,769 cal BP (95.4% probability) (Fig S1).

It was shown that below the seasonally thawing layer, which is no more than 0.7 m in the study sites, any migration or thermal diffusion of bacterial cells with unfrozen water films within the stratum is precluded[7,8,13,14]. Permafrost deposits contained burrows were firmly cemented by ice and showed no signs of degradation or thawing of permafrost in the past. Thus, the vertical movement of modern microorganisms and contamination syncryogenic strata is impossible and age of biota in corresponds to the age of permafrost sediments[15]. Natural melting of permafrost leads to the destruction of the outcrops wall during the warm season at a rate of about a meter per month, exposing the new frozen deposits and continually revealing new buried paleontological objects, for example well-preserved mammal and other ancient animal mummies.

Nematode isolation and culturing

Revitalization of cultivable ancient nematodes was observed during enrichment cultivation with *E. coli* strain OP50 as a food source. Samples of about 1cm³ were taken from the inner part of the frozen burrows under sterile conditions (inside laminar air flow cabinet) and

resuspended in mineral liquid Prescott–James medium (PJM) into Petri dishes[16]. To avoid drying and cross-contamination, the plates were wrapped with Parafilm and cultivated at 20ºC for several weeks. The soil suspensions were observed every 2-3 days in closed plates using an inverted light microscope Nikon Eclipse E100 to find the revived organisms until motile nematodes were detected. Clonal strains were established from individual nematodes which were picked from the soil suspension, transferred to fresh liquid PJM plates, and grown at 20ºC using *E. coli* strain OP50 as a food source. Strain *E. coli* was grown in the tryptic soy broth agar (1/2 TSB) overnight at +35°C. Agar medium with lawn of *E. coli* was cut into pieces of about 1cm² in size and placed in a liquid PJM plate with nematode cultures. Further strains of *P. kolymaensis* are maintained on liquid PJM and Nematode Growth Medium (NGM) agar plates at +18°C[17].The stages of growing ancient nematodes from a frozen sample were repeated in the control lines. Laboratory bulk cultures and 10 strains (lineages) derived from isolates are reseeded every 2 months and maintained under laboratory conditions for more than 4 years until now. Morphological and phylogenetical investigations carried out used Pn2-1 strain.

Light and scanning electron microscopy

Nematodes were taken from a long-term culture *Pn2-1* strain. The specimens were collected from NGM plates and fixed in 4% formalin solution for about four hours and after that were picked into a Syracuse glass filled with mixture of glycerin, 95% ethanol, and distilled water (1:29:70). The glass were placed in a drying oven set to 40°C for 1–2 days until completely dehydrated, as in the glycerin-ethanol method[18]. Specimens were identified, measured, photographed, and drawn under a Leica DM5000 light microscope equipped with Leica Application Suite Version 3.8.0 software and a Leica DFC 425 C digital camera. All the measured sizes are given in µm. For scanning electron microscopy, formalin-fixed specimens were dehydrated in a series: 40% ethanol, 70% ethanol, 95% ethanol, 95% ethanol + acetone (50:50), acetone I, and acetone II, and then critical point dried. Once dried, specimens were mounted on a stub to be coated with platinum–palladium alloy and examined with Cam Scan S-2.

Phylogenomic Analyses

To explore the phylogenetic position of the novel species 18S and 28S rRNA gene sequences from 43 taxa across the *Propanagrolaimus*, *Panagrolaimus*, *Panagrellus* and *Halicephalobus* genera were downloaded from Genbank[19], with sequences from *Caenorhabditis briggsae* used as an outgroup (Table B inS1 Text). Both genes were identified in *Halicephalobus mephisto* (BioProject PRJNA528747) using BLAST[20] and a closely related reference (*Halicephalobus sp. LD6*). Nucleotide sequence alignments were generated using MAFFT L-INS-I[21] (v7.475). Multiple sequence alignments were manually curated and concatenated into a supermatrix[22], with a species tree inferred using maximum likelihood via IQTREE[23]. This supermatrix was partitioned using best-fit model of sequence evolution per gene inferred using ModelFinder[24] with 1000 bootstrap pseudo replicates used to estimate nodal support.

To further expand on the phylogenetic representation, 60 genes present in the novel species that had representative clusters shared with *Brugia malayi*, *Caenorhabditis elegans*, *Caenorhabditis remanei*, *Loa loa*, *Necator americanus* and *Trichinella spiralis* on OrthoDB[25] (minimum of 1 gene per species for the cluster to have been considered, minimum of 10 genes per species needed for inclusion) were used for phylogenetic analysis (Table D inS1 Text). Genome assemblies for an additional 88 nematode species and an outgroup platyhelminth (*Macrostomum lignano*) were downloaded from the WormBase Parasite[26] database (Table E in S1 Text). Additional assemblies the *Panagrolaimus* genera[27], *Panagrolaimus superbus* (BioProject: PRJEB32708), *Panagrolaimus* sp. DL0137 (BioSample: SAMN06329917), *Panagrolaimus* sp. ES5 (BioSample: SAMN06329914), *Panagrolaimus* sp. PS1159 (BioSample: SAMN06329915), *Panagrolaimus* sp. PS1579 (BioSample: SAMN06329916) and the species *Propanagrolaimus* sp. JU765 (BioSample: SAMN06329918) were also included (n=100 nematode species). As these genomes were not present in the OrthoDB clusters, possible orthologs were identified by mapping *C. elegans* sequences for all genes to each genome, with a minimum threshold of 70% sequence identity and 75% gene coverage for a hit to be considered a putative ortholog. Given the potential for sequence quality negatively impacting sequence alignment, the frame-shift-aware alignment software MACSE[28] was used to generate multiple sequence alignments using the '*alignSequences*' function. The phylogeny was inferred using the supertree method with IQTREE as described above.

To estimate the rate of amino acid sequence diversity, the number of substitutions per site between the novel species, *Panagrolaimus* sp. PS1159 and *Panagrolaimus* sp. ES5, gene trees for each orthogroup were inferred using IQTREE. Distances between branches for all orthogroups were determined using the R package '*ape*'[29].

References

1. Wetterich S, Tumskoy V, Rudaya N, Andreev AA, Opel T, Meyer H, et al. Ice Complex formation in arctic East Siberia during the MIS3 Interstadial. Quaternary Science Reviews. 2014;84: 39–55. doi:10.1016/j.quascirev.2013.11.009

2. Murton JB, Goslar T, Edwards ME, Bateman MD, Danilov PP, Savvinov GN, et al. Palaeoenvironmental Interpretation of Yedoma Silt (Ice Complex) Deposition as Cold-Climate Loess, Duvanny Yar, Northeast Siberia: Palaeoenvironmental Interpretation of Yedoma Silt, Duvanny Yar. Permafrost and Periglac Process. 2015;26: 208–288. doi:10.1002/ppp.1843

3. Gubin SV, Maximovich SV, Zanina OG, Stakhov VL. Morphogenetics of plant remains from paleosols and rodent burrows buried in permafrost of the Late Pleistocene (32-28,000 BP). Plant Archaeogenetics. 2011. pp. 11–21.

4. Zanina OG, Gubin SV, Kuzmina SA, Maximovich SV, Lopatina DA. Late-Pleistocene (MIS 3-2) palaeoenvironments as recorded by sediments, palaeosols, and ground-squirrel nests at Duvanny Yar, Kolyma lowland, northeast Siberia. Quaternary Science Reviews. 2011;30: 2107–2123. doi:10.1016/j.quascirev.2011.01.021

5. Romanovsky VE, Drozdov DS, Oberman NG, Malkova GV, Kholodov AL, Marchenko SS, et al. Thermal state of permafrost in Russia. Permafrost Periglac Process. 2010;21: 136–155. doi:10.1002/ppp.683

6. Faerman M, Bar-Gal GK, Boaretto E, Boeskorov GG, Dokuchaev NE, Ermakov OA, et al. DNA analysis of a 30,000-year-old Urocitellus glacialis from northeastern Siberia reveals phylogenetic relationships between ancient and present-day arctic ground squirrels. Sci Rep. 2017;7: 42639. doi:10.1038/srep42639

7. Shi L, Liu X, Shi L, Stinson HT, Rowlette J, Kahl LJ, et al. Mid-infrared metabolic imaging with vibrational probes. Nature Methods. 2020;17.

8. Juck DF, Whissell G, Steven B, Pollard W, McKay CP, Greer CW, et al. Utilization of fluorescent microspheres and a green fluorescent protein-marked strain for assessment of microbiological contamination of permafrost and ground ice core samples from the

Canadian High Arctic. Appl Environ Microbiol. 2005;71: 1035–1041. doi:10.1128/AEM.71.2.1035-1041.2005

9. Gilichinsky DA, Wilson GS, Friedmann EI, Mckay CP, Sletten RS, Rivkina EM, et al. Microbial Populations in Antarctic Permafrost: Biodiversity, State, Age, and Implication for Astrobiology. Astrobiology. 2007;7: 275–311. doi:10.1089/ast.2006.0012

10. Legendre M, Bartoli J, Shmakova L, Jeudy S, Labadie K, Adrait A, et al. Thirty-thousand-year-old distant relative of giant icosahedral DNA viruses with a pandoravirus morphology. Proceedings of the National Academy of Sciences. 2014;111: 4274–4279. doi:10.1073/pnas.1320670111

11. Lopatina DA, Zanina OG. Conditions of Formation of Paleosols during MIS 3 in the Kolyma Lowland according to Palynological and Phytolith Methods. Stratigr Geol Correl. 2020;28: 330–341. doi:10.1134/S0869593820030065

12. Yashina S, Gubin S, Maksimovich S, Yashina A, Gakhova E, Gilichinsky D. Regeneration of whole fertile plants from 30,000-y-old fruit tissue buried in Siberian permafrost. Proceedings of the National Academy of Sciences. 2012;109: 4008–4013. doi:10.1073/pnas.1118386109

13. Shi T, Reeves RH, Gilichinsky DA, Friedmann EI. Characterization of Viable Bacteria from Siberian Permafrost by 16S rDNA Sequencing. Microbial Ecology. 1997;33: 169–179. doi:10.1007/s002489900019

14. Gilichinskiy DA, Khlebnikova GM, Zvyagintsev DG, Fedorov-Davydov DG, Kudryavtseva NN. MICROBIOLOGY OF SEDIMENTARY MATERIALS IN THE PERMAFROST ZONE. International Geology Review. 1989;31: 847–858. doi:10.1080/00206818909465938

15. Abramov A, Vishnivetskaya T, Rivkina E. Are permafrost microorganisms as old as permafrost? FEMS Microbiology Ecology. 2021;97: fiaa260. doi:10.1093/femsec/fiaa260

16. Prescott DM, James TW. CULTURING OF AMOEBA PROTEUS ON TETRAHYMENA. Experimental Cell Research. 1955;8: 256–258.

17. Brenner S. THE GENETICS OF CAENORHABDITIS ELEGANS. Genetics. 1974;77: 71–94. doi:10.1093/genetics/77.1.71

18. Seinhorst JW. A Rapid Method for the Transfer of Nematodes From Fixative To Anhydrous Glycerin. Nematologica. 1959;4: 67–69.

19. Clark K, Karsch-Mizrachi I, Lipman DJ, Ostell J, Sayers EW. GenBank. Nucleic Acids Res. 2016;44: D67–D72. doi:10.1093/nar/gkv1276

20. AltschuP SF, Gish W, Miller W, Myers EW, Lipman DJ. Basic Local Alignment Search Tool. : 8.

21. Katoh K, Standley DM. MAFFT Multiple Sequence Alignment Software Version 7: Improvements in Performance and Usability. Molecular Biology and Evolution. 2013;30: 772–780. doi:10.1093/molbev/mst010

22. Kück P, Meusemann K. FASconCAT: Convenient handling of data matrices. Molecular Phylogenetics and Evolution. 2010;56: 1115–1118. doi:10.1016/j.ympev.2010.04.024

23. Nguyen L-T, Schmidt HA, von Haeseler A, Minh BQ. IQ-TREE: A Fast and Effective Stochastic Algorithm for Estimating Maximum-Likelihood Phylogenies. Molecular Biology and Evolution. 2015;32: 268–274. doi:10.1093/molbev/msu300

24. Kalyaanamoorthy S, Minh BQ, Wong TKF, von Haeseler A, Jermiin LS. ModelFinder: fast model selection for accurate phylogenetic estimates. Nat Methods. 2017;14: 587–589. doi:10.1038/nmeth.4285

25. Kriventseva EV, Kuznetsov D, Tegenfeldt F, Manni M, Dias R, Simão FA, et al. OrthoDB v10: sampling the diversity of animal, plant, fungal, protist, bacterial and viral genomes for evolutionary and functional annotations of orthologs. Nucleic Acids Research. 2019;47: D807–D811. doi:10.1093/nar/gky1053

26. Howe KL, Bolt BJ, Shafie M, Kersey P, Berriman M. WormBase ParaSite − a comprehensive resource for helminth genomics. Molecular and Biochemical Parasitology. 2017;215: 2–10. doi:10.1016/j.molbiopara.2016.11.005

27. Schiffer PH, Danchin EGJ, Burnell AM, Creevey CJ, Wong S, Dix I, et al. Signatures of the Evolution of Parthenogenesis and Cryptobiosis in the Genomes of Panagrolaimid Nematodes. iScience. 2019;21: 587–602. doi:10.1016/j.isci.2019.10.039

28. Ranwez V, Douzery EJP, Cambon C, Chantret N, Delsuc F. MACSE v2: Toolkit for the Alignment of Coding Sequences Accounting for Frameshifts and Stop Codons. Wilke C, editor. Molecular Biology and Evolution. 2018;35: 2582–2584. doi:10.1093/molbev/msy159

29. Paradis E, Claude J, Strimmer K. APE: Analyses of Phylogenetics and Evolution in R language. Bioinformatics. 2004;20: 289–290. doi:10.1093/bioinformatics/btg412
