# Supplementary material for: A novel nematode species from the Siberian permafrost shares adaptive mechanisms for cryptobiotic survival with C. elegans dauer larva
Source: PLoS Genet. 2023 Jul 27;19(7):e1010798. doi: 10.1371/journal.pgen.1010798 (PMC10374039; doi:10.1371/journal.pgen.1010798)
Supplement: S1 Orthology analysis — (PDF) [file pgen.1010798.s015.pdf]

Supplementary file – ORTHOLOGY_ANALYSIS

All phylogenies were generated with IQtree2 with 1000 bootstraps (-bb option). The scale bar corresponds to 0.1 estimated amino acid substitutions per site.

Anaplectus granulosus – ANAGRA, *Caenorhabditis elegans* – CAEELE, *Diploscapter coronatus* – DIPCOR, *Diploscapter pachys* – DIPPAC, *Halicephalobus mephisto* – HALMEP, *Neocamacolaimus parasiticus* – NEOPAR, *Panagrellus redivivus* – PANRED, *Panagrolaimus davidi* – PANDAV, *Panagrolaimus kolymaensis* – HLNpanKol1, *Panagrolaimus* sp. ES5 – PANES5, *Panagrolaimus* sp. PS1159 – PANPS1159, *Panagrolaimus superbus* – PANSUP, *Plectus murrayi* – PLEMUR, *Plectus sambesii* – PLESAM, *Plectus* sp. (from Permafrost) – HLNpleKol1, *Pristionchus pacificus* – PRIPAC, *Propanagrolaimus* sp. JU765 – PROJU765, *Stephanolaimus elegans* – STEELE.

pp. 2-3: Trehalose synthesis genes

pp. 4-20: TCA cycle

p. 21: Glyoxylate shunt

pp. 22-38: Glycolysis / Gluconeogenesis

pp. 39-40: Polyamine synthesis

pp. 41-62: Dauer genes

Trehalose synthesis

TPS-1 / TPS-2

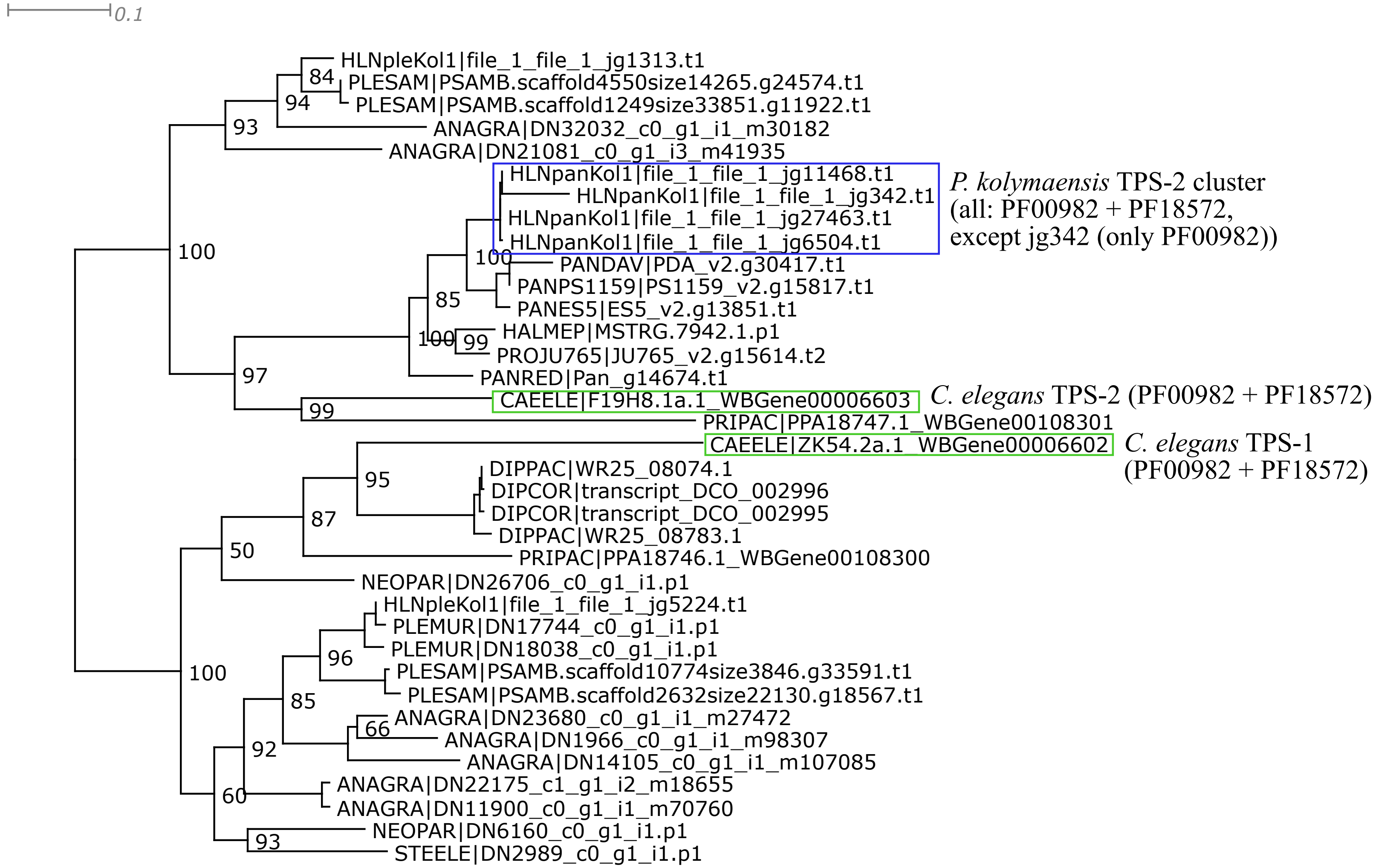

Trimal -automated1 function; one long-branched sequence (*D. pachys*) manually removed afterwards;
IQtree2 ML phylogeny best-fit model according to BIC: LG+I+G4
Almost all plectid sequences that cluster with *C. elegans* TPS-1 do have large gaps in the alignment and were removed at first;
Then the clustering of the one remaining plectid sequence not so clear, therefore, the short sequences were left in the phylogeny.
Panagrolaimids appear to encode only TPS-2.

GOB-1

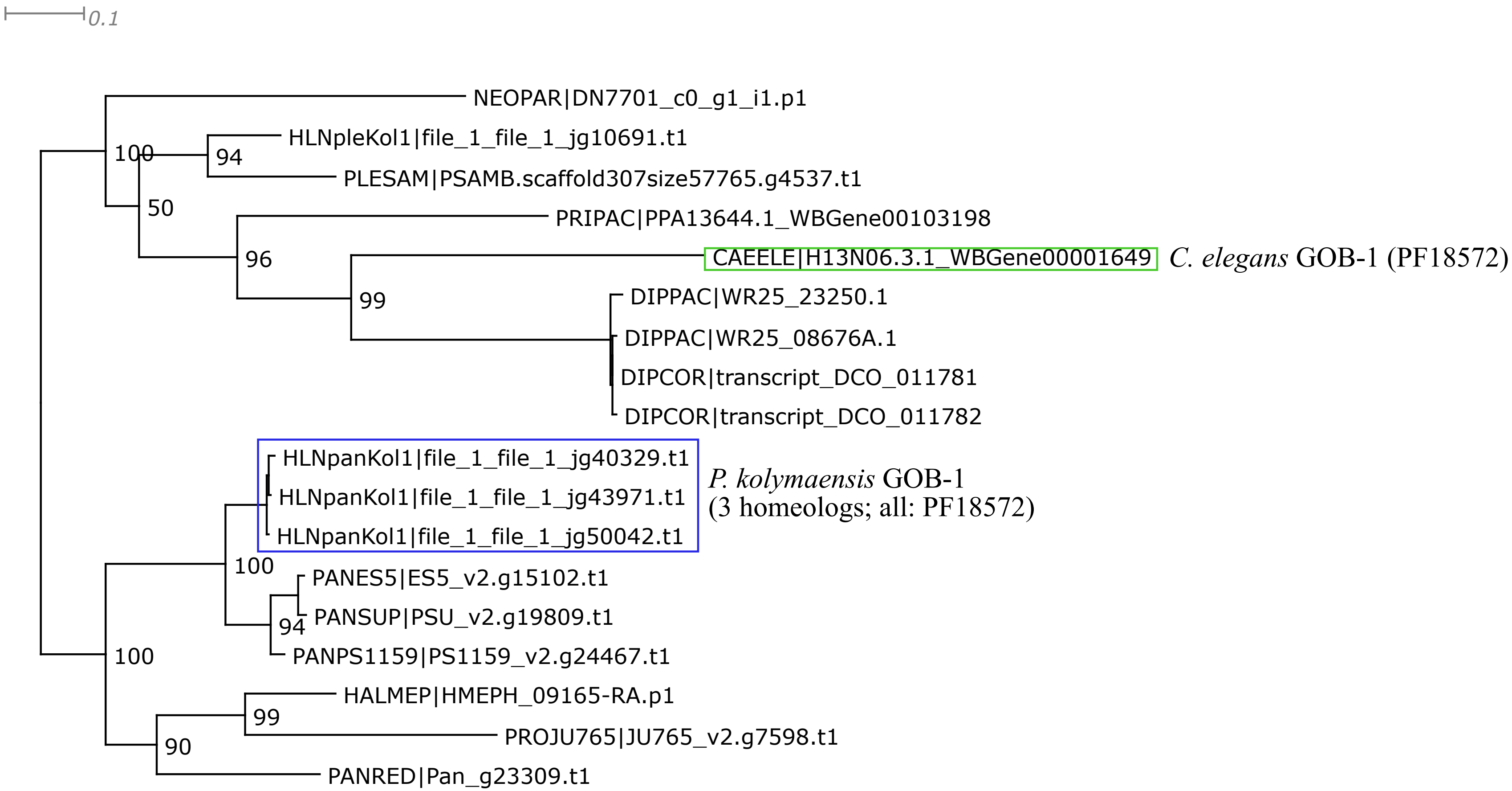

Trimal -automated1 function; short or spurious sequences manually removed afterwards;
IQtree2 ML phylogeny best-fit model according to BIC: LG+G4

TCA Cycle

CTS-1

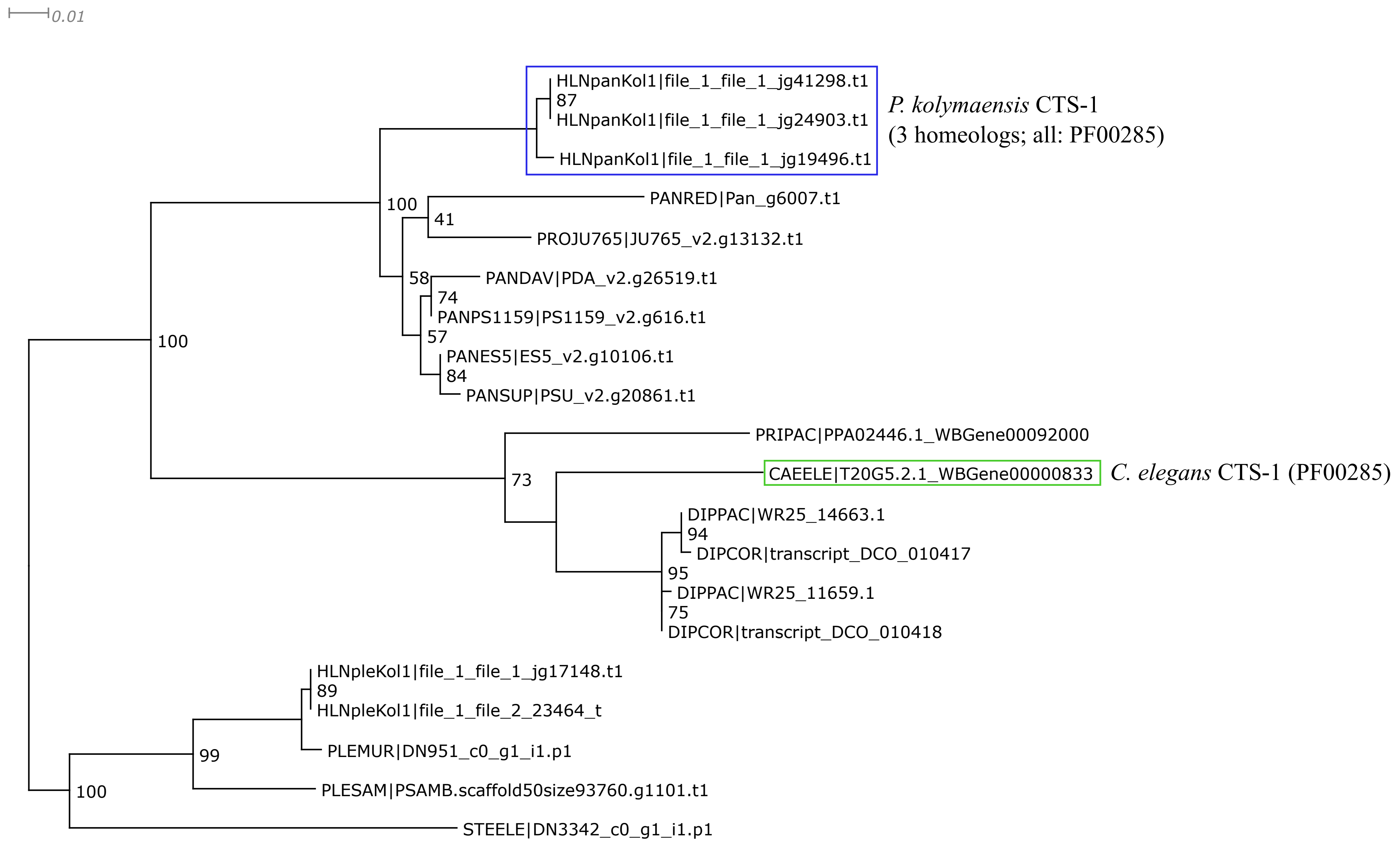

Trimal -automated1 function; short or spurious sequences manually removed afterwards;
IQtree2 ML phylogeny best-fit model according to BIC: LG+G4

ACO-1

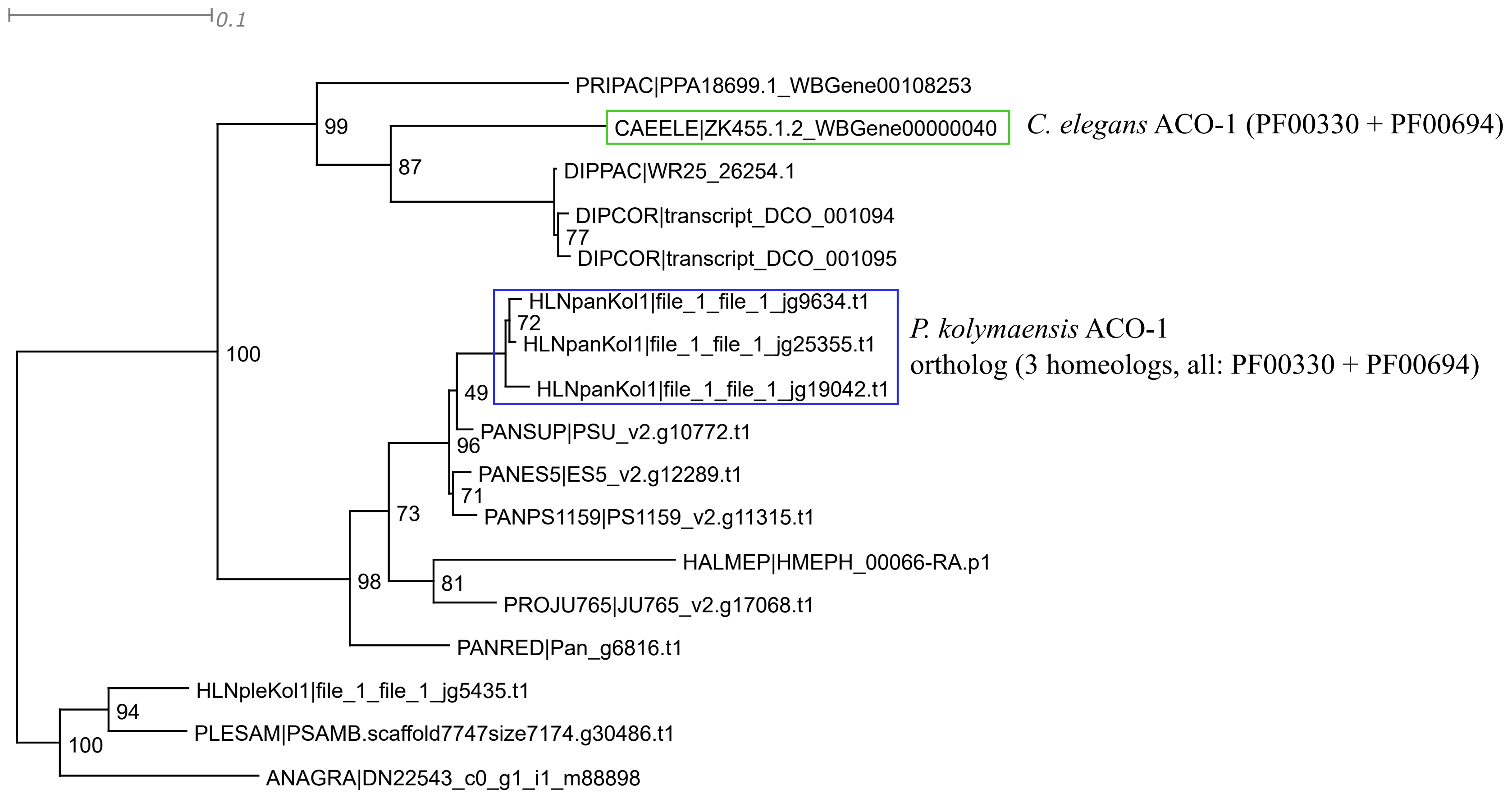

Trimal -automated1 function; short or spurious sequences manually removed afterwards;
IQtree2 ML phylogeny best-fit model according to BIC: LG+I+G4

ACO-2

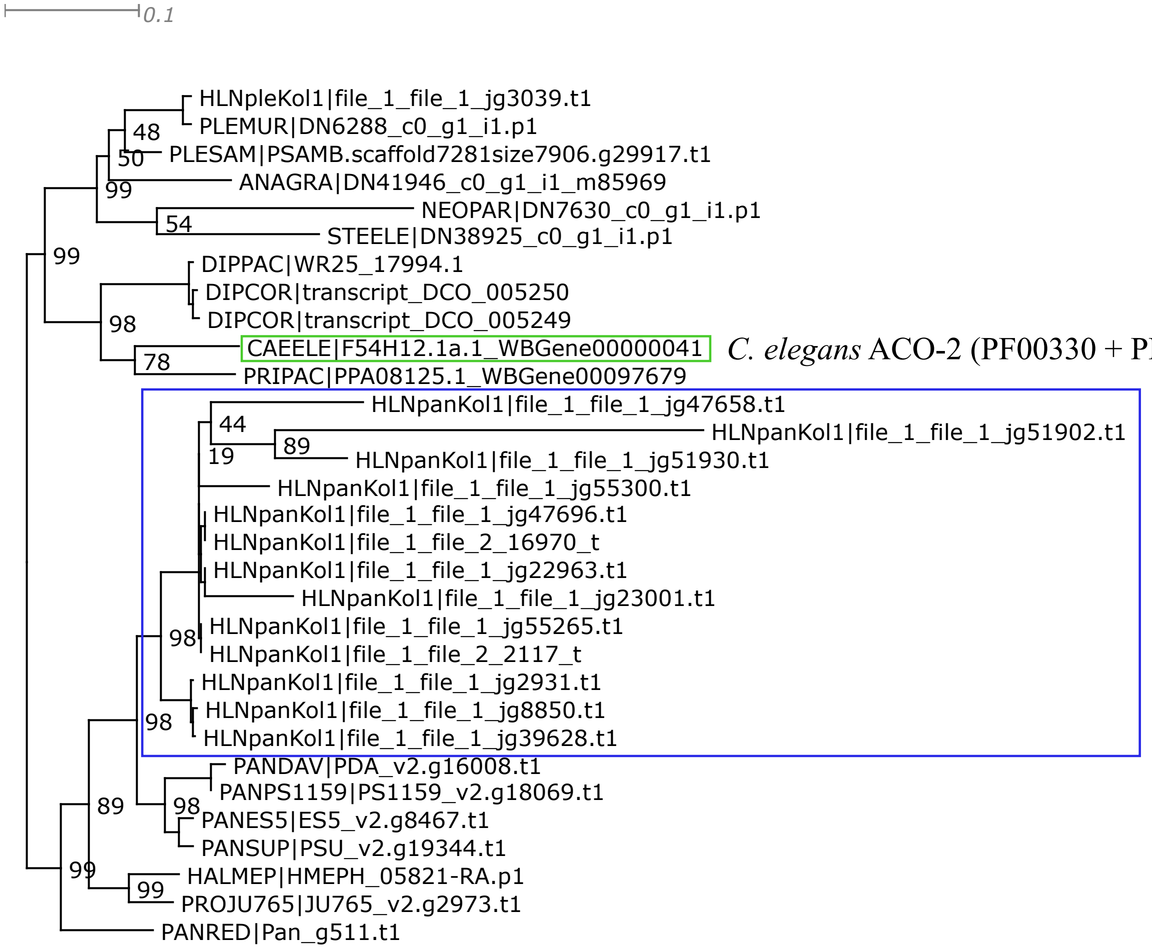

C. elegans ACO-2 (PF00330 + PF00694)

P. kolymaensis ACO-2 expansion
all PF00330 + PF00694, except jg51902 (PF00330 + PF00578), jg51930 (only PF00330), jg23001 (2x PF00330)

Trimal -automated1 function; short or spurious sequences manually removed afterwards;
IQtree2 ML phylogeny best-fit model according to BIC: WAG+G4

IDH-1 / IDH-2

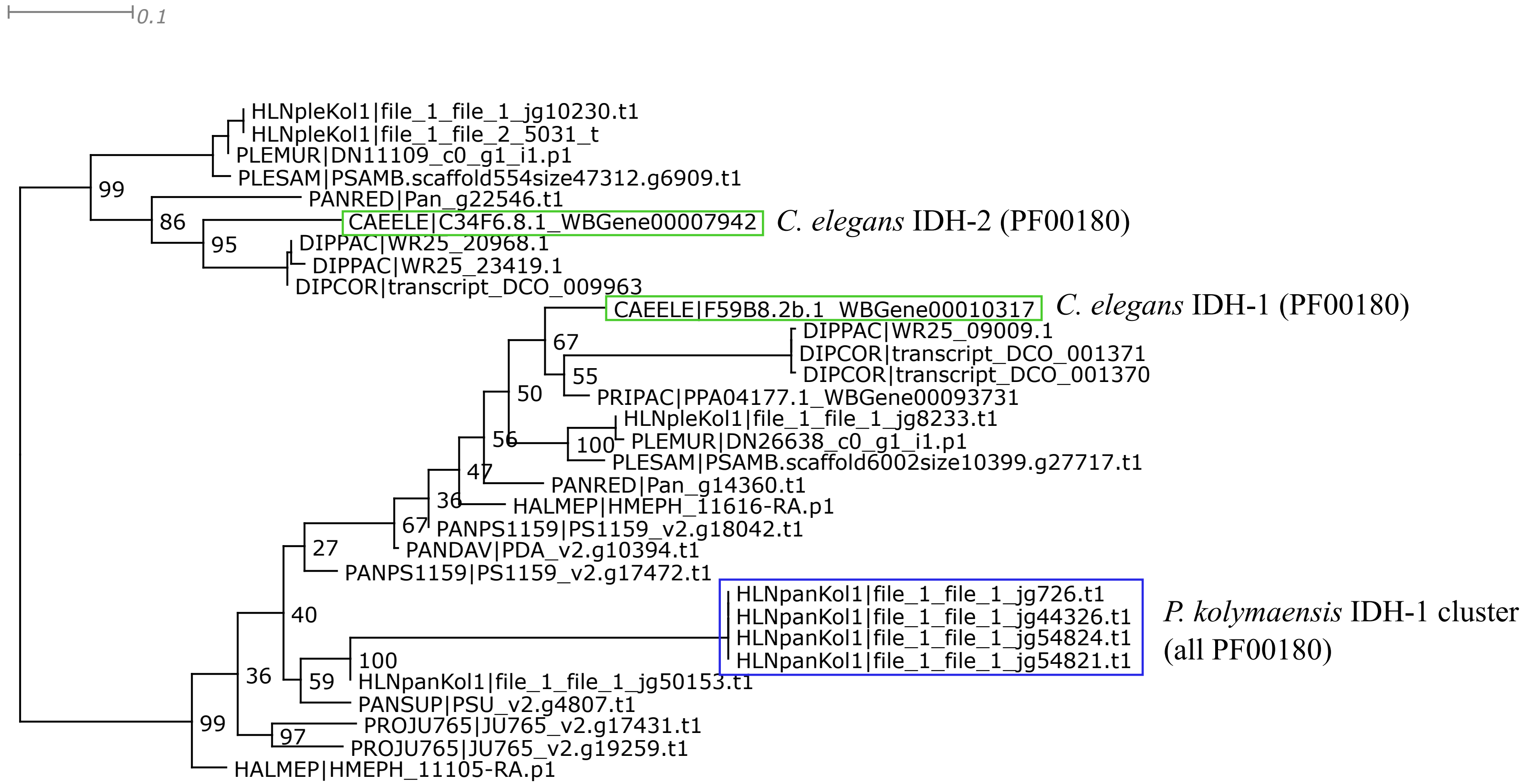

Trimal -automated1 function; short or spurious sequences manually removed afterwards;
IQtree2 ML phylogeny best-fit model according to BIC: WAG+I+G4

IDHA-1

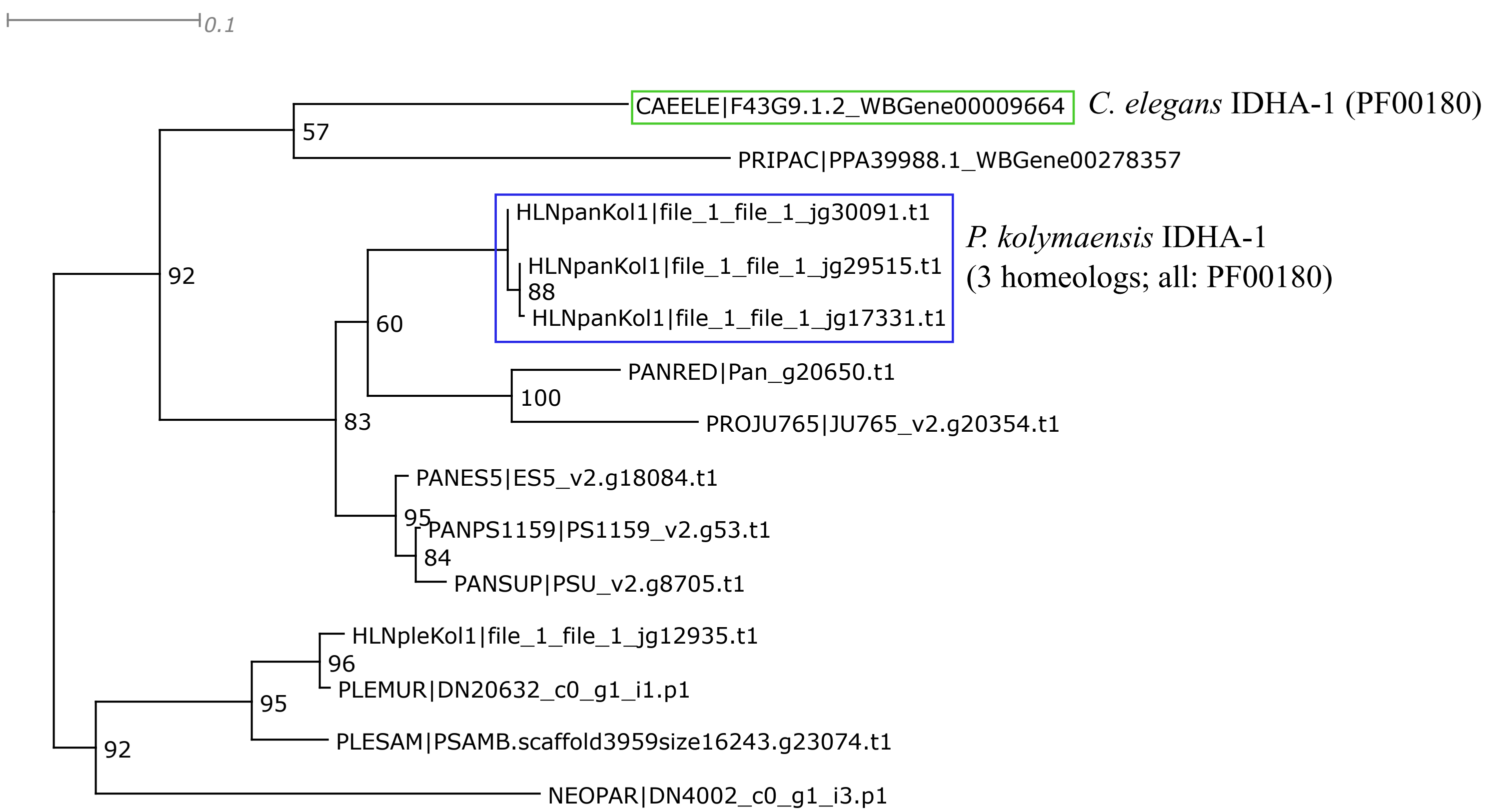

Trimal: 1. -resoverlap 0.75 -seqoverlap 80 functions; 2. -automated1 function; 3. short or spurious sequences manually removed afterwards; IQtree2 ML phylogeny best-fit model according to BIC: LG+I+G4

IDHB-1

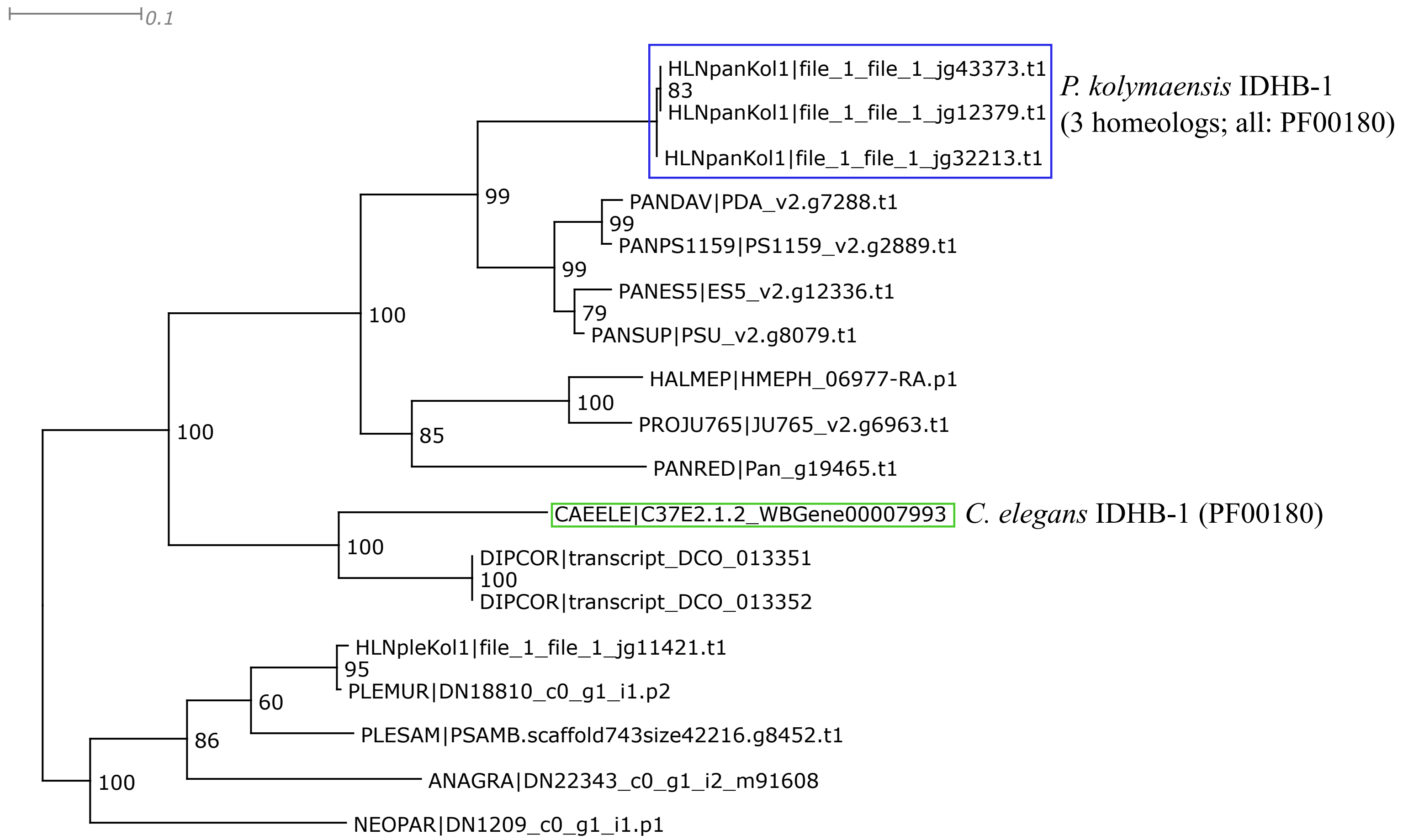

Trimal: 1. -resoverlap 0.75 -seqoverlap 80 functions; 2. -automated1 function; 3. short or spurious sequences manually removed afterwards; IQtree2 ML phylogeny best-fit model according to BIC: LG+I+G4

IDHG-1 / IDHG-2

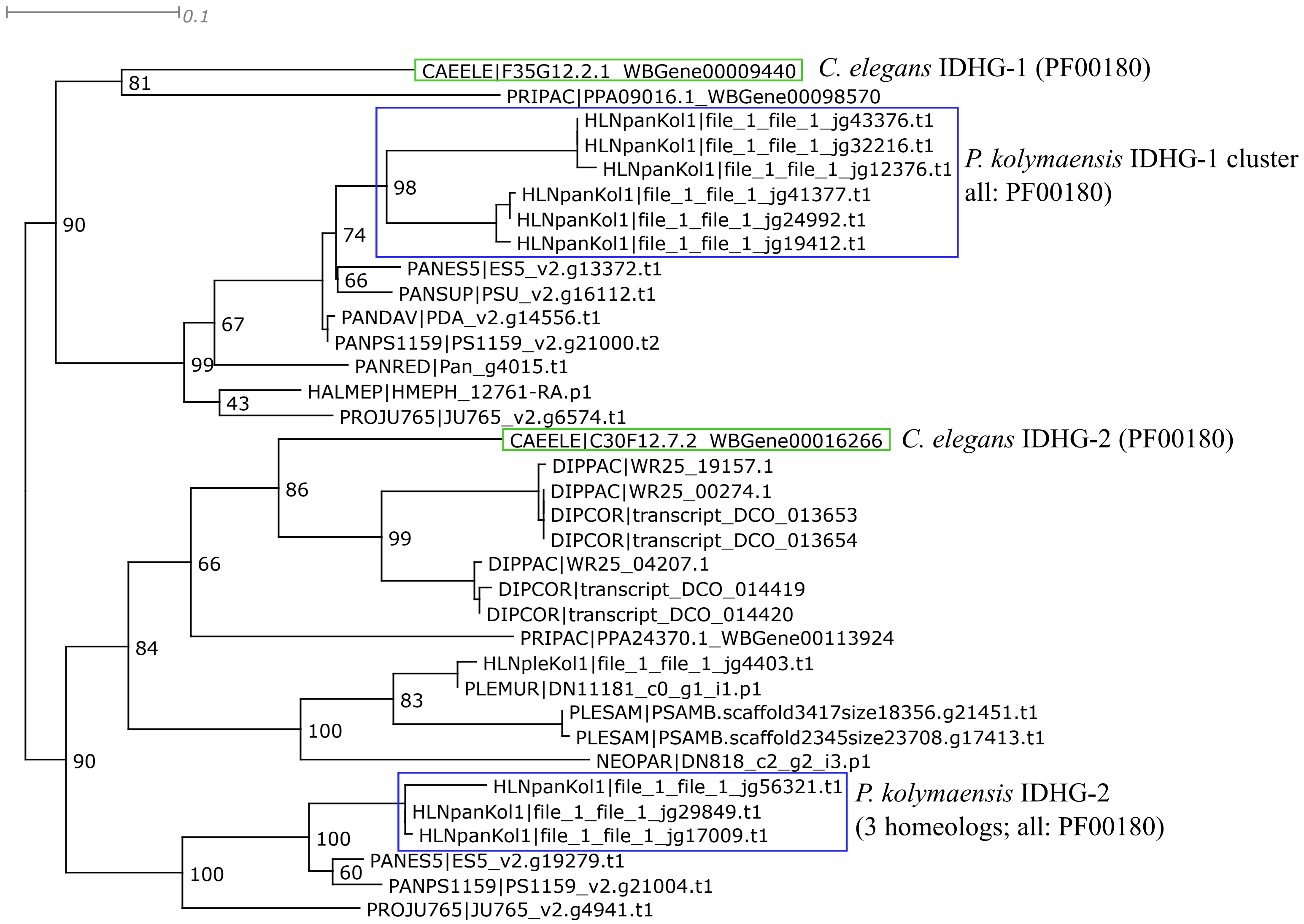

Trimal -automated1 function; short or spurious sequences manually removed afterwards;
IQtree2 ML phylogeny best-fit model according to BIC: LG+G4

MDH-1

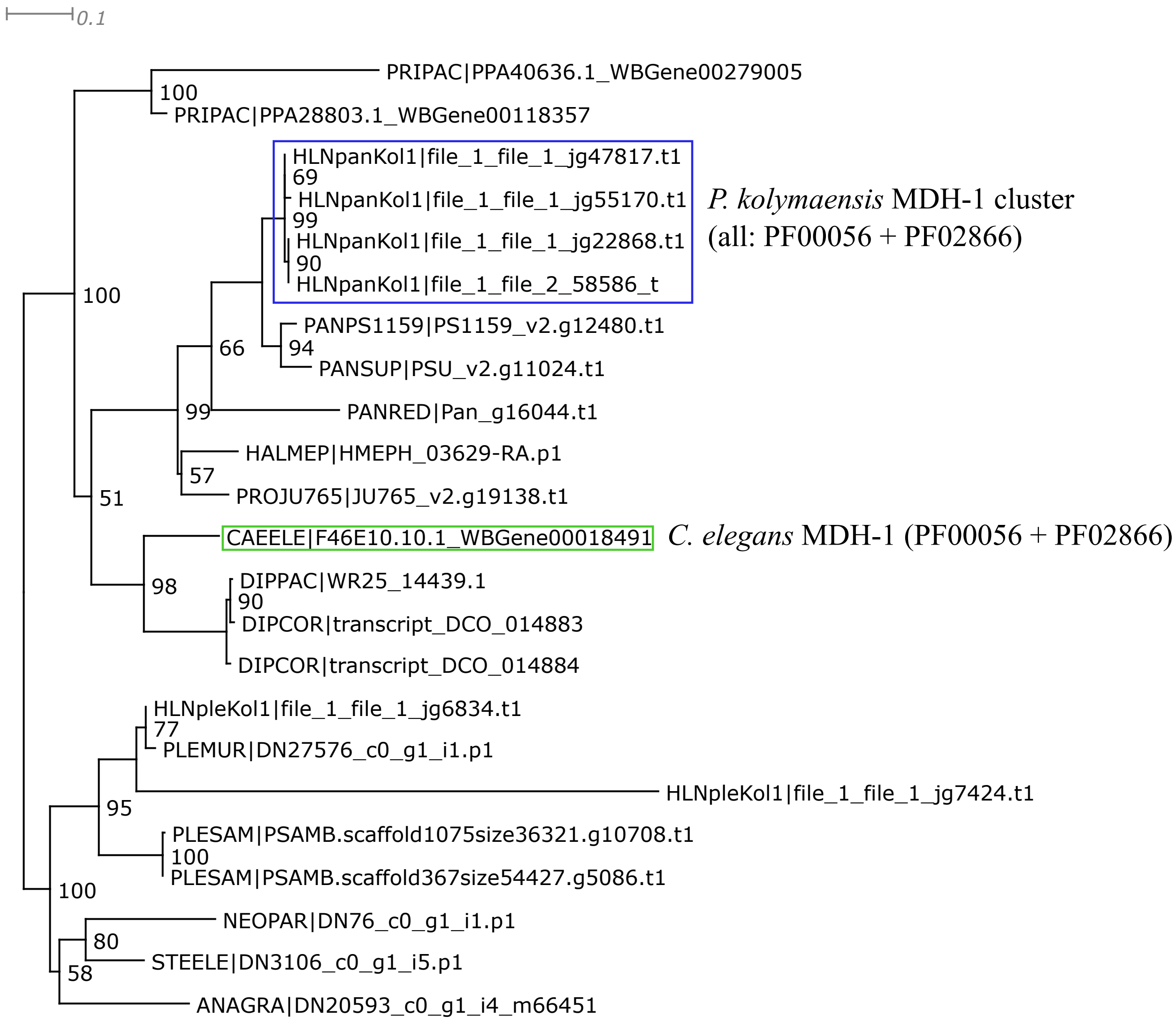

Trimal -automated1 function; short or spurious sequences manually removed afterwards;
IQtree2 ML phylogeny best-fit model according to BIC: LG+G4

MDH-2

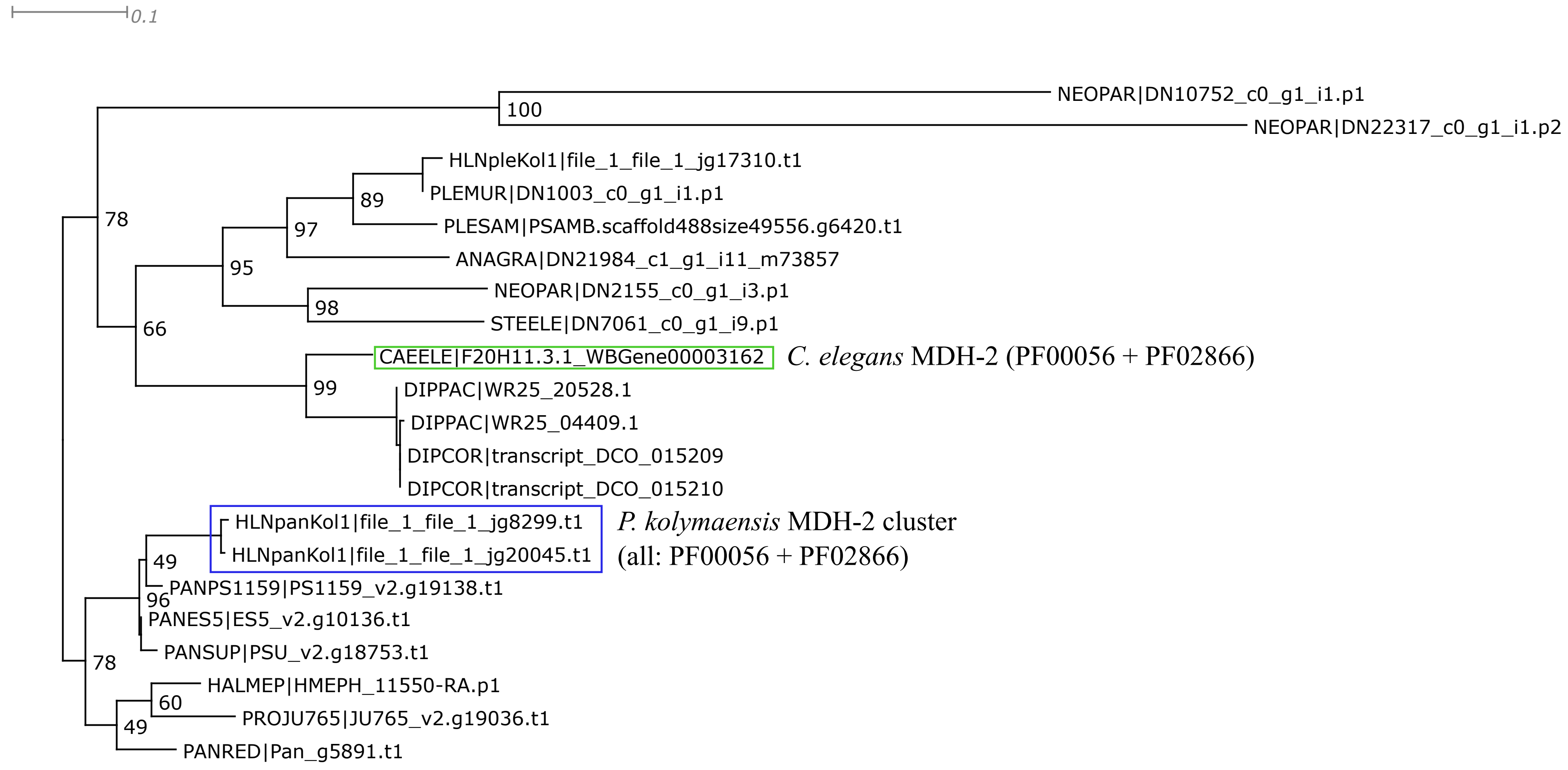

Trimal -automated1 function; short or spurious sequences manually removed afterwards;
IQtree2 ML phylogeny best-fit model according to BIC: LG+G4

OGDH-1 / OGDH-2

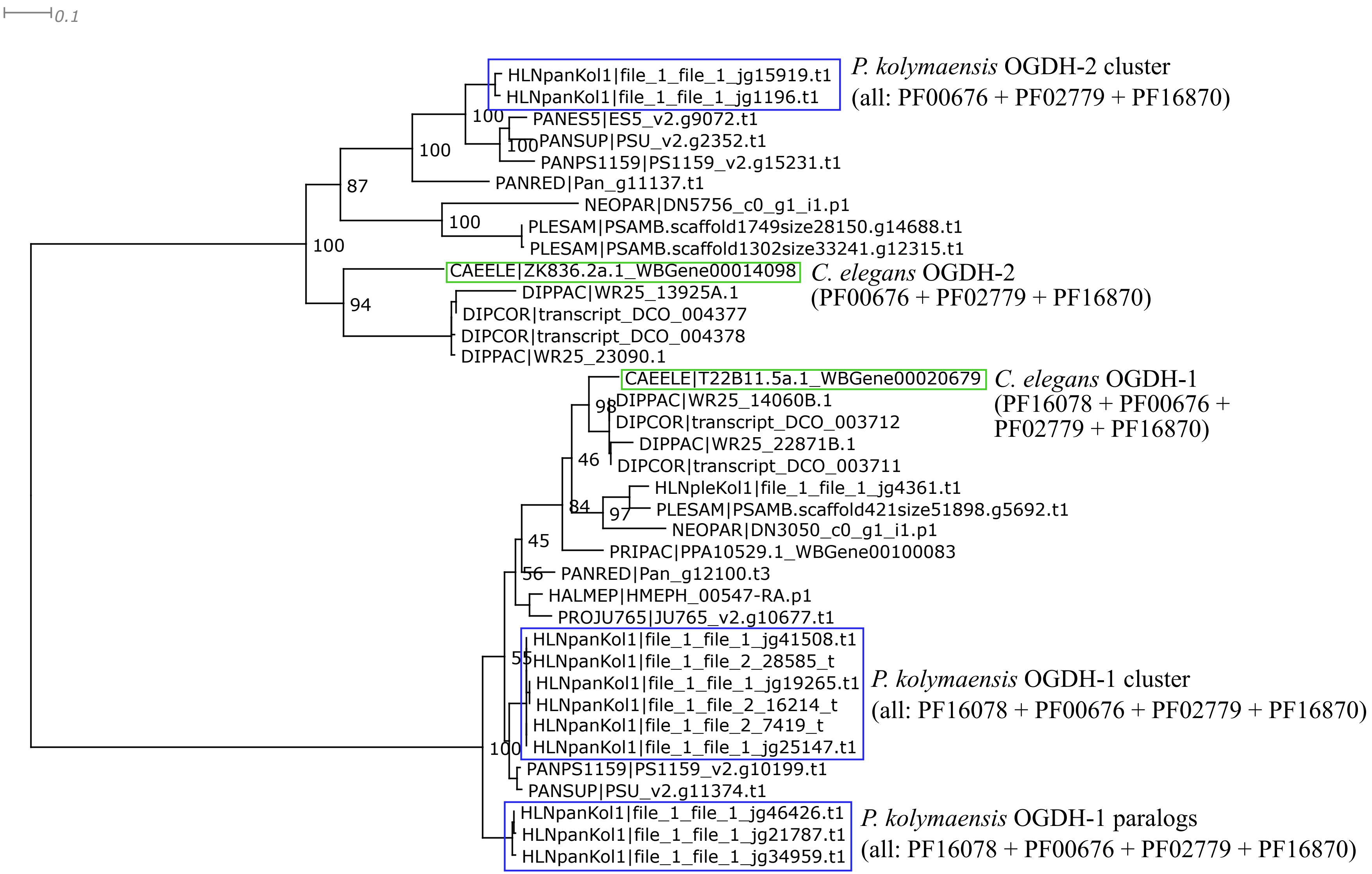

Trimal -automated1 function; short or spurious sequences manually removed afterwards;
IQtree2 ML phylogeny best-fit model according to BIC: LG+G4

SUCA-1

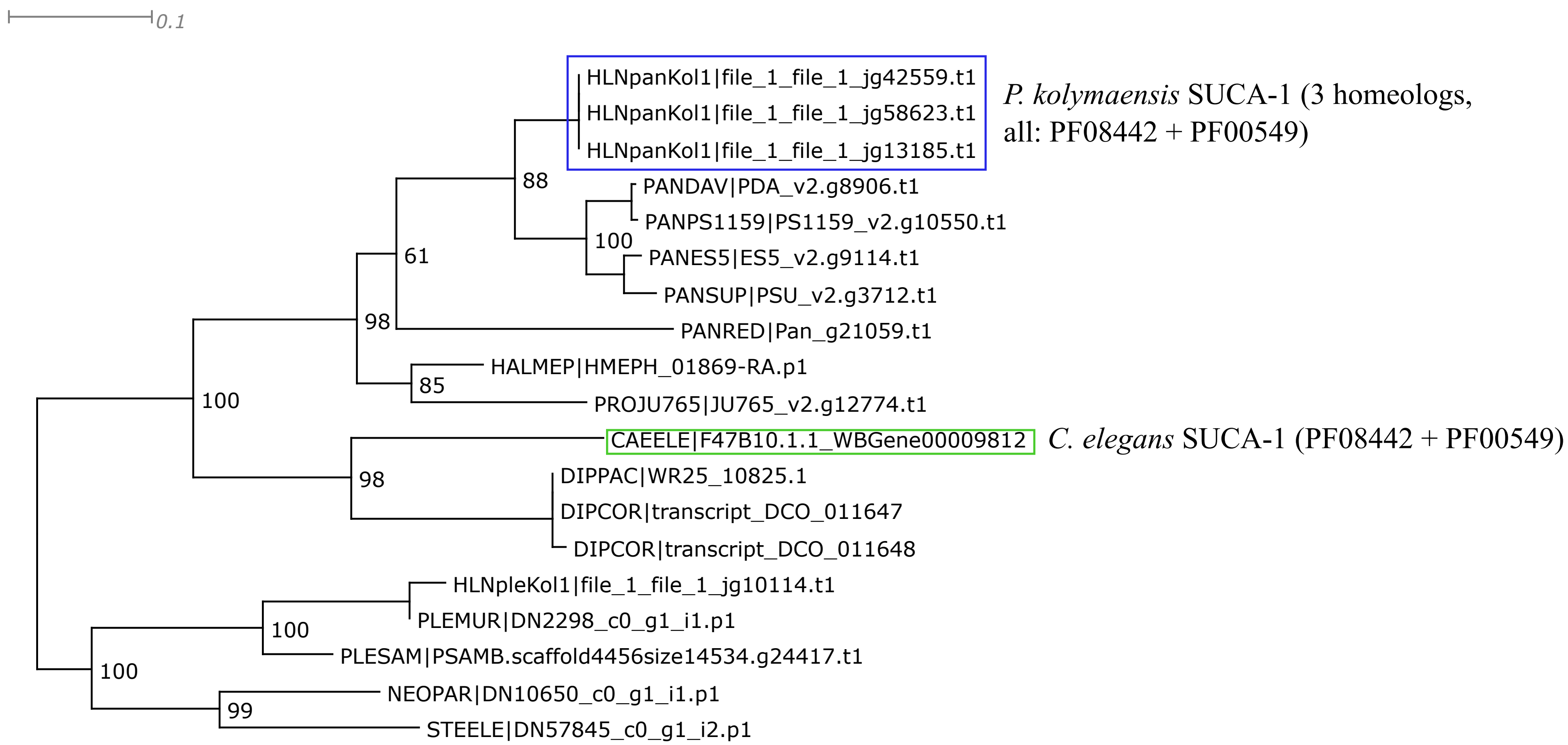

Trimal -automated1 function; short or spurious sequences manually removed afterwards;
IQtree2 ML phylogeny best-fit model according to BIC: LG+I+G4

SUCG-1

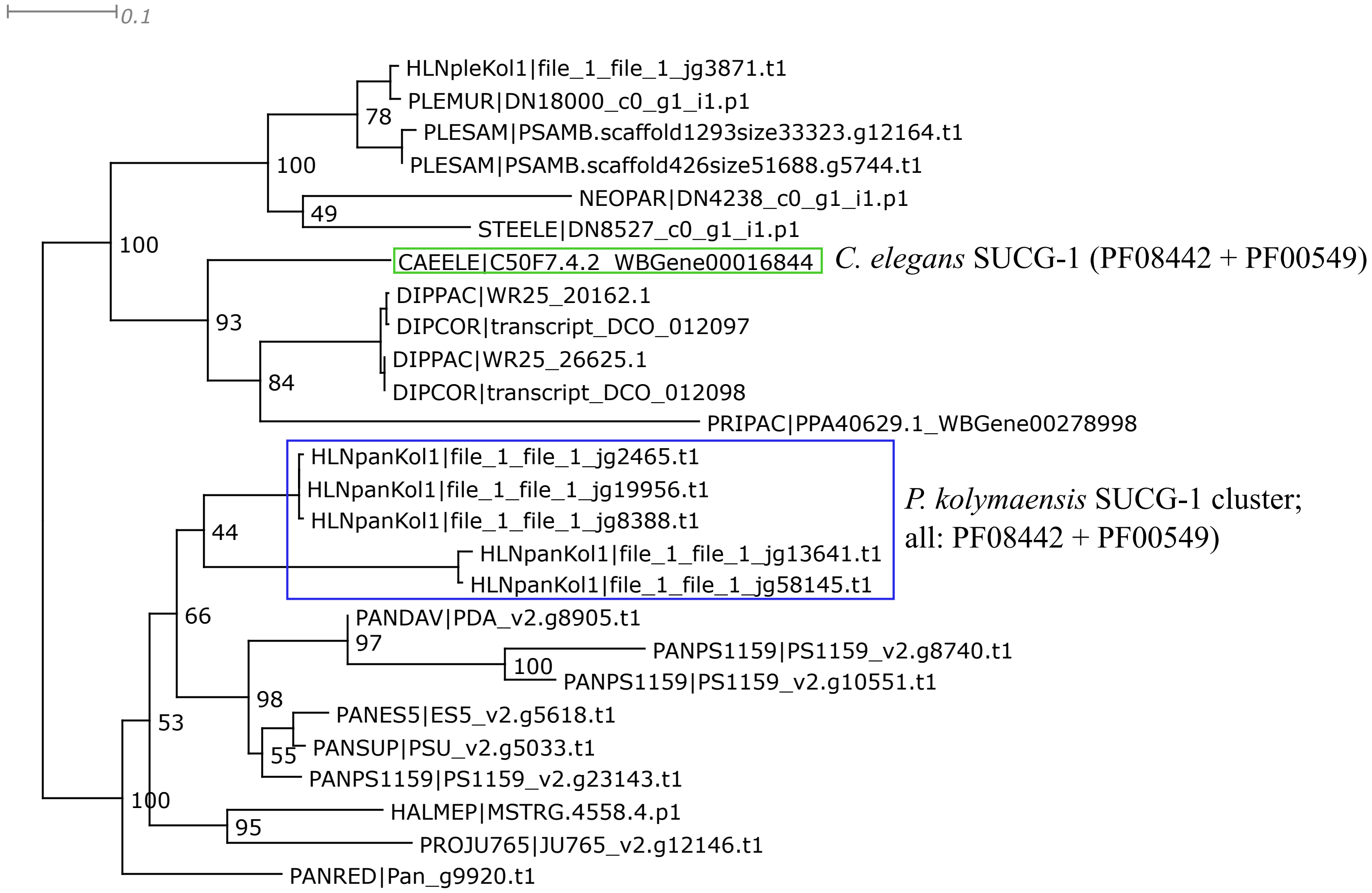

Trimal -automated1 function; short or spurious sequences manually removed afterwards;
IQtree2 ML phylogeny best-fit model according to BIC: LG+I+G4

SUCL-1 / SUCL-2

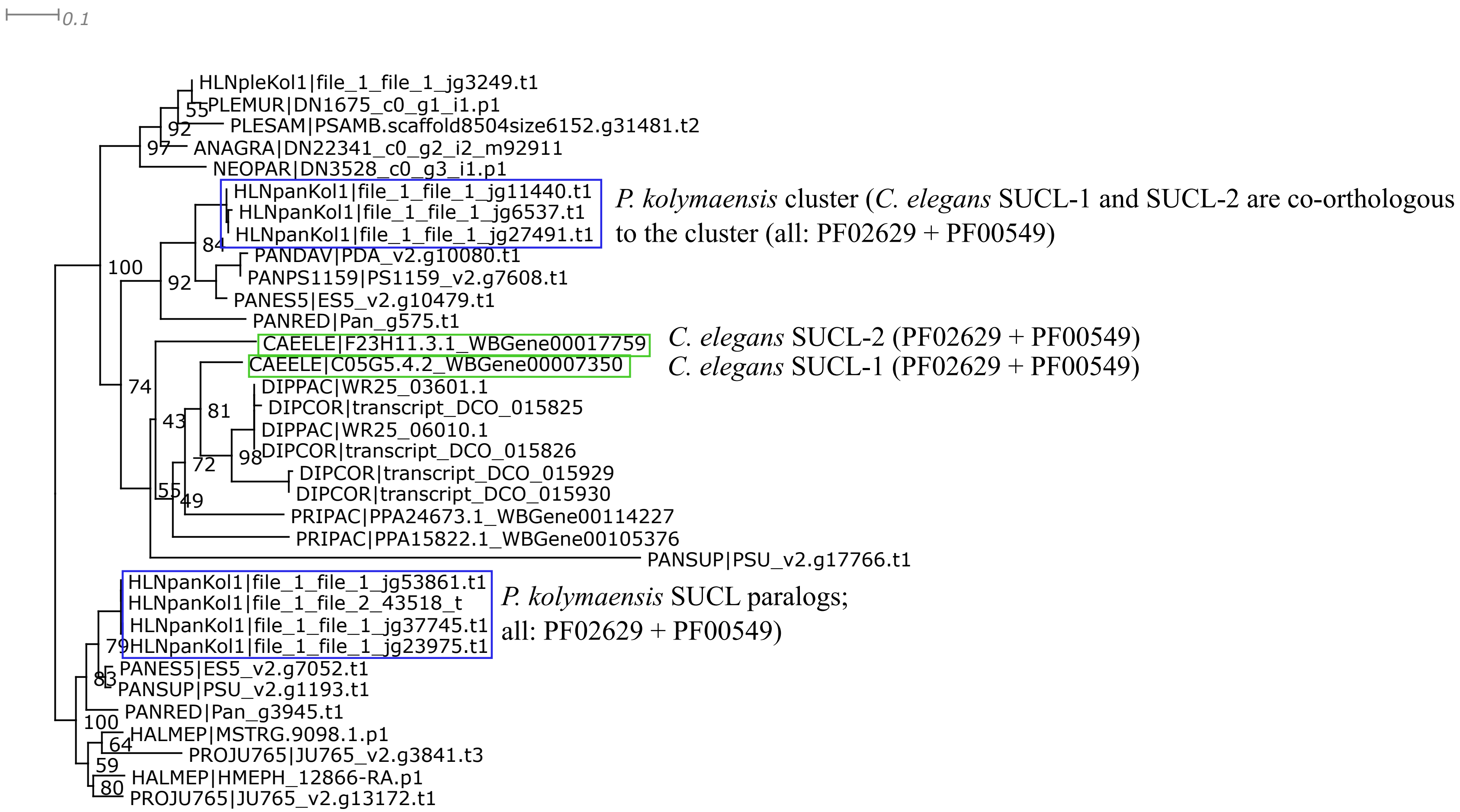

Trimal -automated1 function; short or spurious sequences manually removed afterwards;
IQtree2 ML phylogeny best-fit model according to BIC: LG+G4

SDHA-1 / SDHA-2

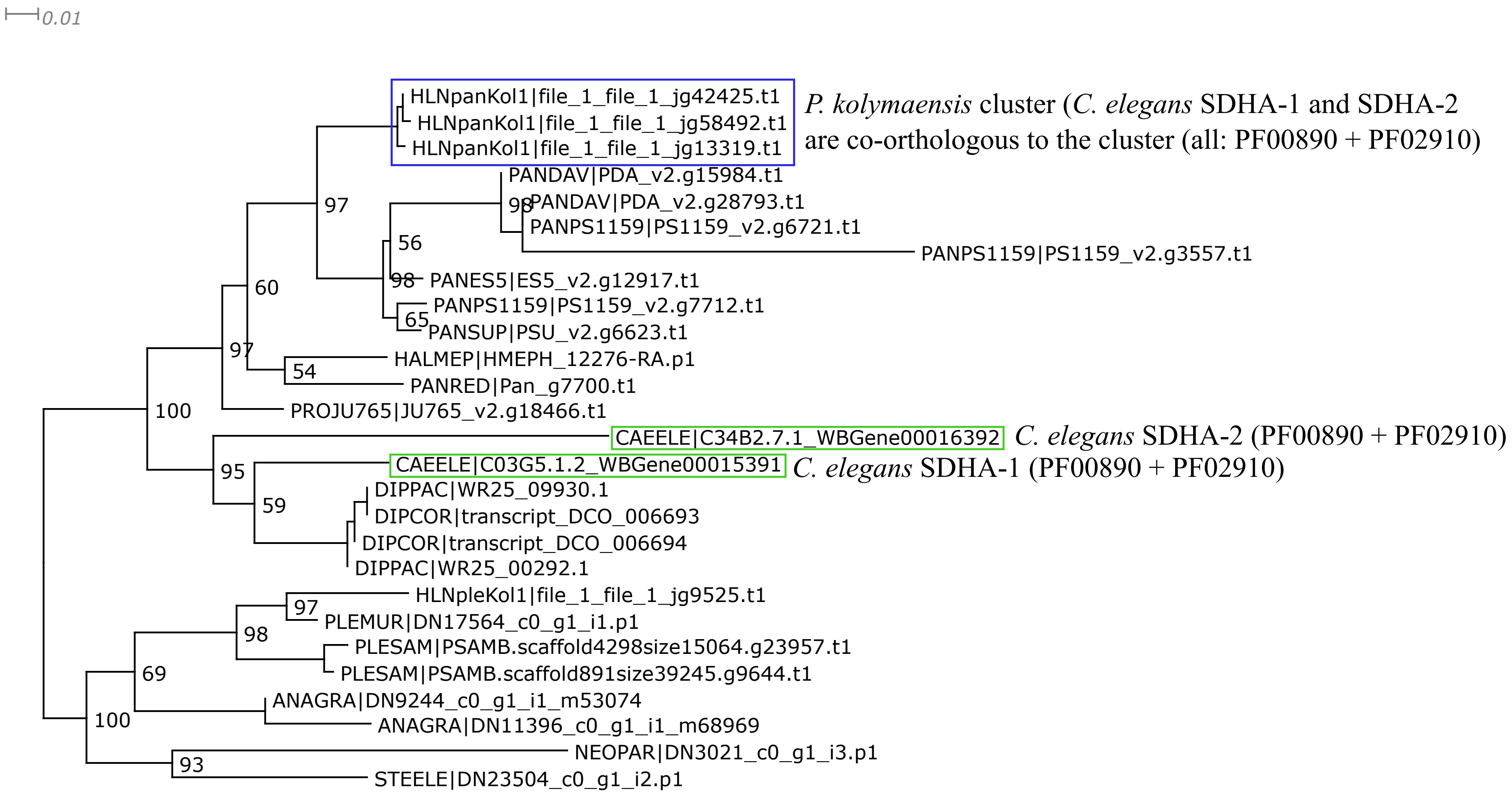

Trimal: 1. -resoverlap 0.75 -seqoverlap 75 functions; 2. -automated1 function; 3. short or spurious sequences manually removed afterwards; IQtree2 ML phylogeny best-fit model according to BIC: LG+I+G4

SDHB-1

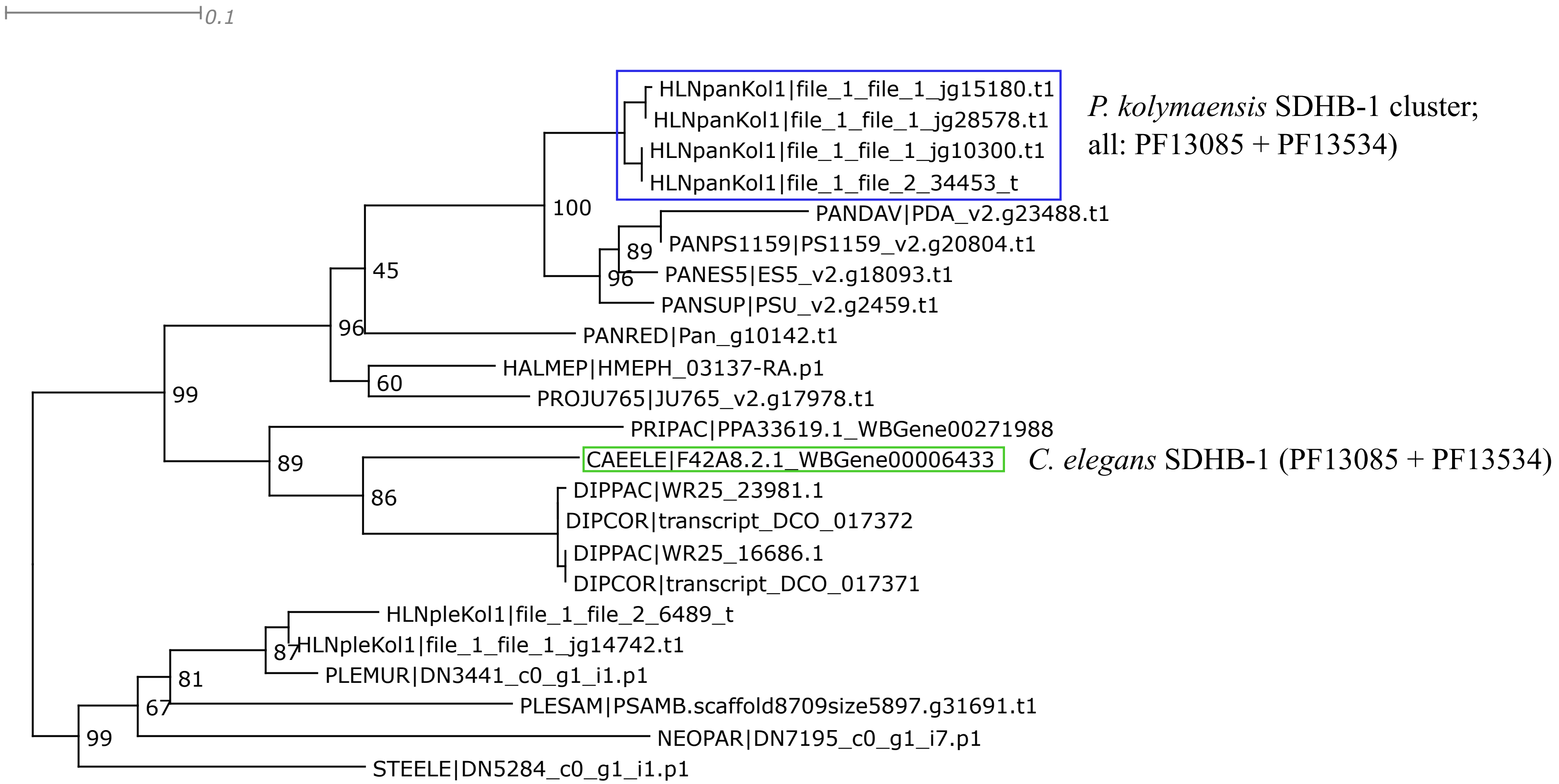

Trimal -automated1 function; short or spurious sequences manually removed afterwards;
IQtree2 ML phylogeny best-fit model according to BIC: WAG+I+G4

MEV-1

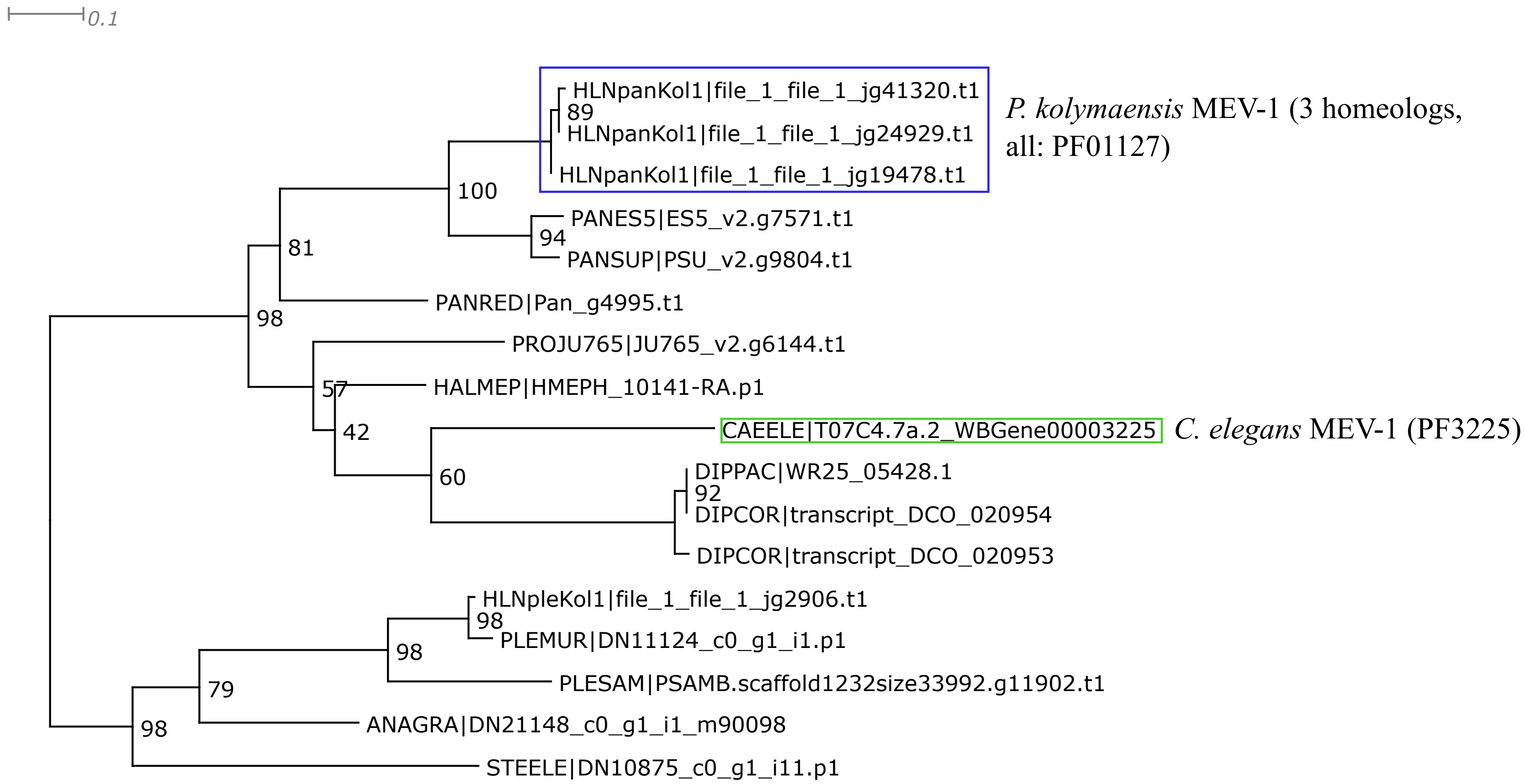

Trimal -automated1 function; short or spurious sequences manually removed afterwards;
IQtree2 ML phylogeny best-fit model according to BIC: LG+G4

FUM-1

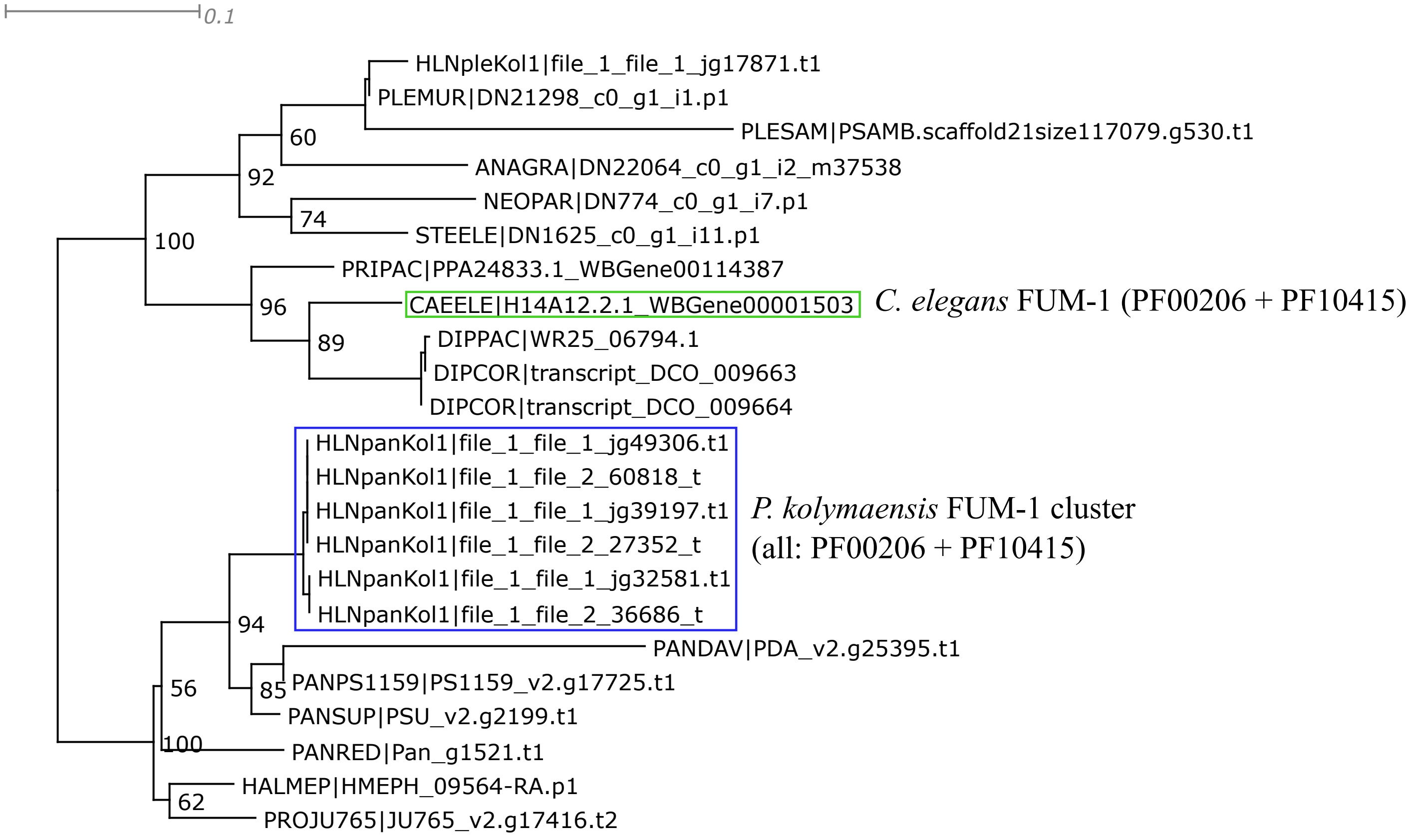

Trimal -automated1 function; short or spurious sequences manually removed afterwards;
IQtree2 ML phylogeny best-fit model according to BIC: WAG+G4

Glyoxylate shunt

ICL-1

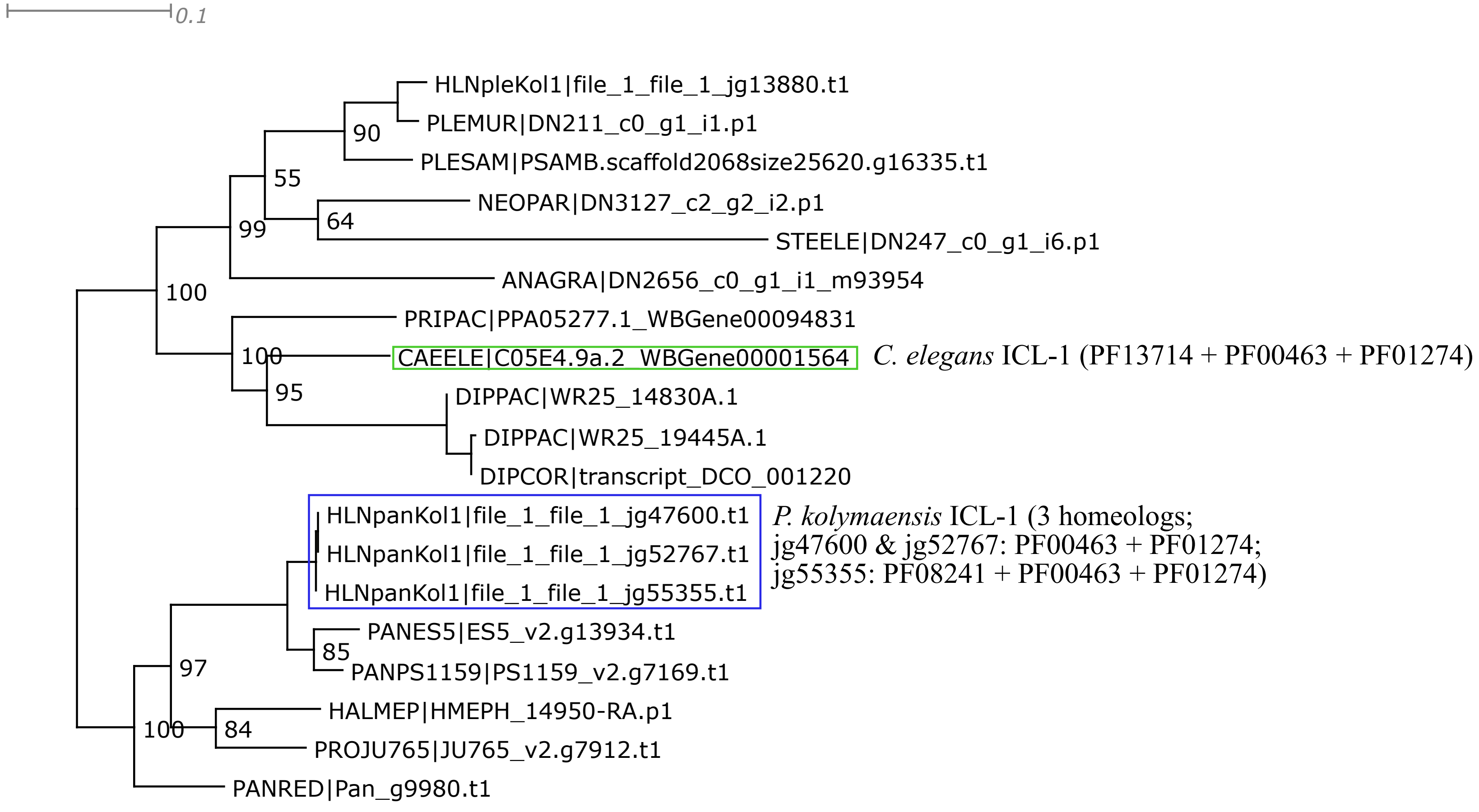

Trimal -automated1 function; short or spurious sequences manually removed afterwards;
IQtree2 ML phylogeny best-fit model according to BIC: LG+G4
Only sequences included that contain both Isocitrate lyase and Malate synthase combined.

Glycolysis / Gluconeogenesis

PDHB-1

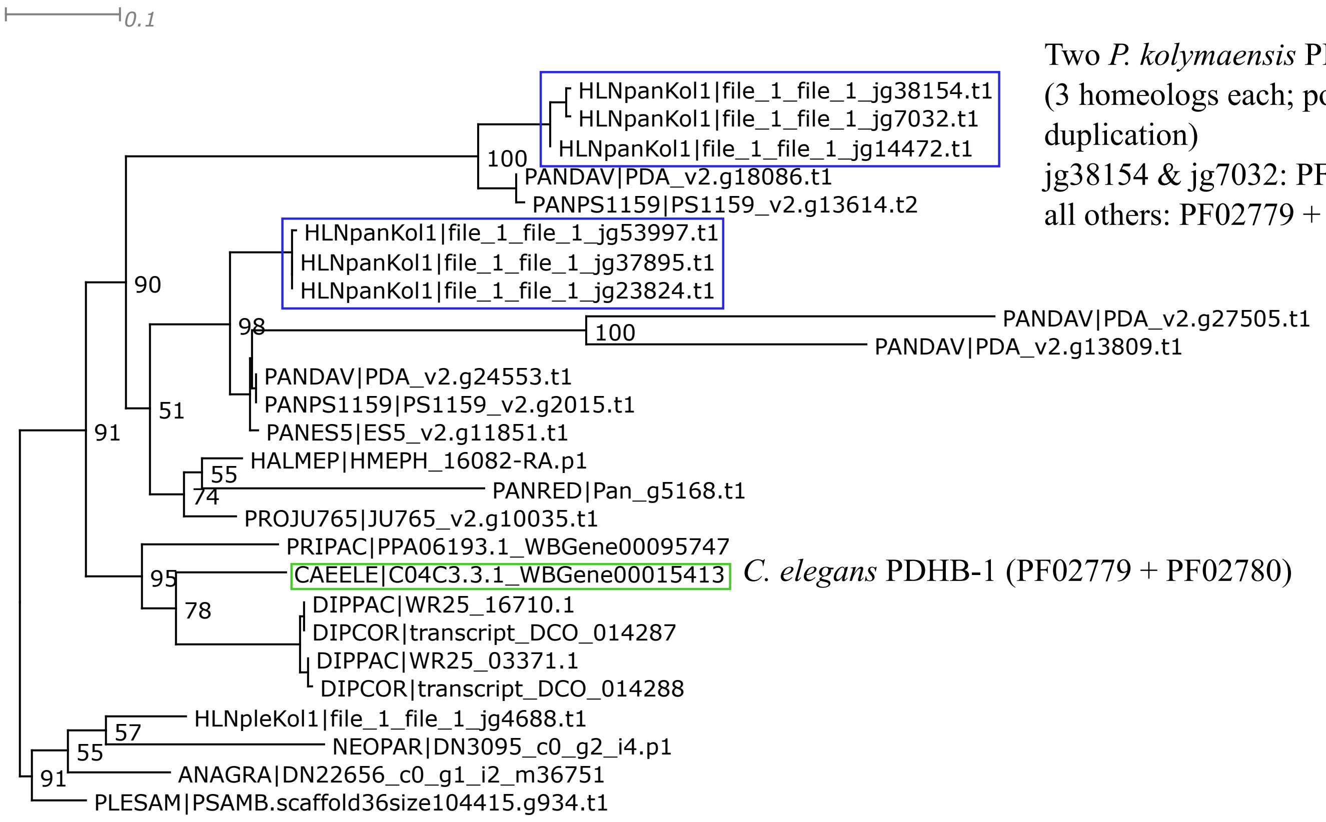

Two *P. kolymaensis* PDHB-1 clusters
(3 homeologs each; possibly *Panagrolaimus* wide duplication)
jg38154 & jg7032: PF02779 + PF02780 + PF00676
all others: PF02779 + PF02780

C. elegans PDHB-1 (PF02779 + PF02780)

Trimal -automated1 function; short or spurious sequences manually removed afterwards;
IQtree2 ML phylogeny best-fit model according to BIC: LG+G4

PDHA-1

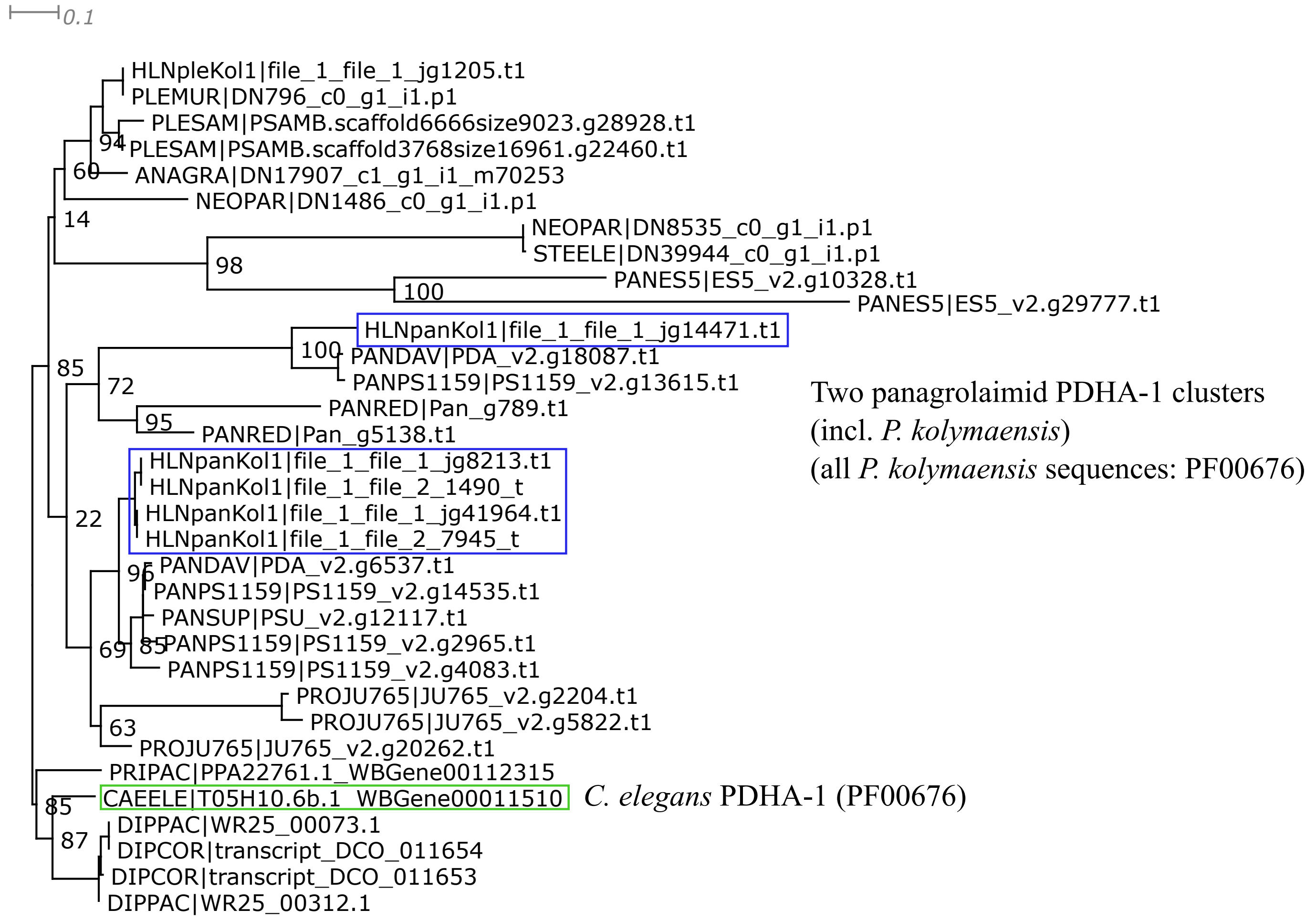

Trimal -automated1 function; short or spurious sequences manually removed afterwards;
IQtree2 ML phylogeny best-fit model according to BIC: LG+I+G4

DLAT-1 / DLAT-2

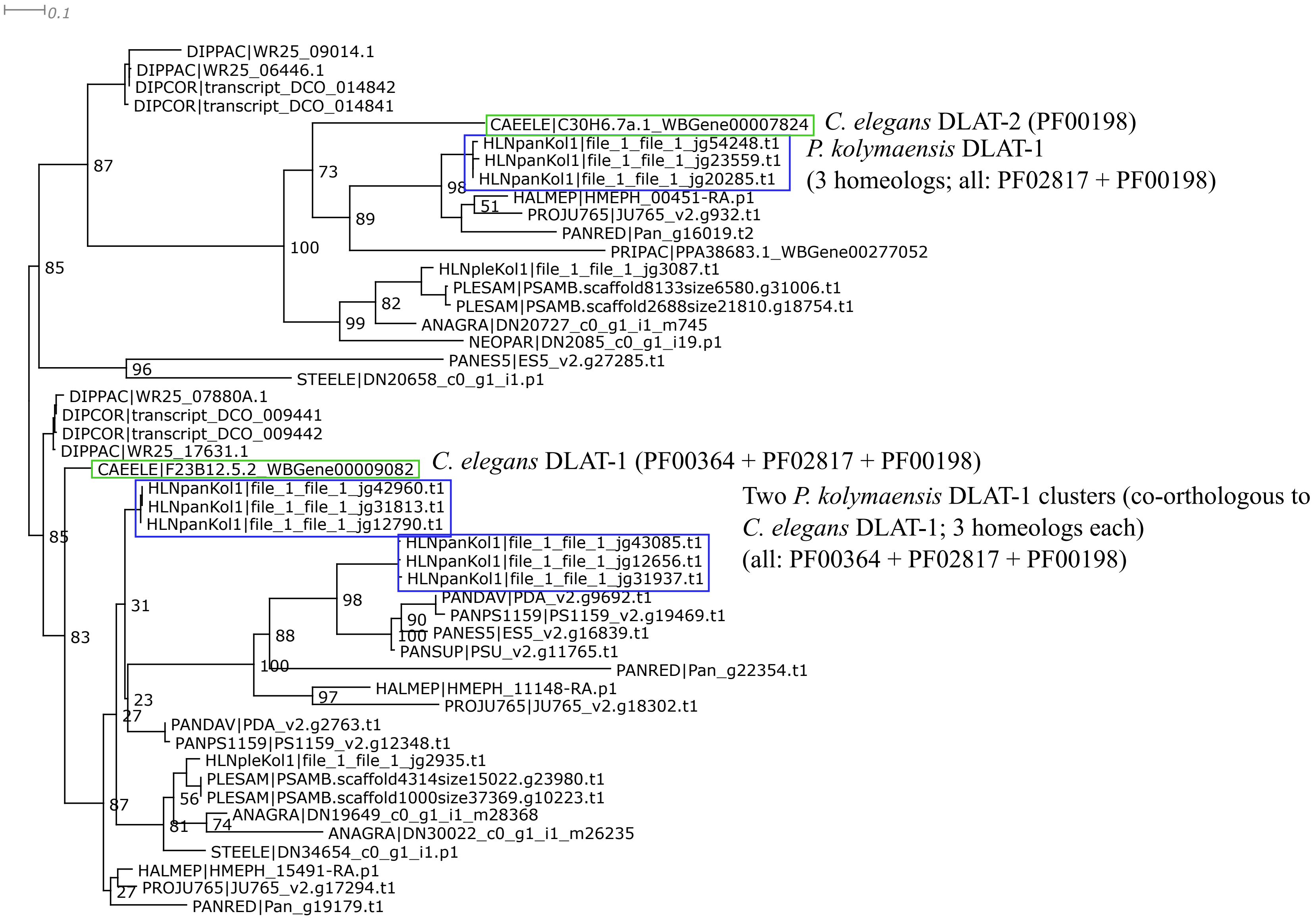

Trimal -automated1 function; short or spurious sequences manually removed afterwards;
IQtree2 ML phylogeny best-fit model according to BIC: LG+G4

DLD-1

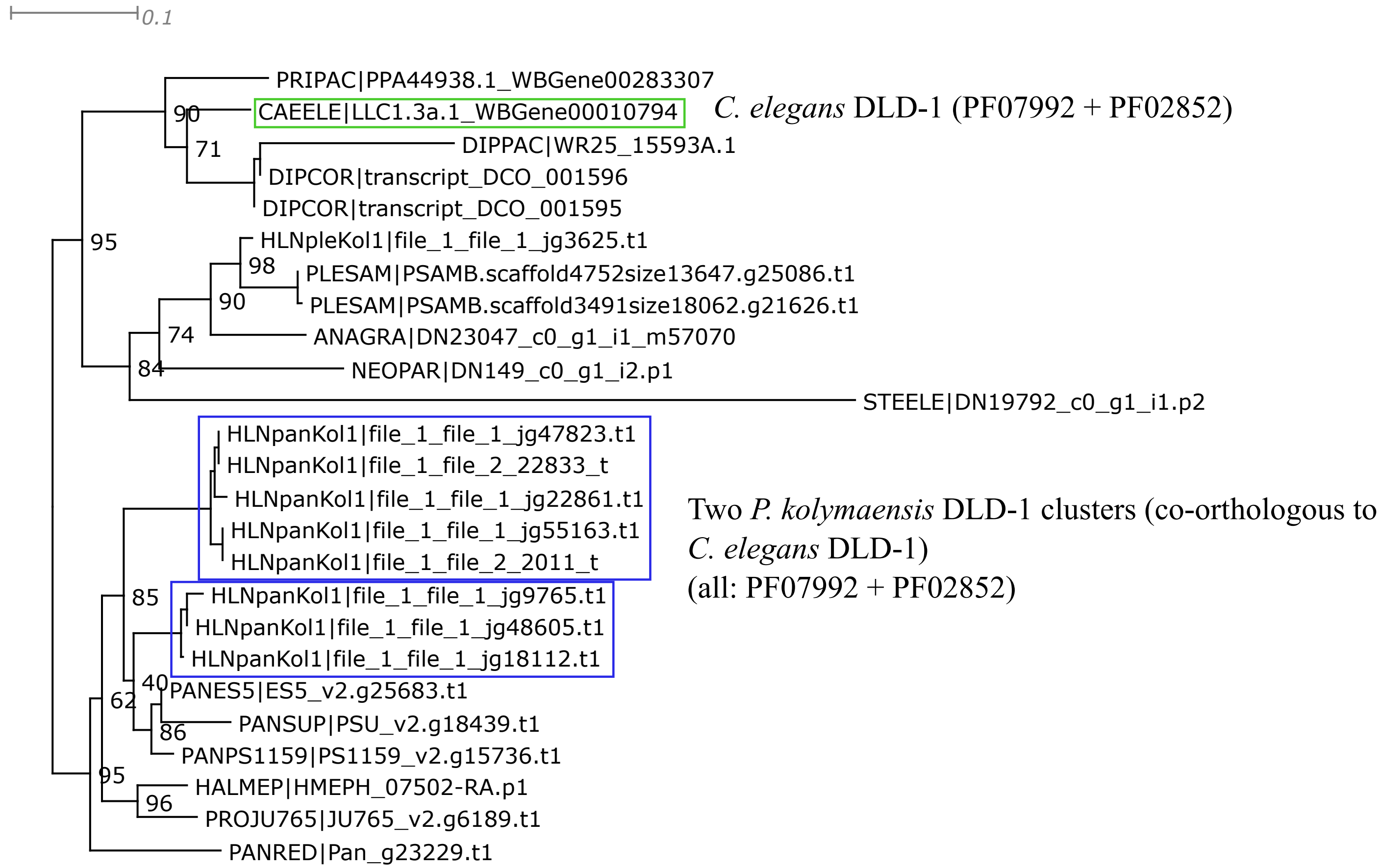

Trimal -automated1 function; short or spurious sequences manually removed afterwards;
IQtree2 ML phylogeny best-fit model according to BIC: LG+G4

PYC-1

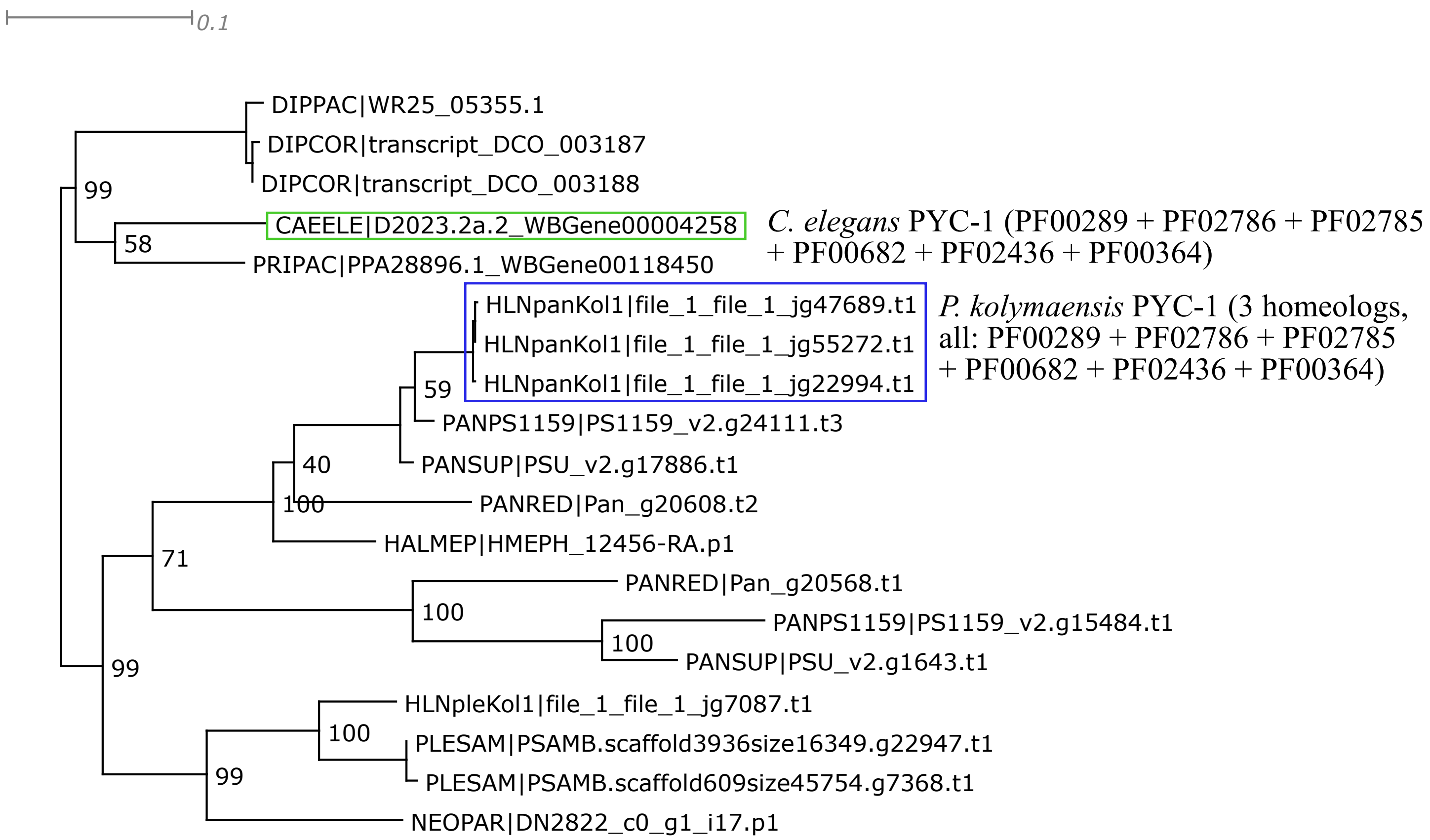

Trimal -automated1 function; short or spurious sequences manually removed afterwards;
IQtree2 ML phylogeny best-fit model according to BIC: LG+G4

PCK-1 / PCK-2

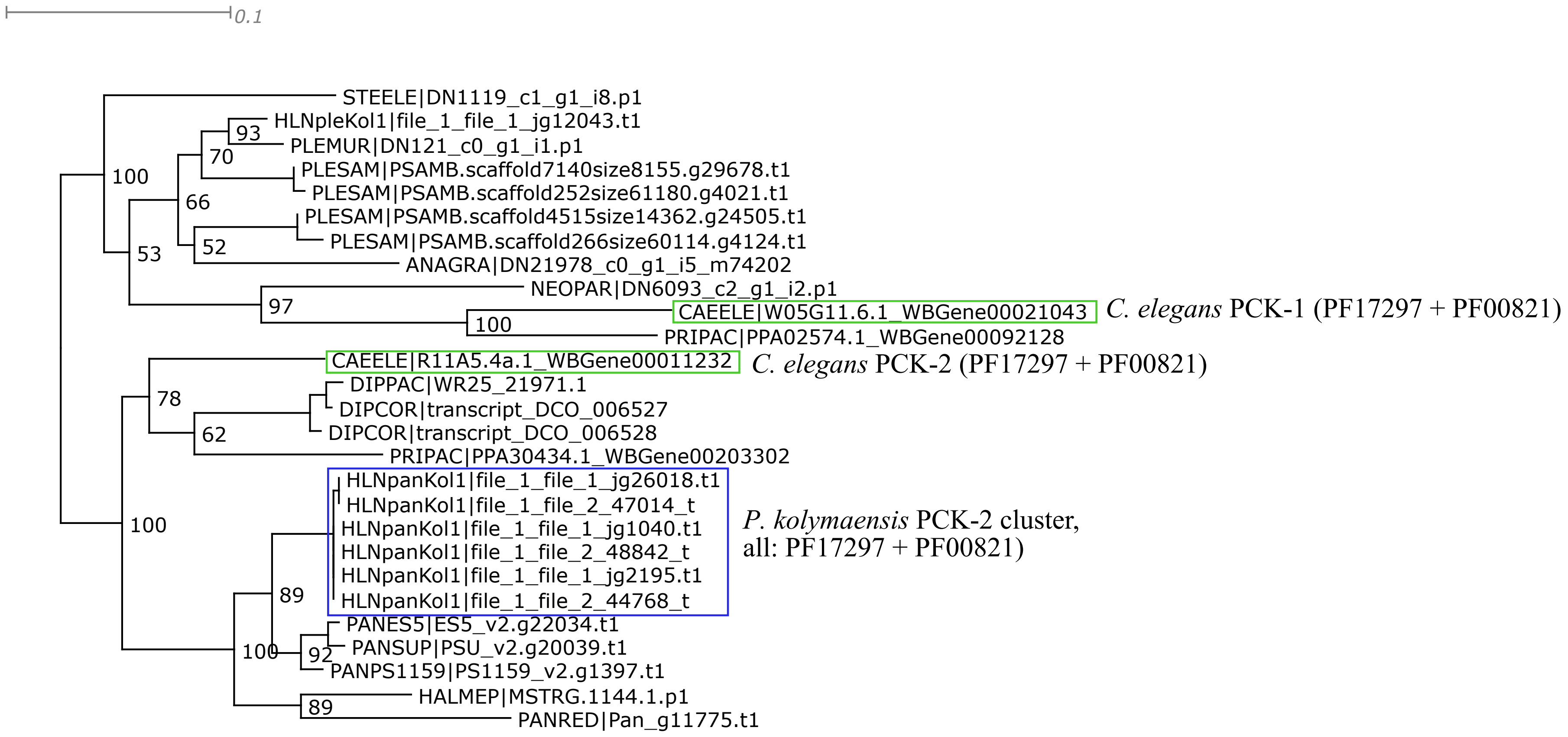

Trimal -automated1 function; short or spurious sequences manually removed afterwards;
IQtree2 ML phylogeny best-fit model according to BIC: LG+I+G4

PYK-1 / PYK-2

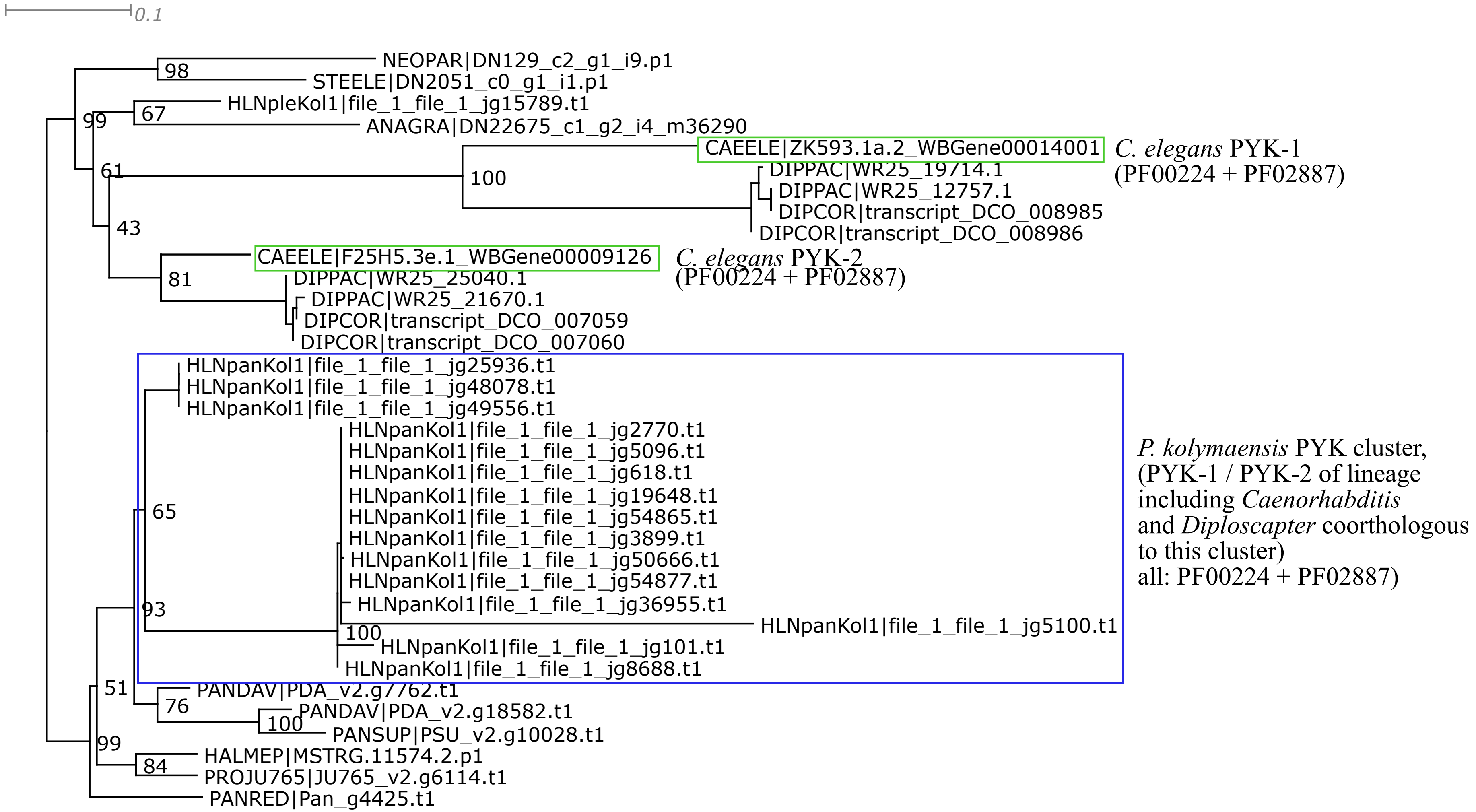

Trimal -automated1 function; short or spurious sequences manually removed afterwards;
IQtree2 ML phylogeny best-fit model according to BIC: LG+G4

ENOL-1

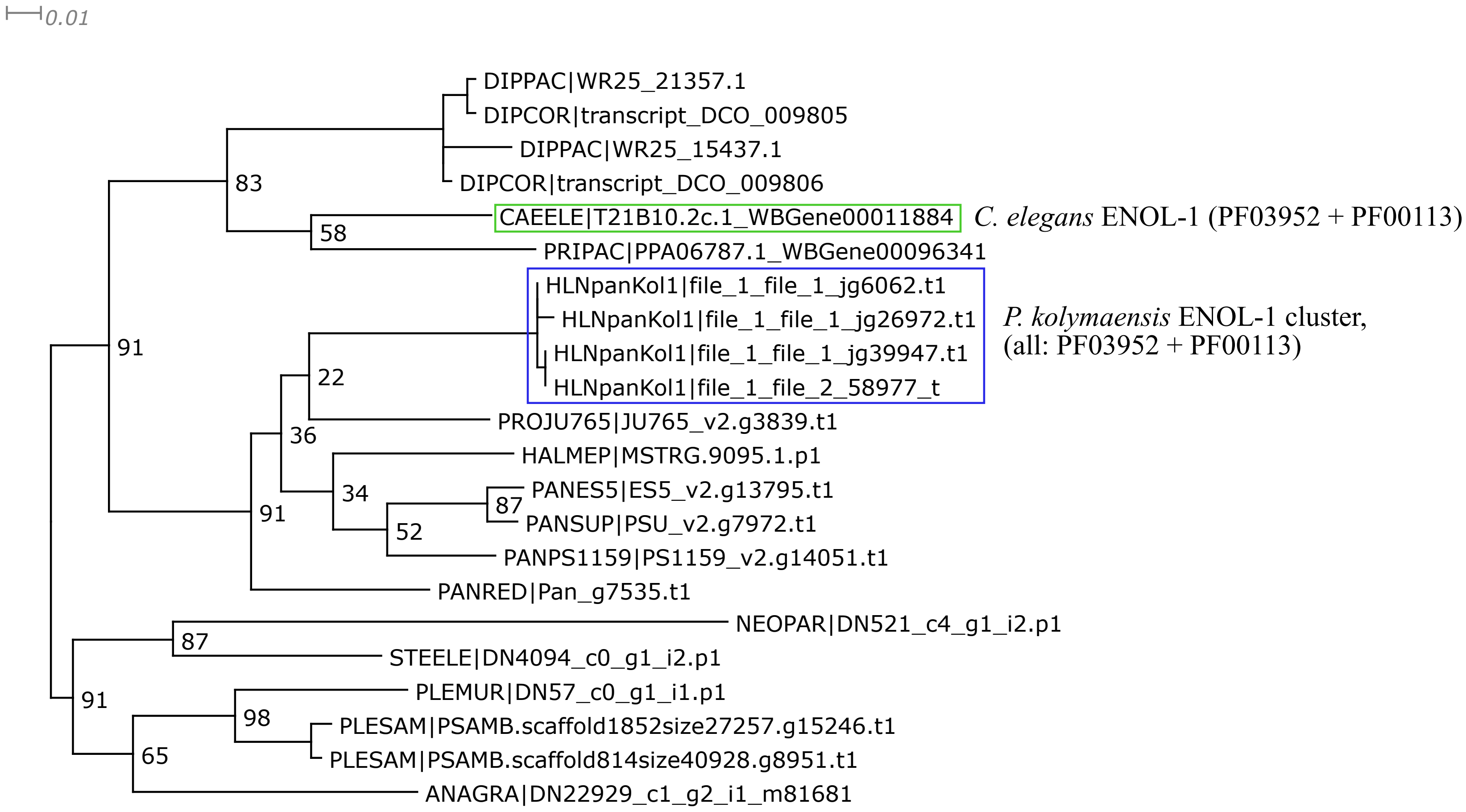

Trimal: 1. -resoverlap 0.75 -seqoverlap 80 functions; 2. -automated1 function; short or spurious sequences manually removed afterwards;
IQtree2 ML phylogeny best-fit model according to BIC: LG+G4

IPGM-1

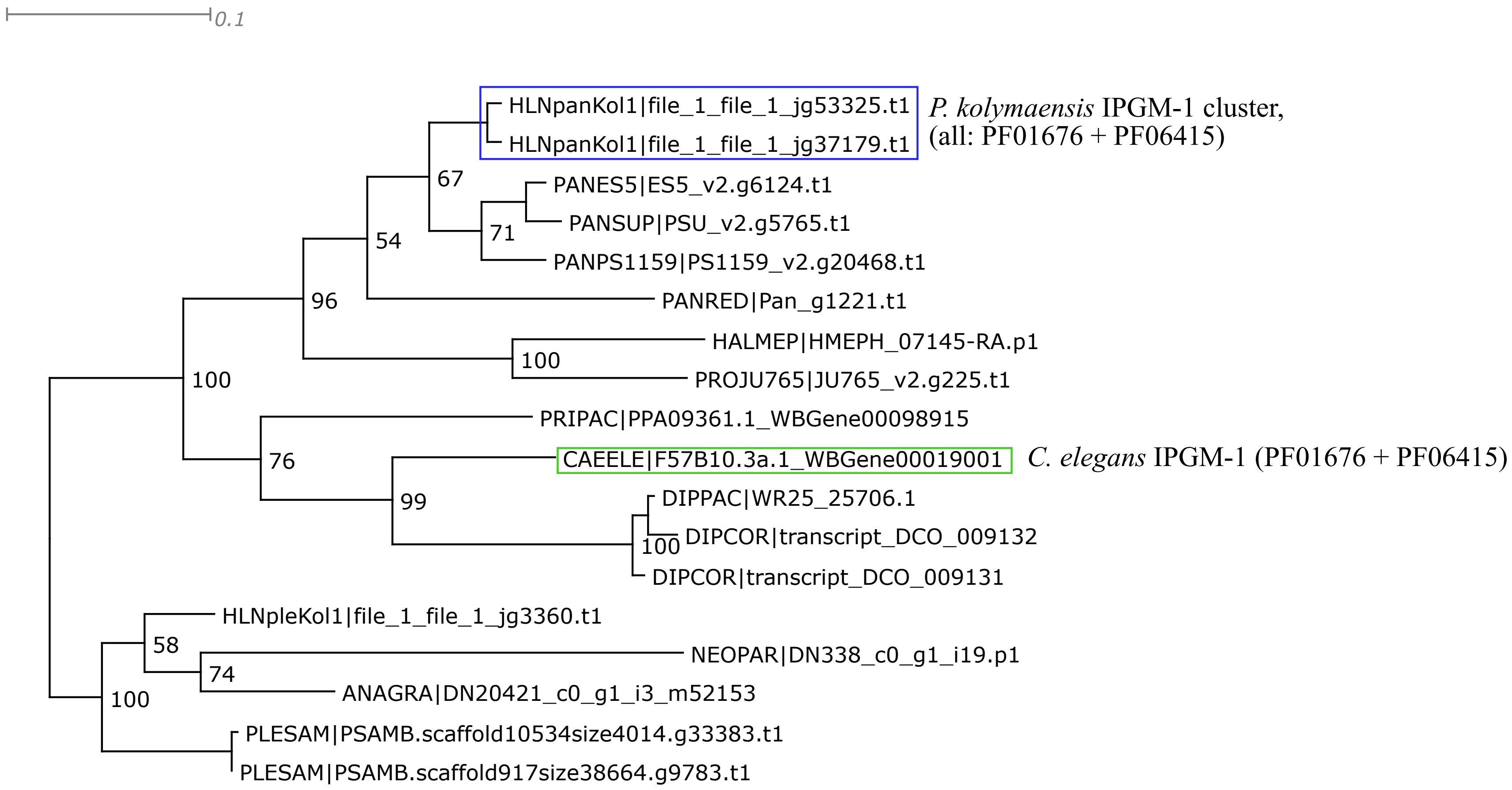

Trimal -automated1 function; short or spurious sequences manually removed afterwards;
IQtree2 ML phylogeny best-fit model according to BIC: LG+G4

PGK-1

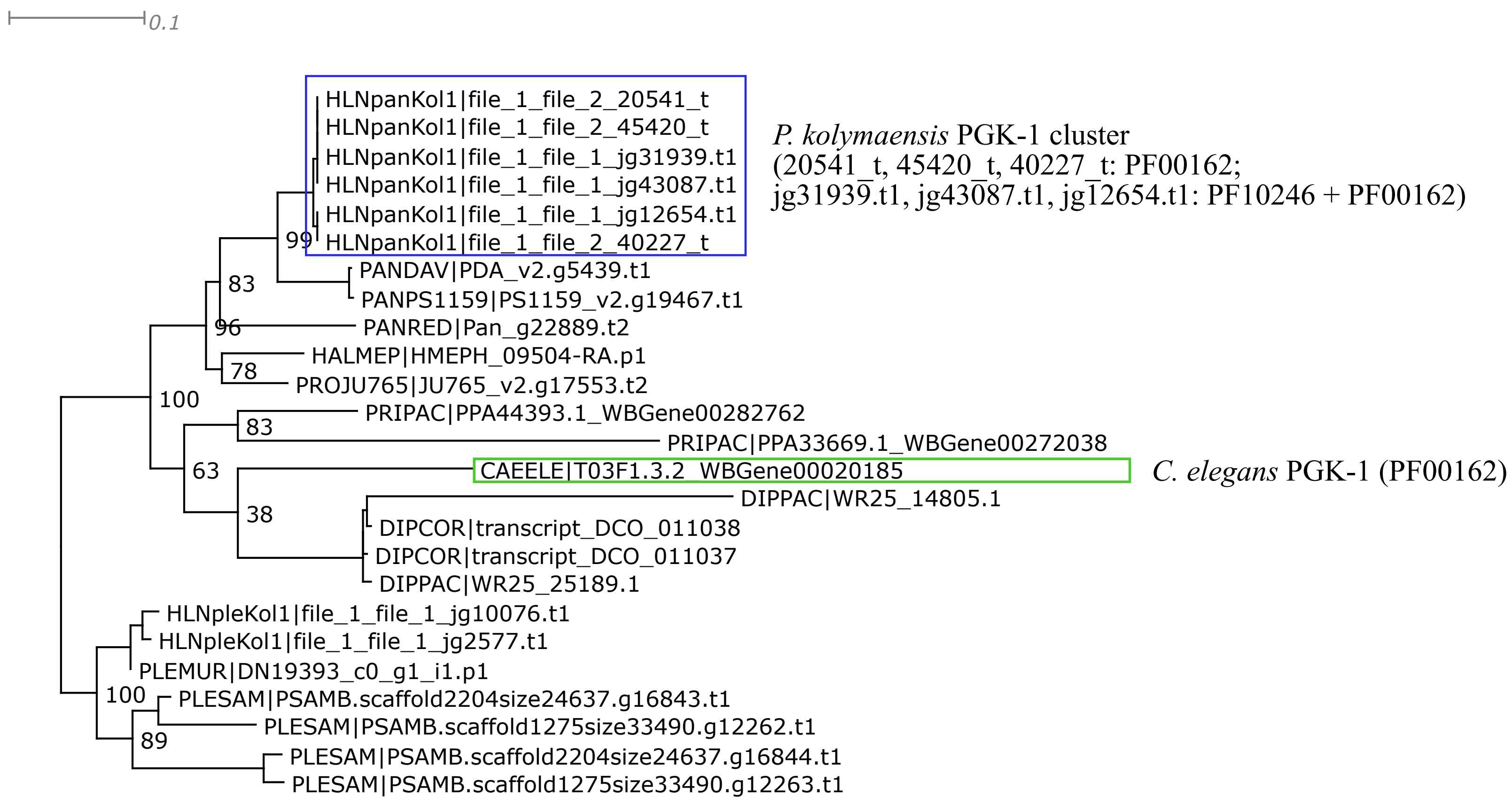

Trimal -automated1 function; short or spurious sequences manually removed afterwards;
IQtree2 ML phylogeny best-fit model according to BIC: WAG+G4

TPI-1

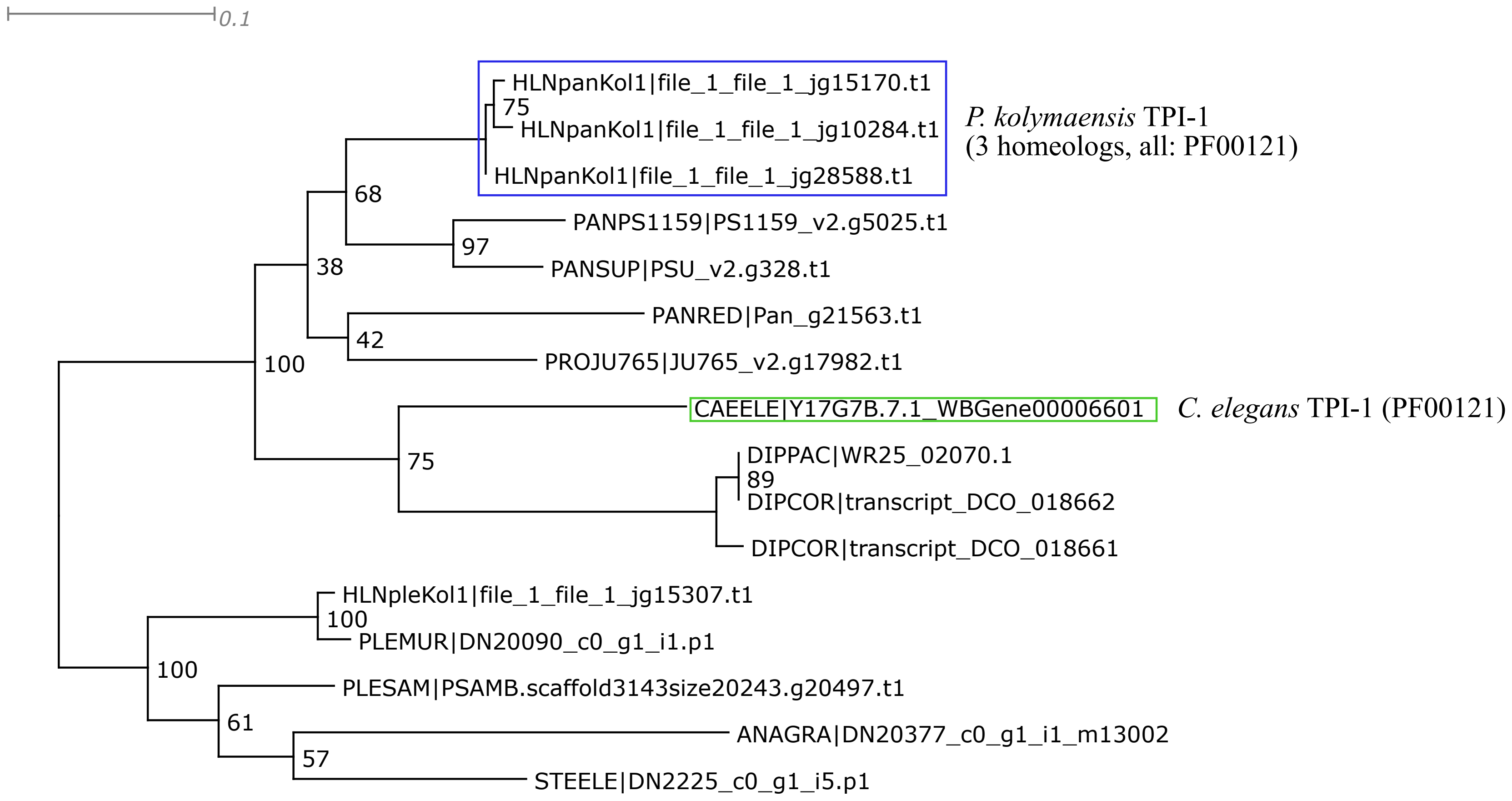

Trimal: 1. -resoverlap 0.75 -seqoverlap 80 functions; 2. -automated1 function; short or spurious sequences manually removed afterwards;

IQtree2 ML phylogeny best-fit model according to BIC: LG+G4

GPD-1, GPD-2, GPD-3, GPD-4

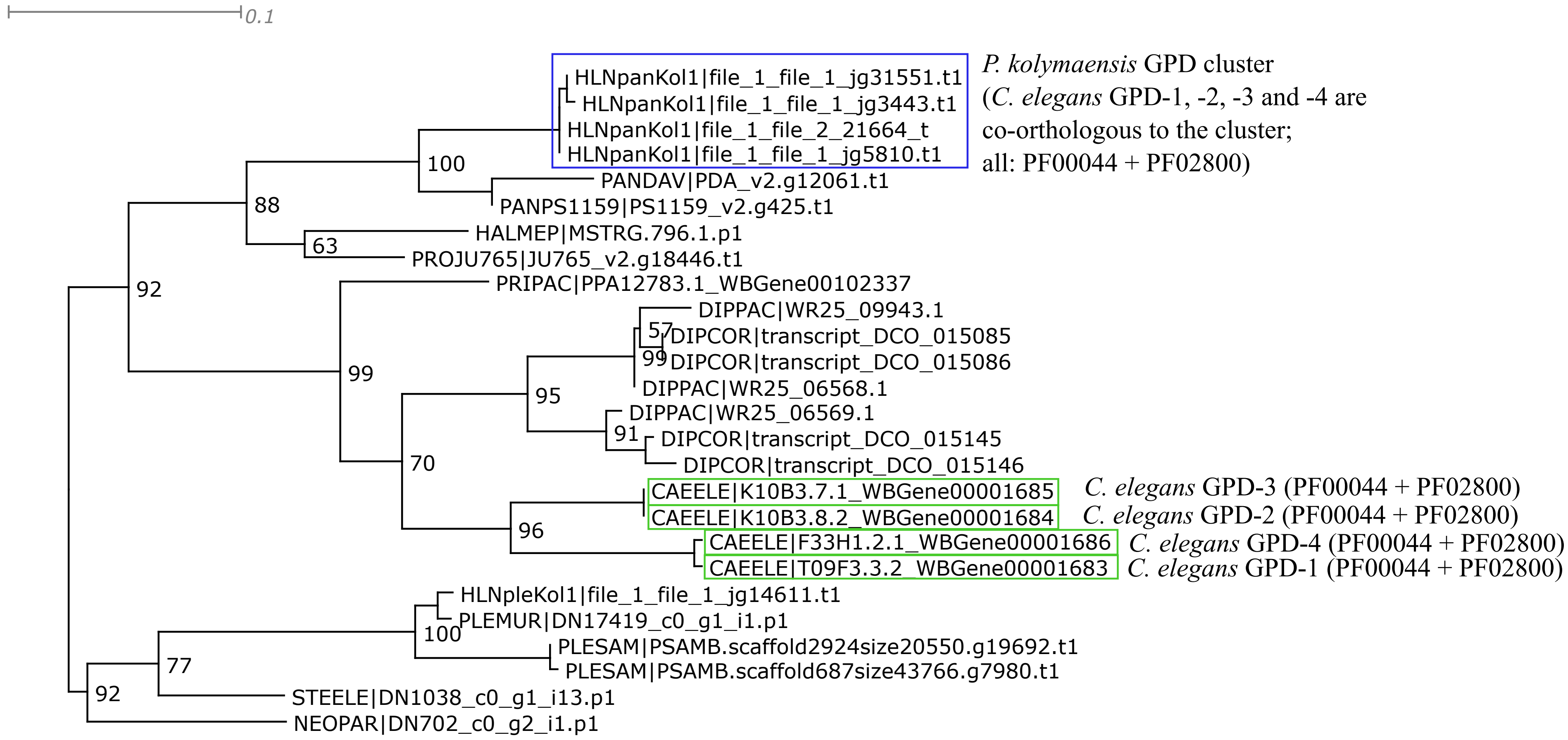

Trimal: 1. -resoverlap 0.75 -seqoverlap 80 functions; 2. -automated1 function; short or spurious sequences manually removed afterwards;
IQtree2 ML phylogeny best-fit model according to BIC: LG+G4

ALDO-1, ALDO-2

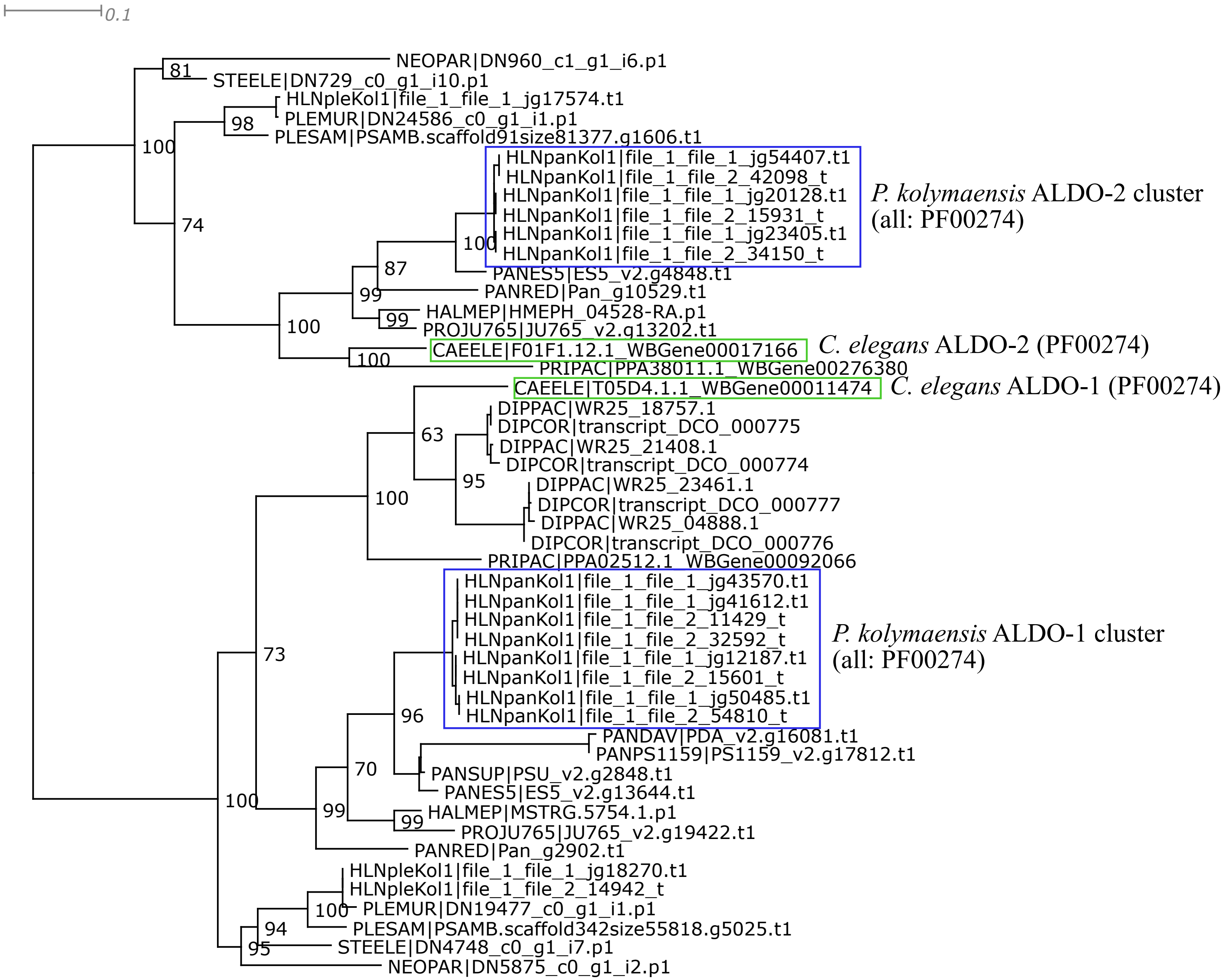

Trimal -automated1 function; short or spurious sequences manually removed afterwards;
IQtree2 ML phylogeny best-fit model according to BIC: LG+G4

FBP-1

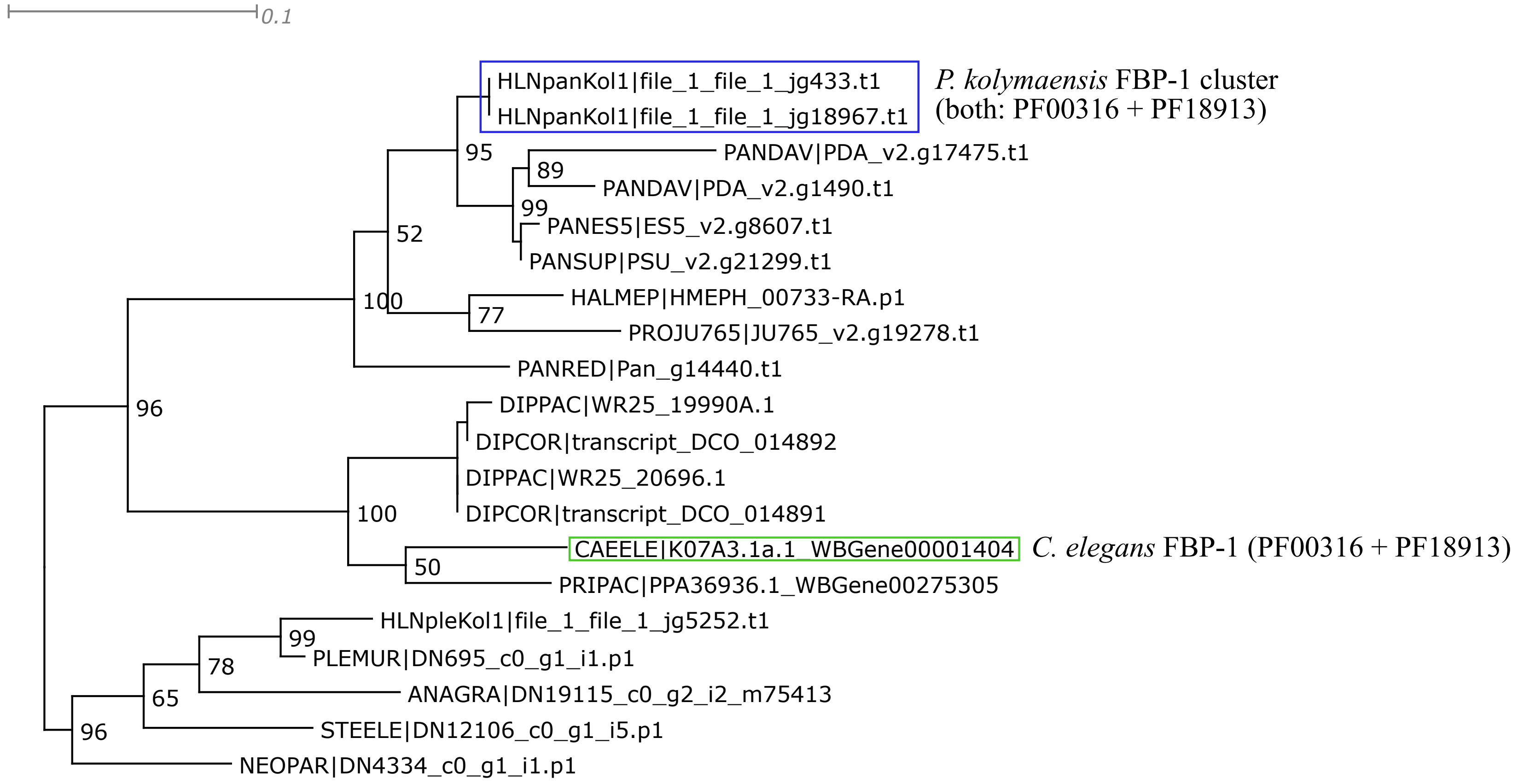

Trimal -automated1 function; short or spurious sequences manually removed afterwards;
IQtree2 ML phylogeny best-fit model according to BIC: WAG+G4

PFK-1

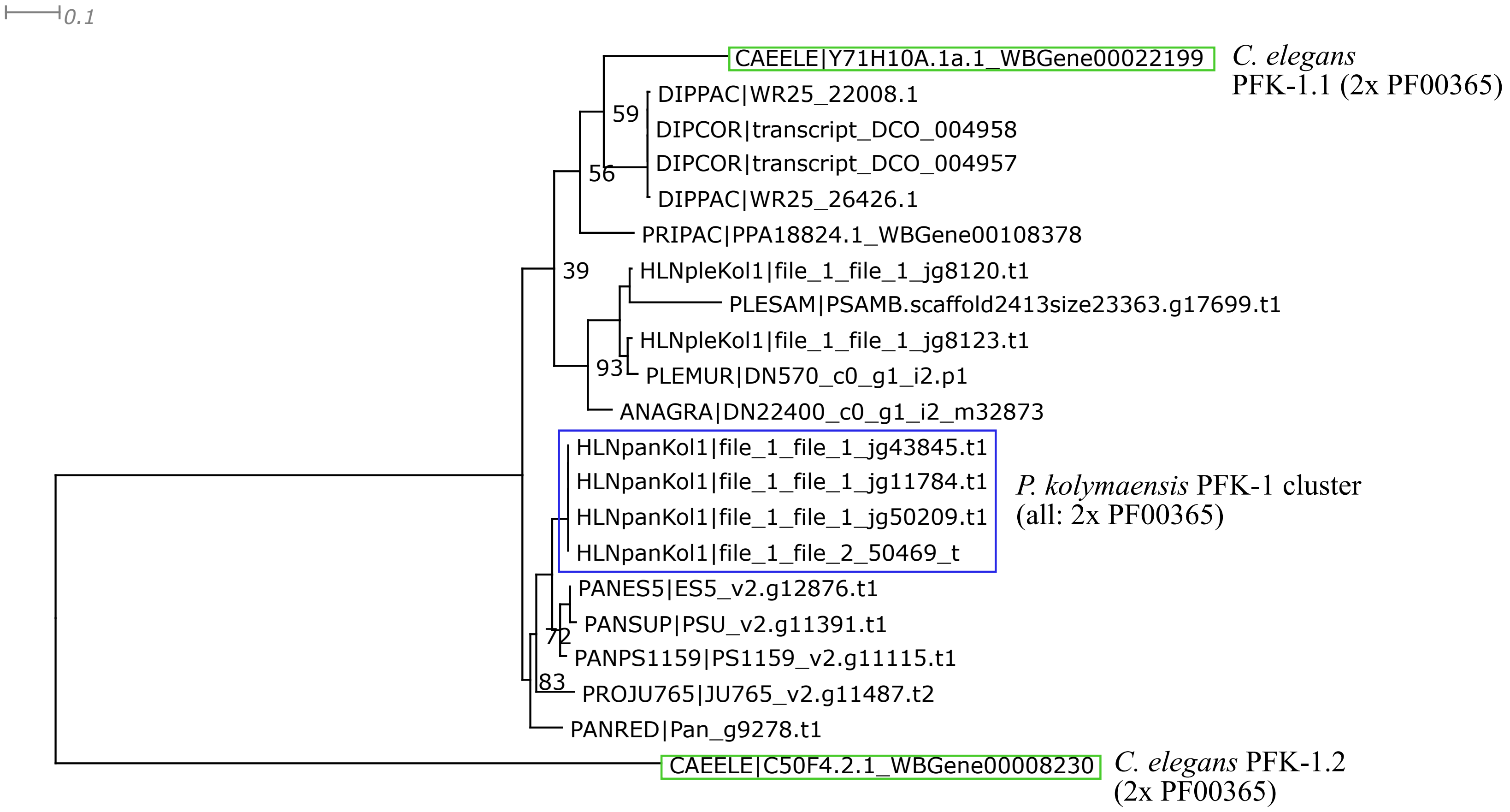

Trimal -automated1 function; short or spurious sequences (and sequences that disrupted phylogeny (N. parasiticus and H. mephisto) manually removed afterwards;
IQtree2 ML phylogeny best-fit model according to BIC: LG+G4

GPI-1

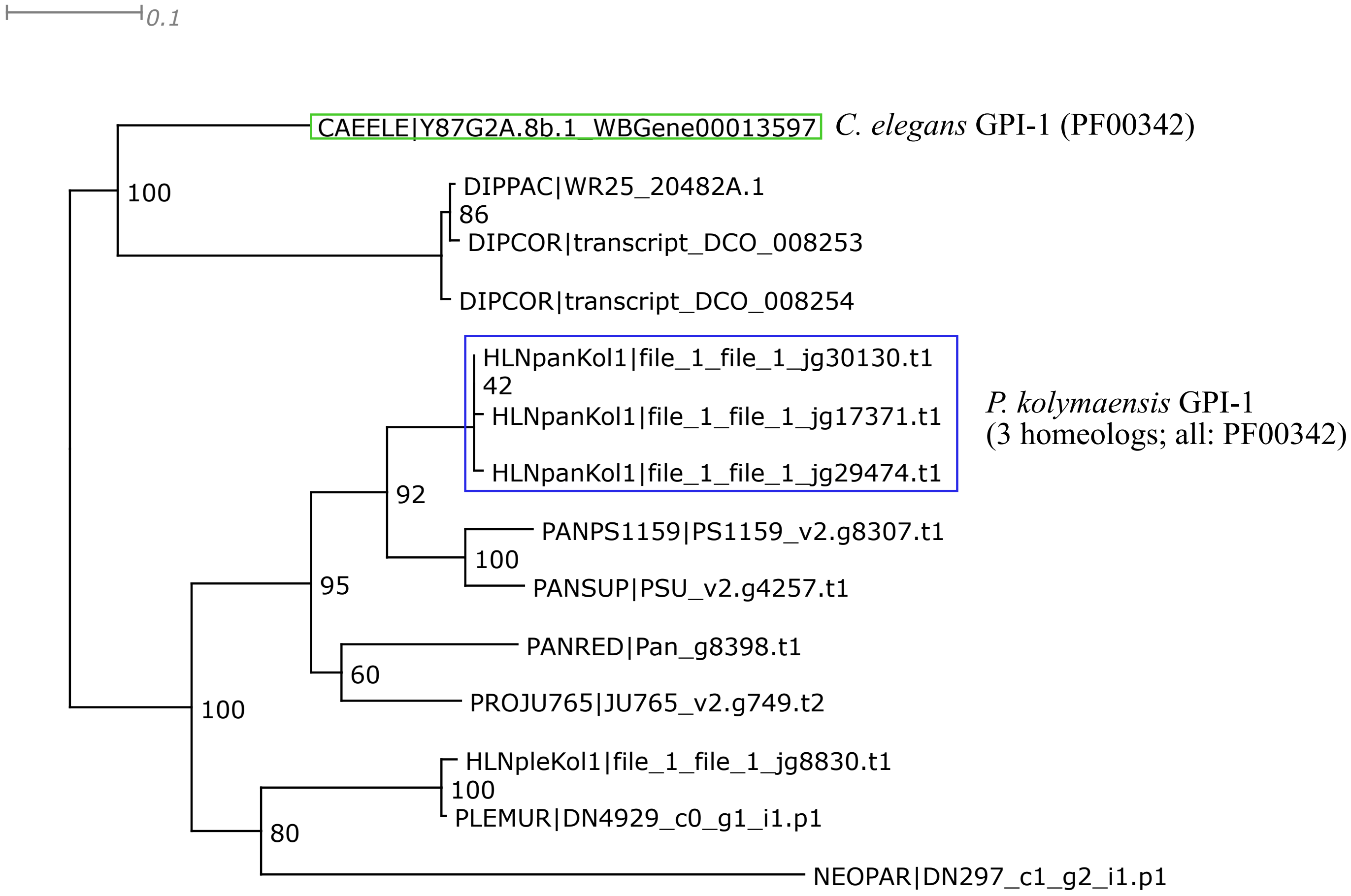

Trimal: 1. -resoverlap 0.75 -seqoverlap 80 functions; 2. -automated1 function; short or spurious sequences manually removed afterwards;
IQtree2 ML phylogeny best-fit model according to BIC: LG+G4

HXK-3

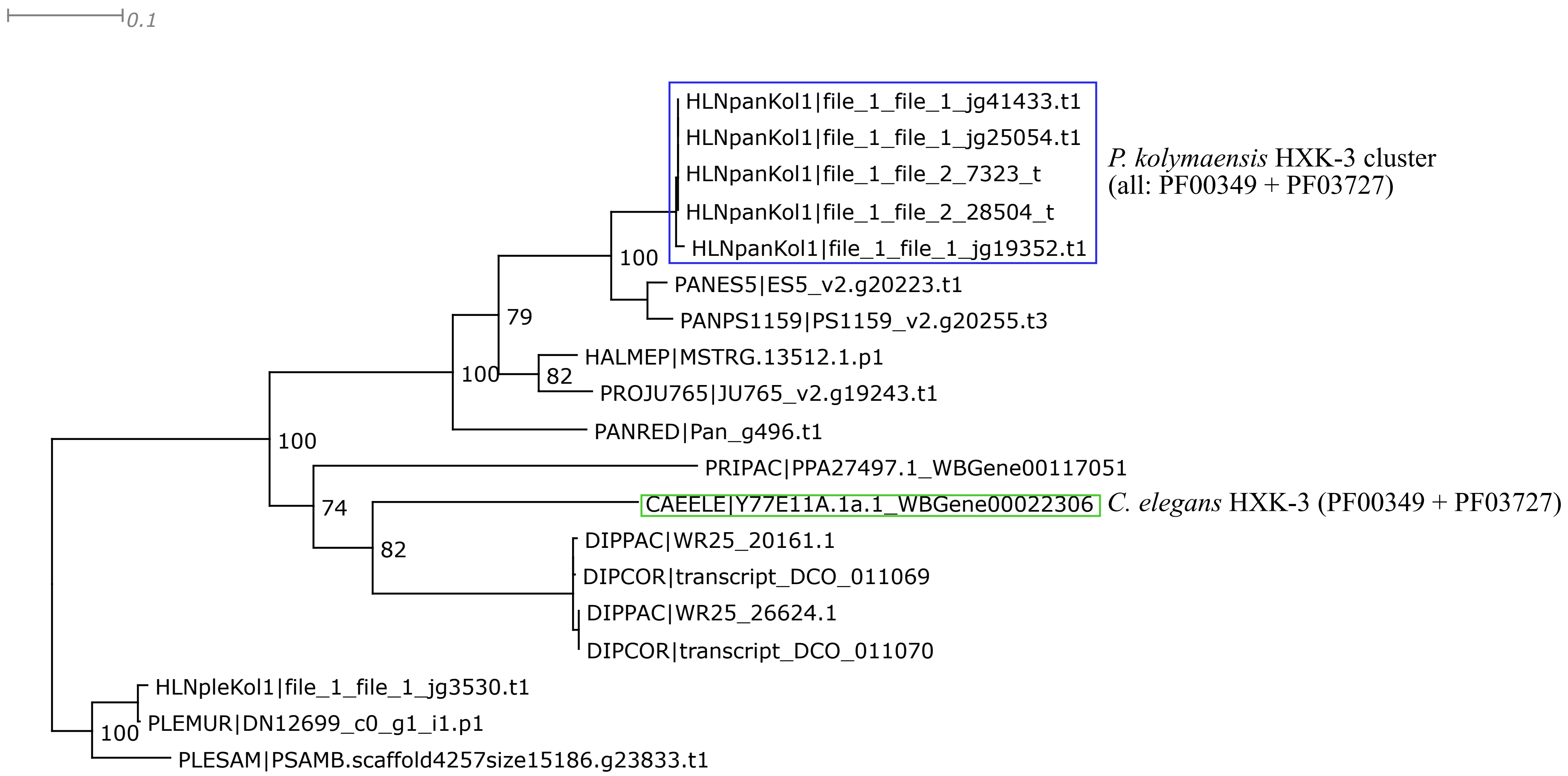

Trimal: 1. -resoverlap 0.75 -seqoverlap 80 functions; 2. -automated1 function; short or spurious sequences manually removed afterwards;
IQtree2 ML phylogeny best-fit model according to BIC: LG+G4

Polyamine biosynthesis

ODC-1

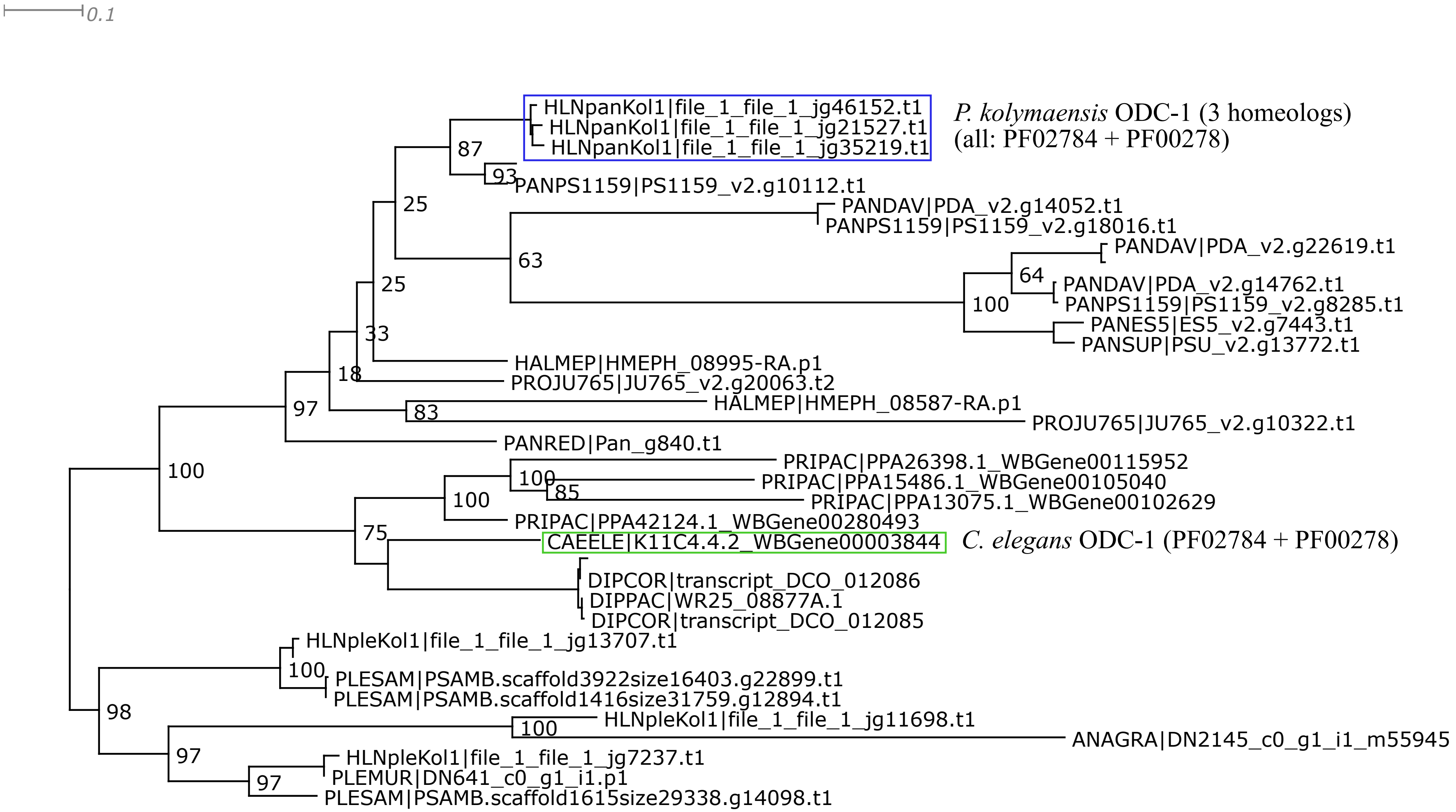

Trimal: 1. -resoverlap 0.75 -seqoverlap 80 functions; 2. -automated1 function; 3. short or spurious sequences manually removed afterwards; IQtree2 ML phylogeny best-fit model according to BIC: LG+I+G4

SPDS-1

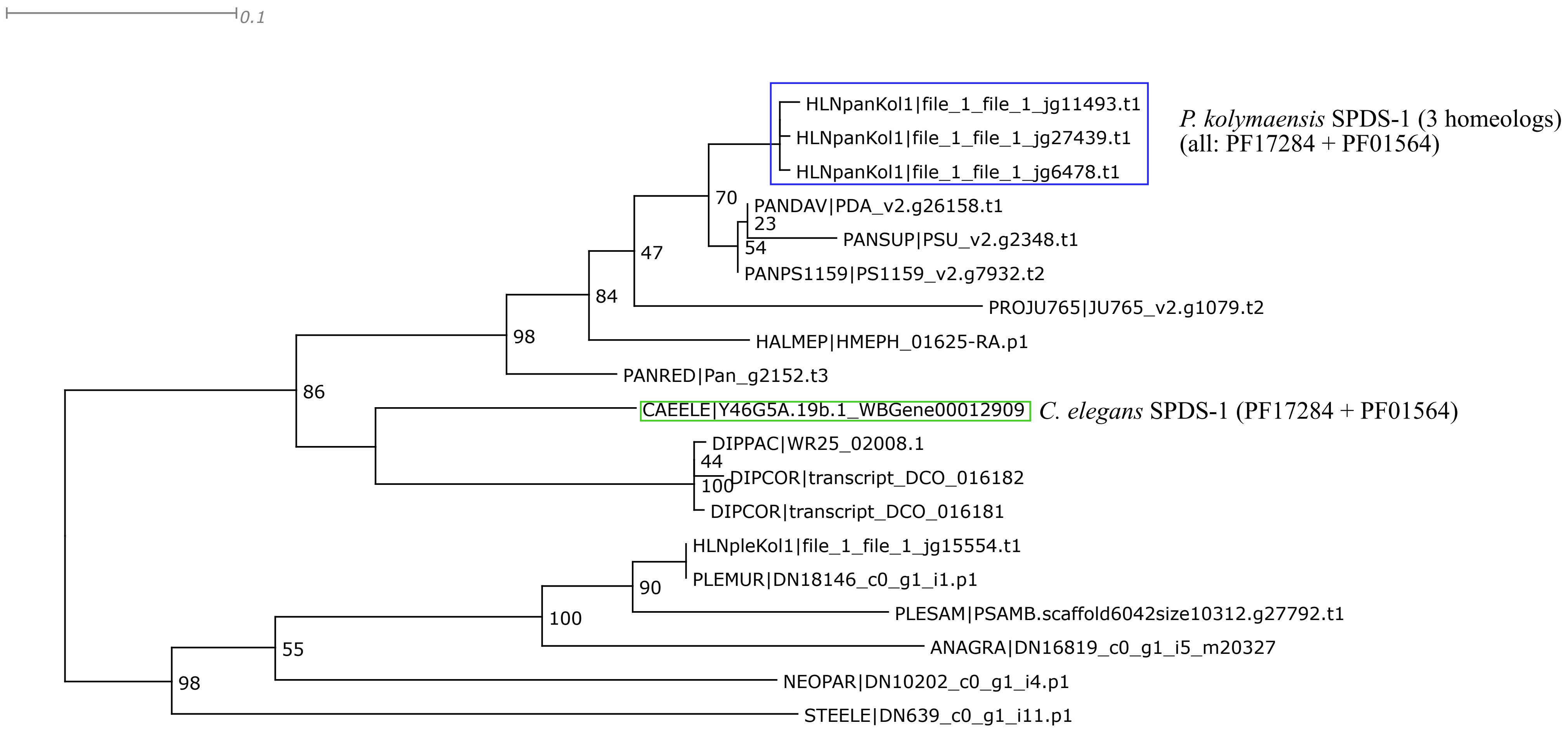

Trimal: 1. -resoverlap 0.75 -seqoverlap 80 functions; 2. -automated1 function; 3. short or spurious sequences manually removed afterwards; IQtree2 ML phylogeny best-fit model according to BIC: LG+G4

Dauer genes

DAF-1

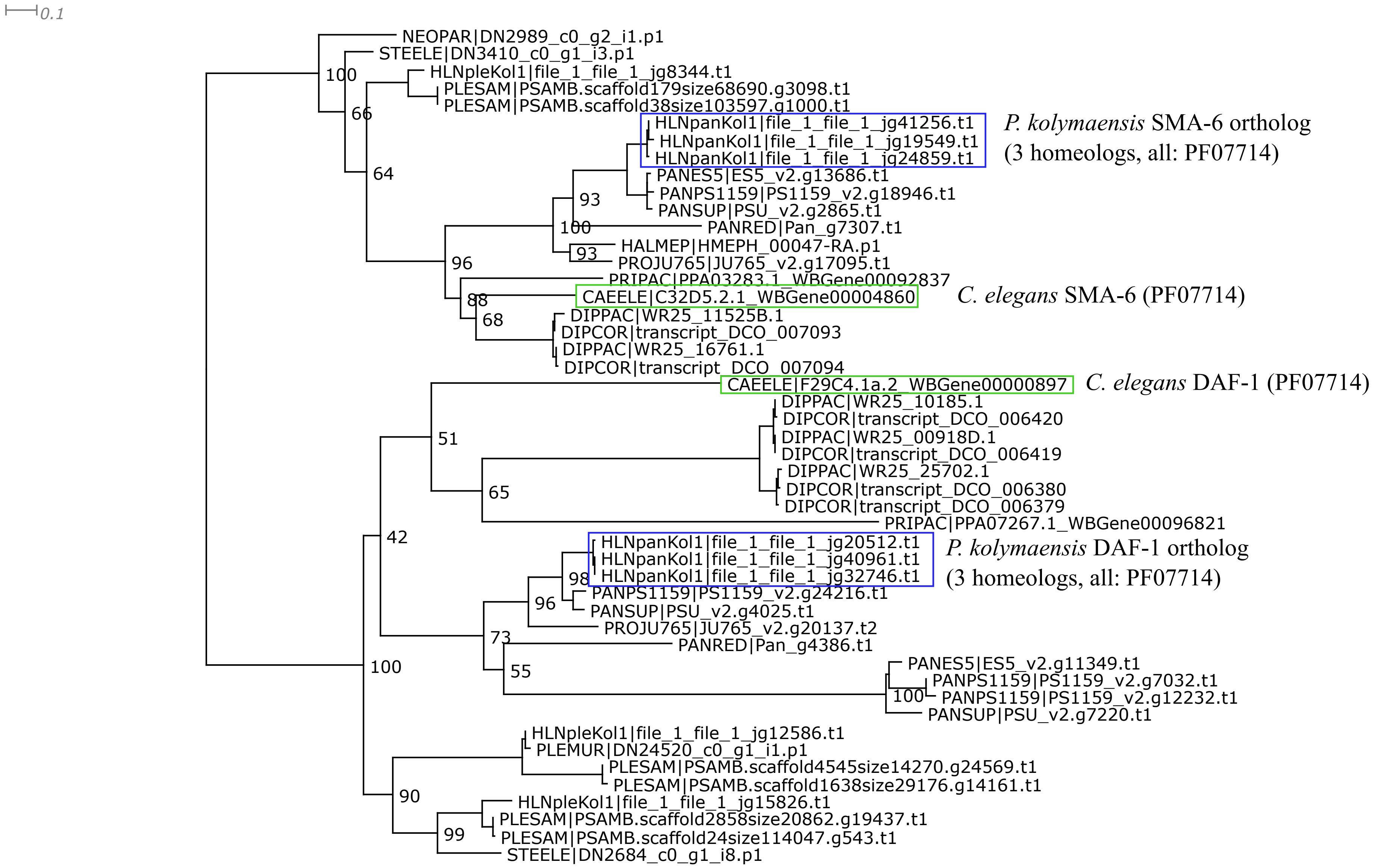

Trimal -automated1 function; short or spurious sequences manually removed afterwards;
IQtree2 ML phylogeny best-fit model according to BIC: LG+I+G4

DAF-2

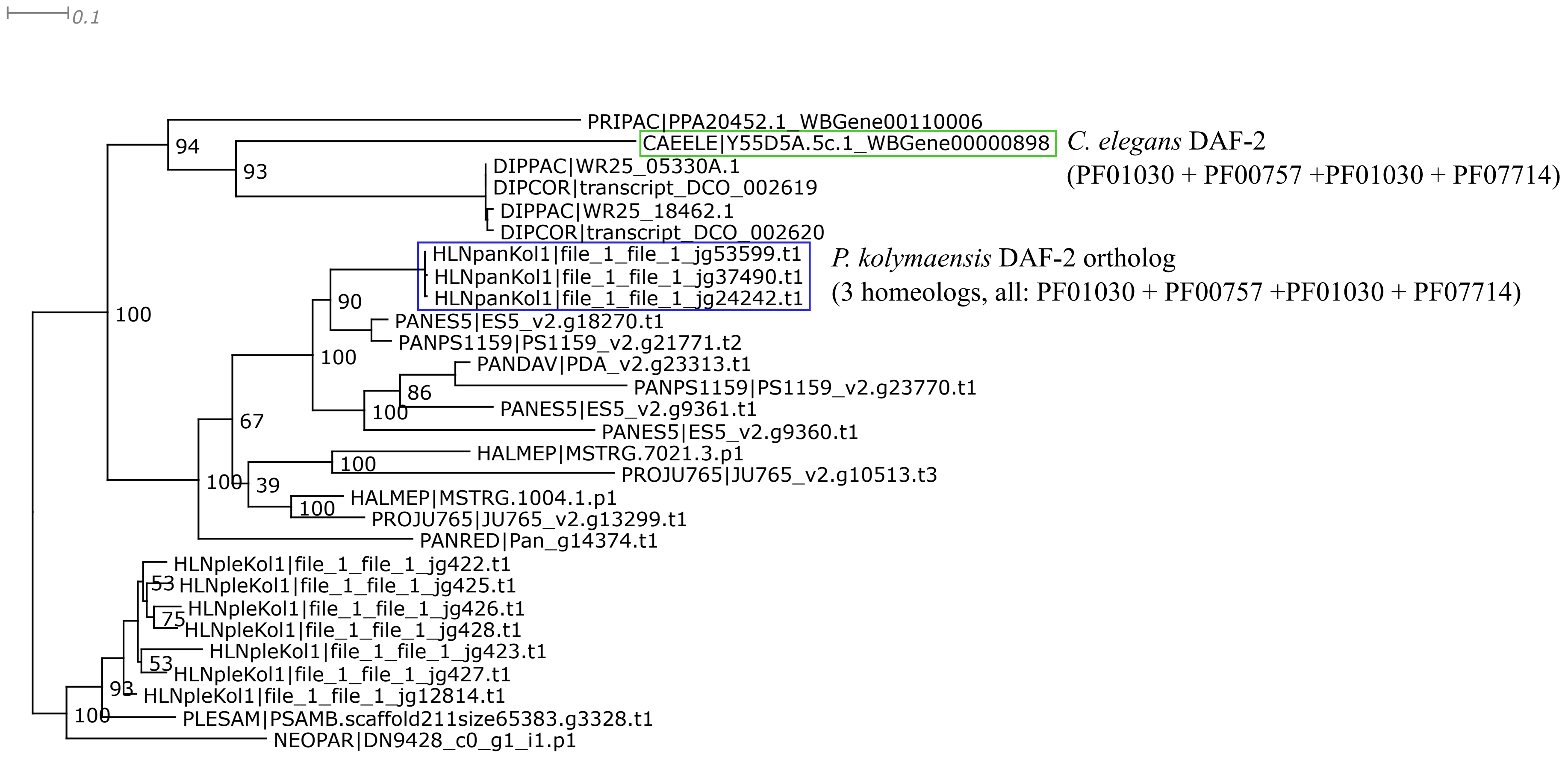

Trimal -automated1 function; Trimal functions -resoverlap 0.5 -seqoverlap 50;
IQtree2 ML phylogeny best-fit model according to BIC: LG+I+G4

DAF-4

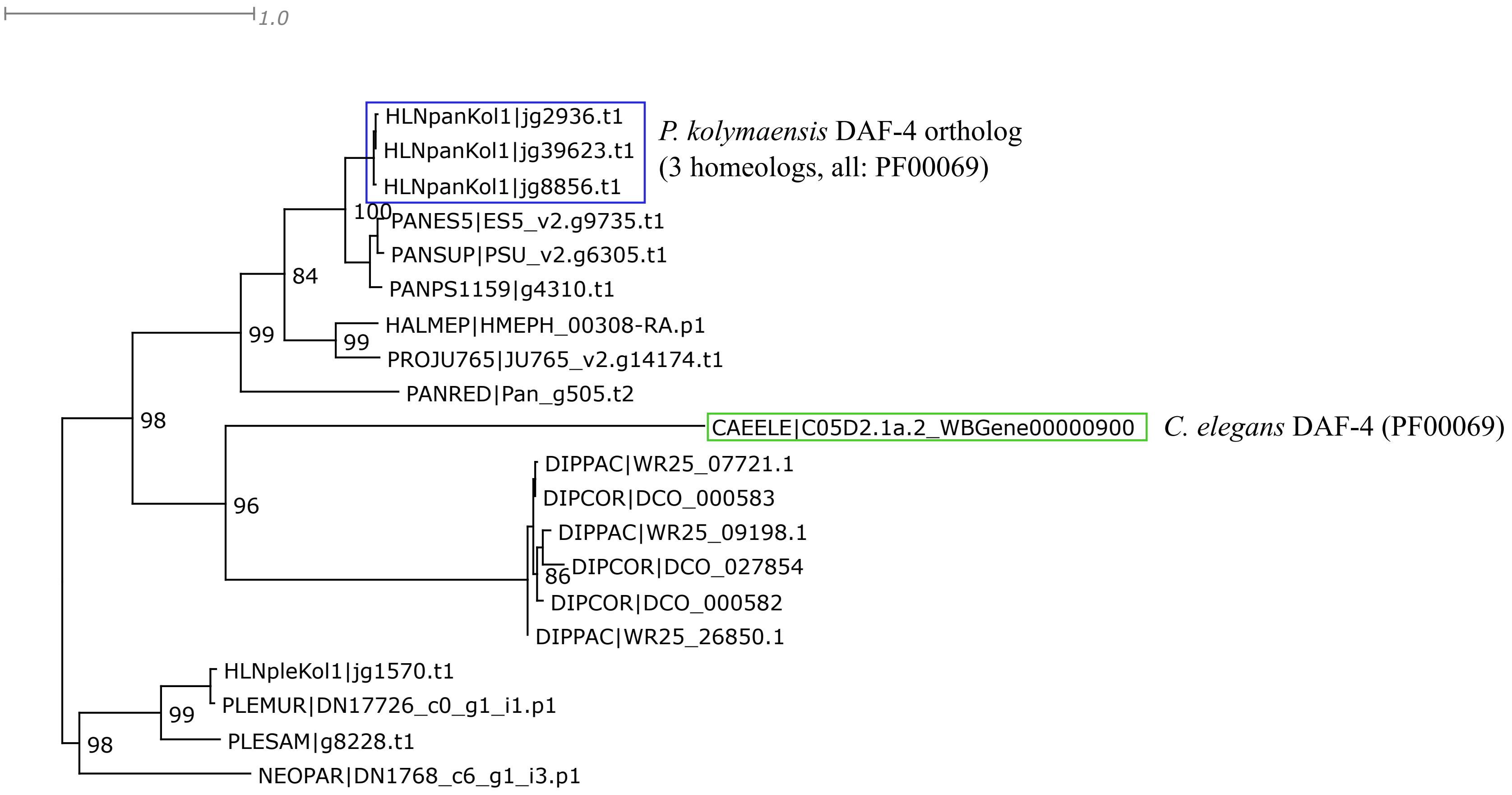

Trimal -automated1 function; short or spurious sequences manually removed afterwards;
IQtree2 ML phylogeny best-fit model according to BIC: LG+I+G4

DAF-6

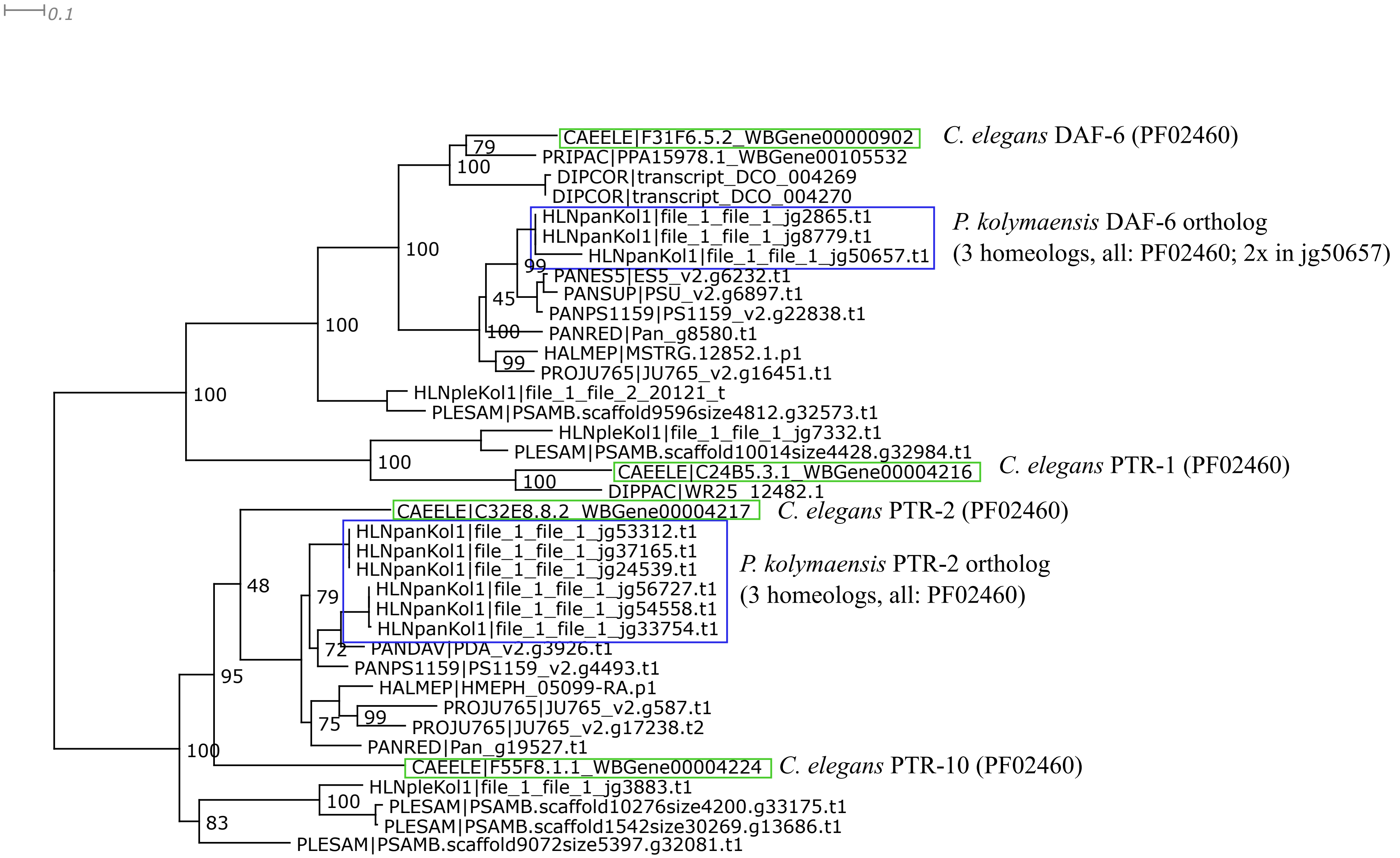

Trimal -automated1 function; Trimal function -resoverlap 0.75 -seqoverlap 75; more short or spurious sequences manually removed afterwards; IQtree2 ML phylogeny best-fit model according to BIC: LG+G4

DAF-7

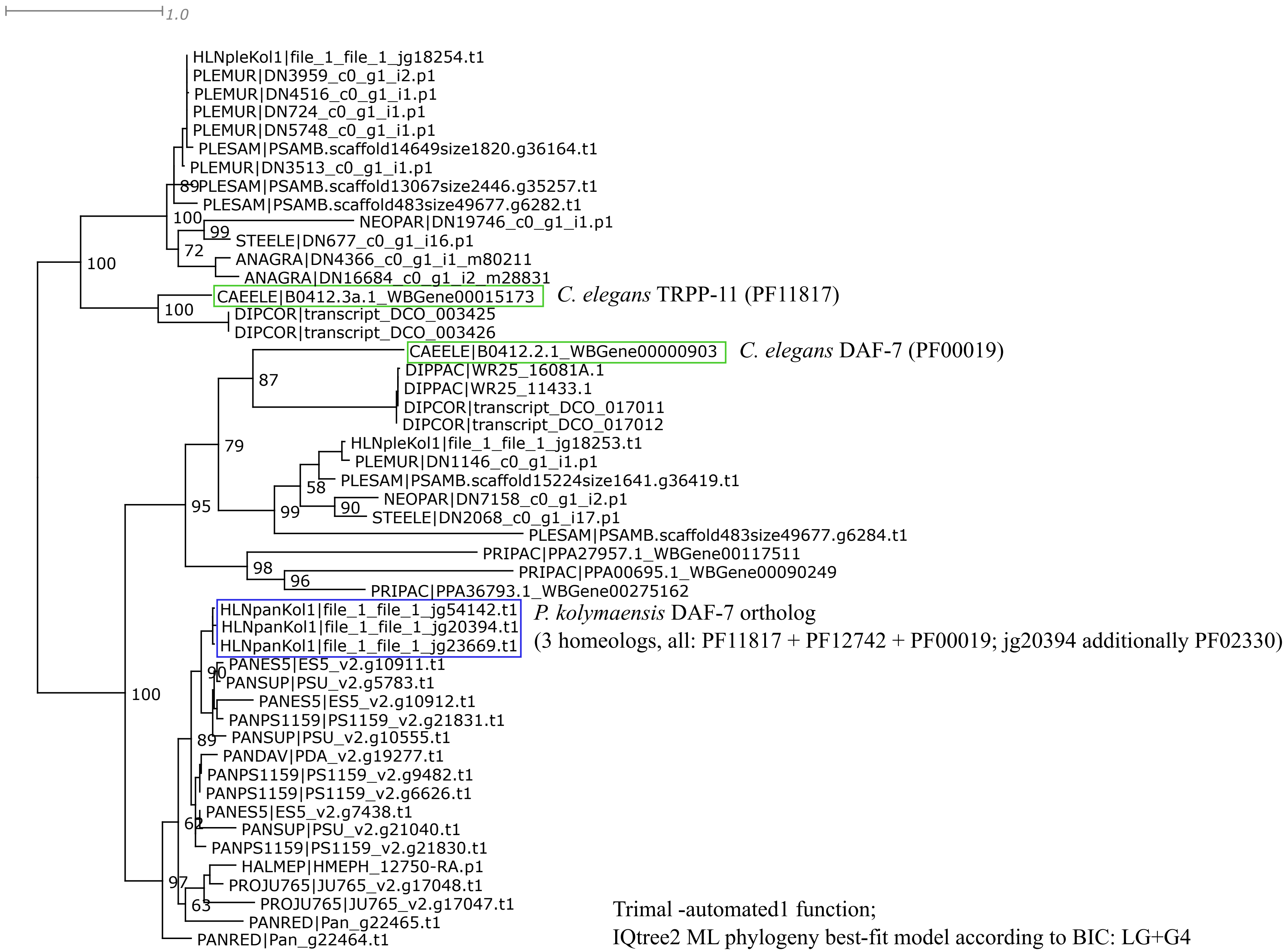

Trimal -automated1 function;
IQtree2 ML phylogeny best-fit model according to BIC: LG+G4

DAF-8 and DAF-14

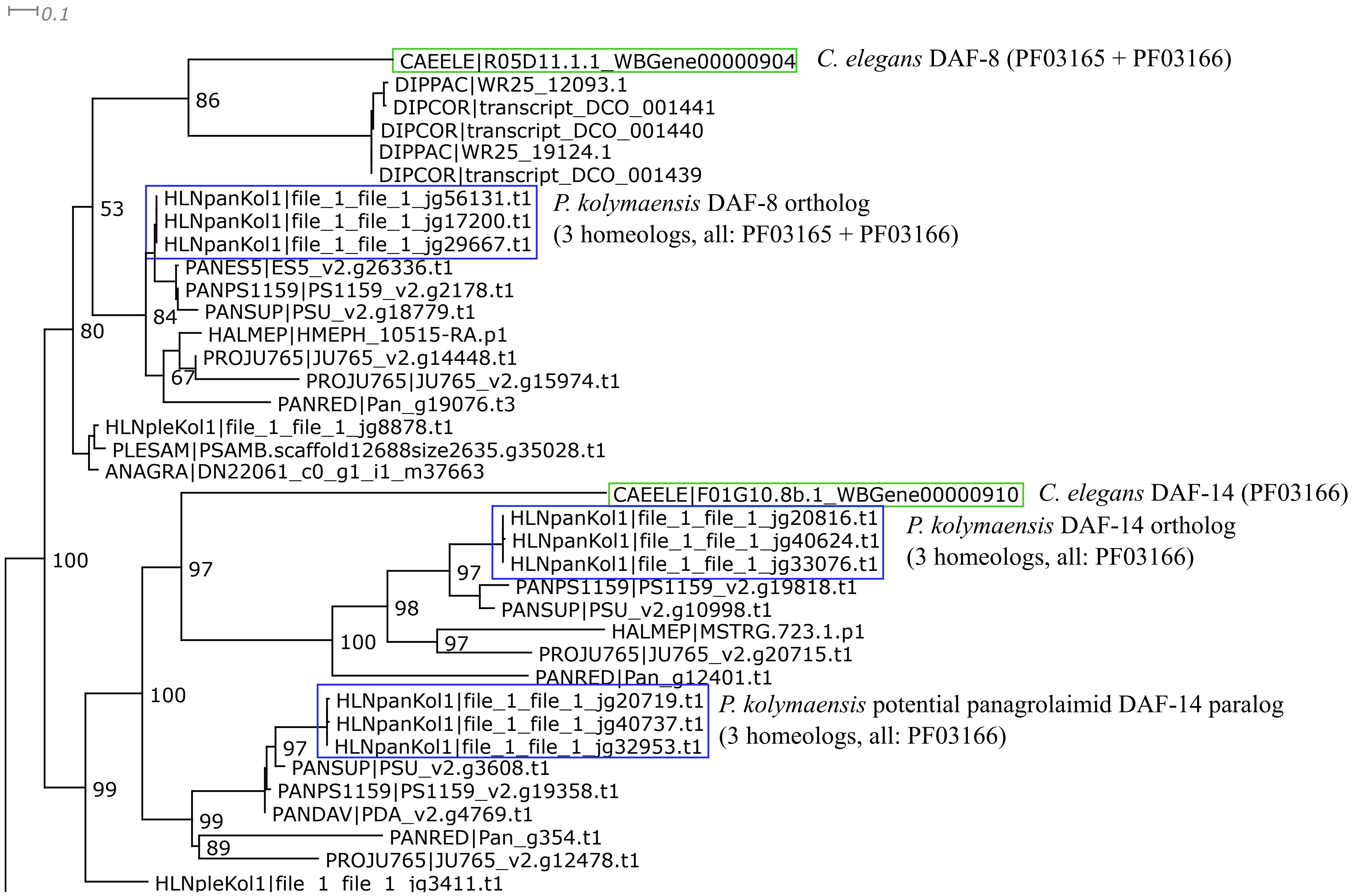

Partial phylogeny; clusters not shown here: SMA-2 orthologs, and SMA-3 orthologs;
Trimal -automated1 function; short and divergent sequences removed manually
IQtree2 ML phylogeny best-fit model according to BIC: LG+I+G4

DAF-9

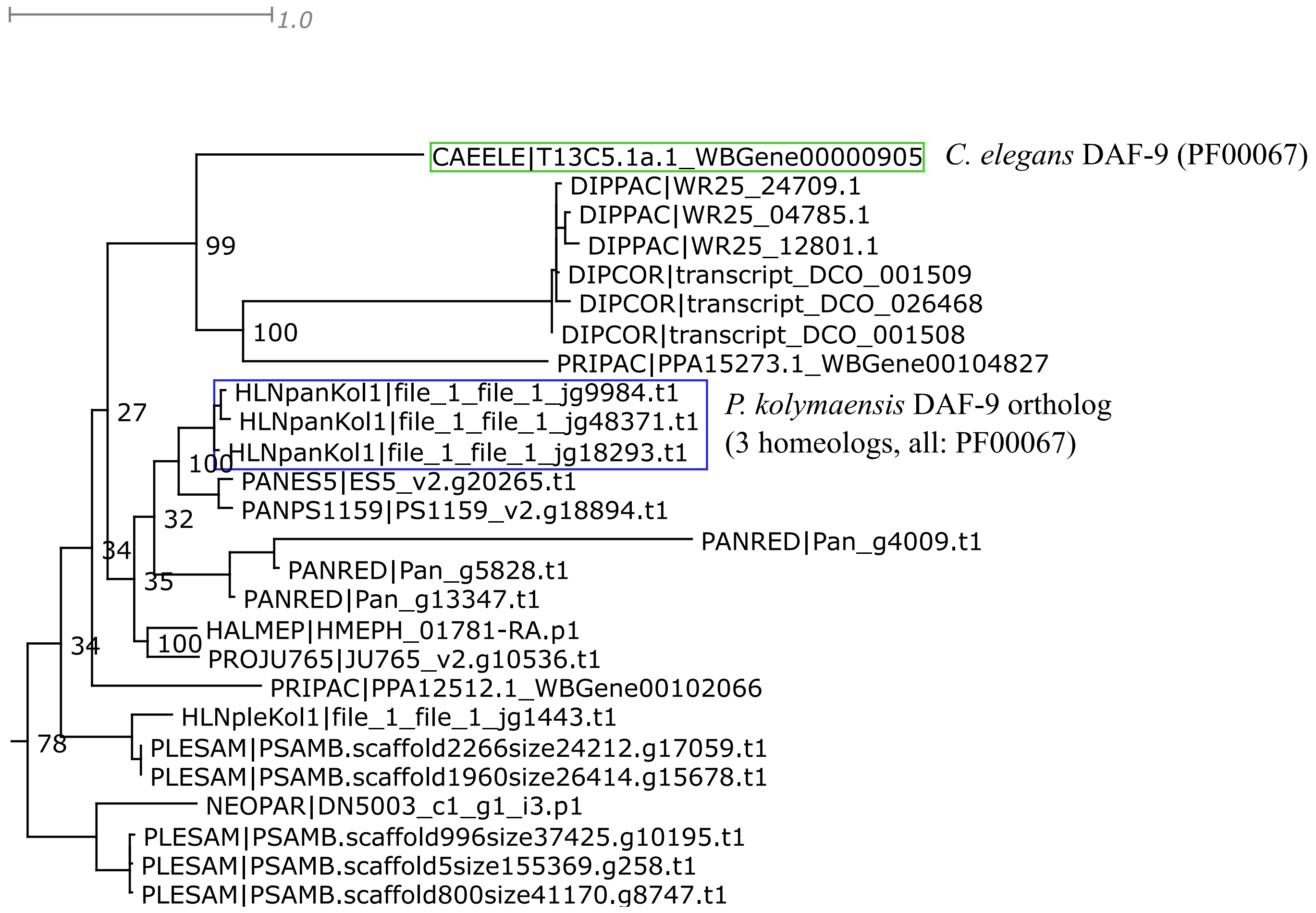

Partial phylogeny; the DAF-9 cluster is only one of many clusters in a large phylogeny of cytochrome P450 genes;
Trimal -automated1 function; short and divergent sequences removed manually
IQtree2 ML phylogeny best-fit model according to BIC: LG+G4

DAF-10

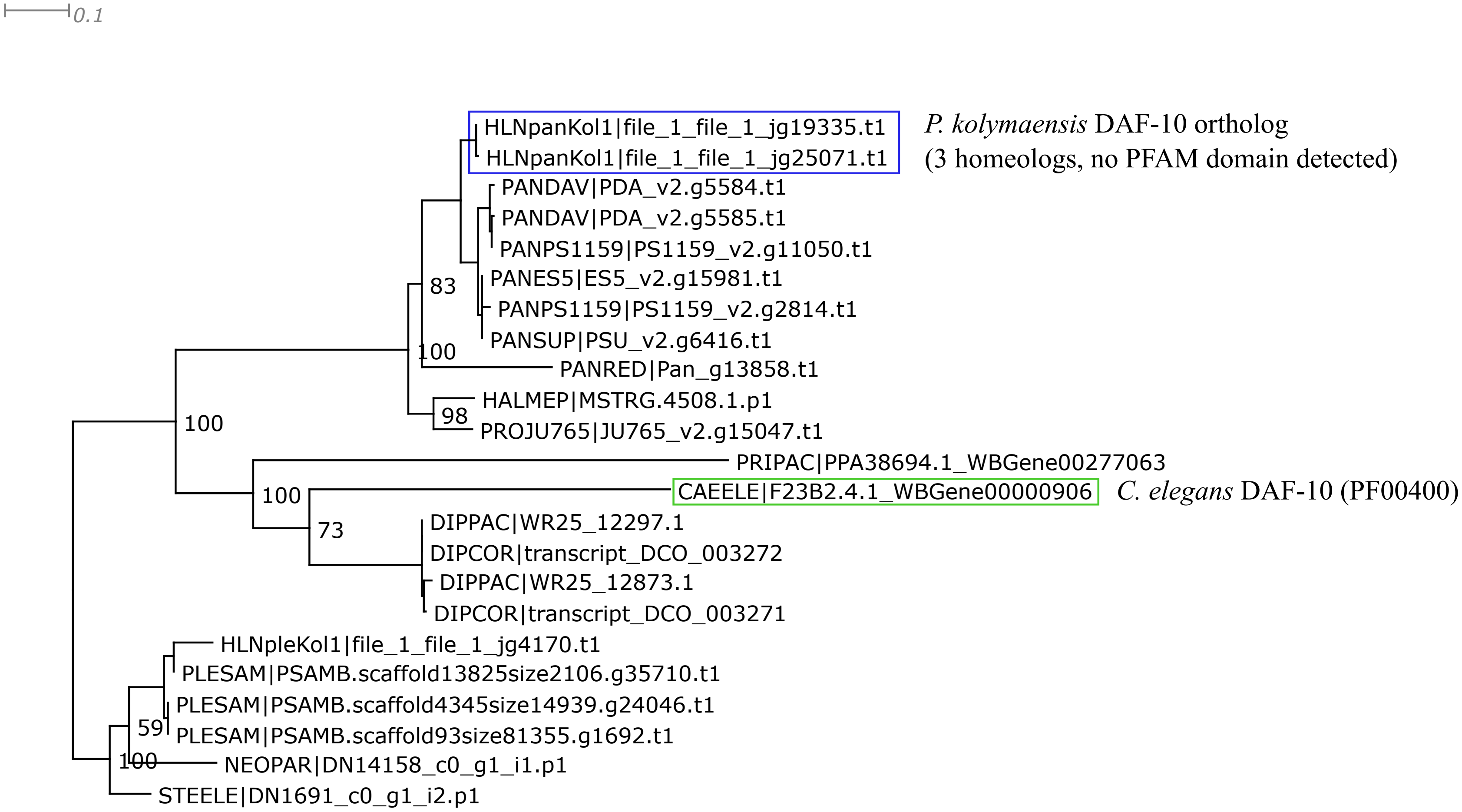

Trimal -automated1 function; .
IQtree2 ML phylogeny best-fit model according to BIC: LG+G4
The PFAM domain PF00400 found in the *C. elegans* DAF-10 protein is not detected in many panagrolaimids, but in some (*P. redivivus*, *Propanagrolaimus* sp. JU765, *H. mephisto*), it might be diverged and not recognised.

DAF-11

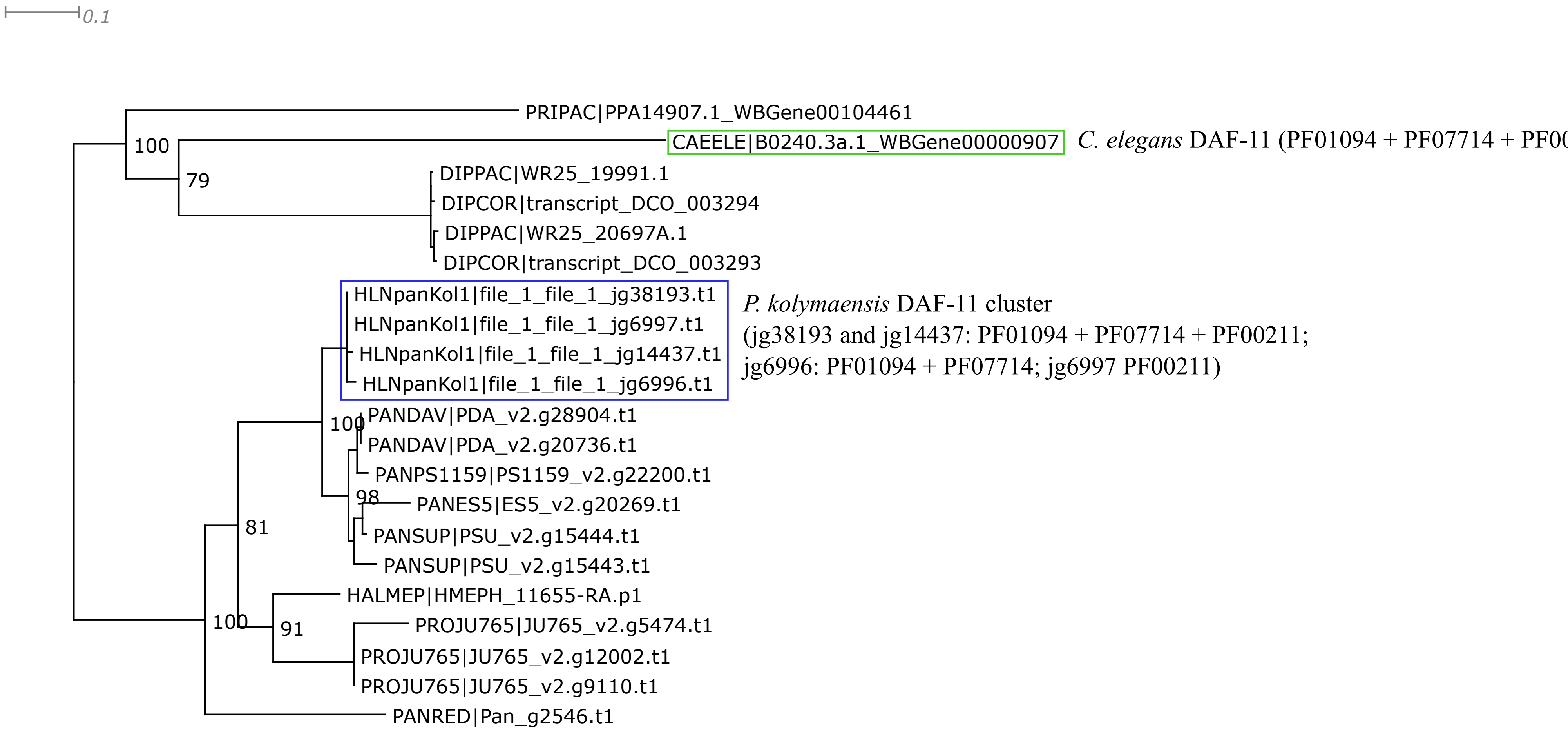

Trimal -automated1 function; .
IQtree2 ML phylogeny best-fit model according to BIC: LG+I+G4.

DAF-12

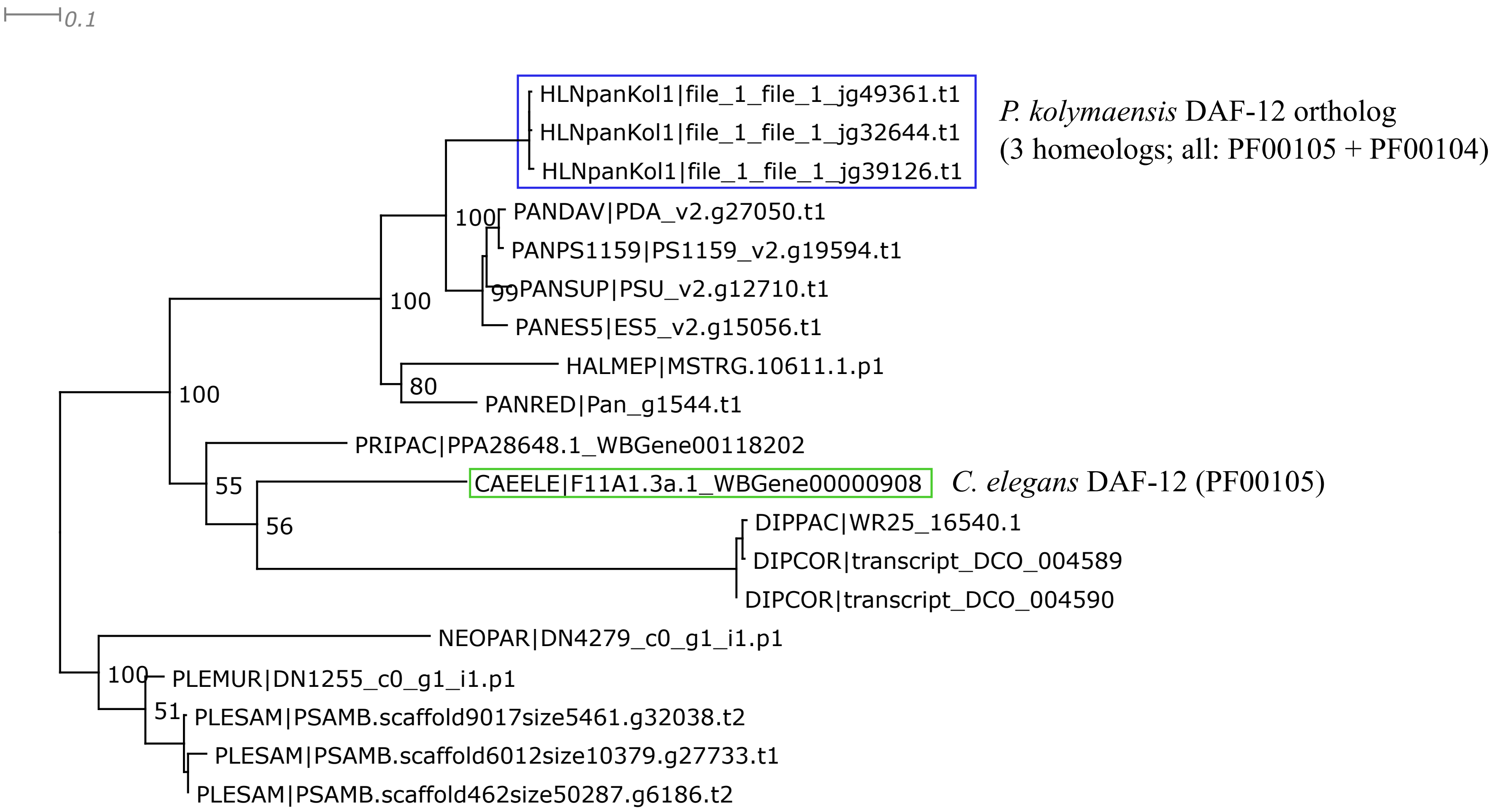

Trimal -automated1 function; short and spurious sequences removed manually.
IQtree2 ML phylogeny best-fit model according to BIC: LG+G4.

DAF-15

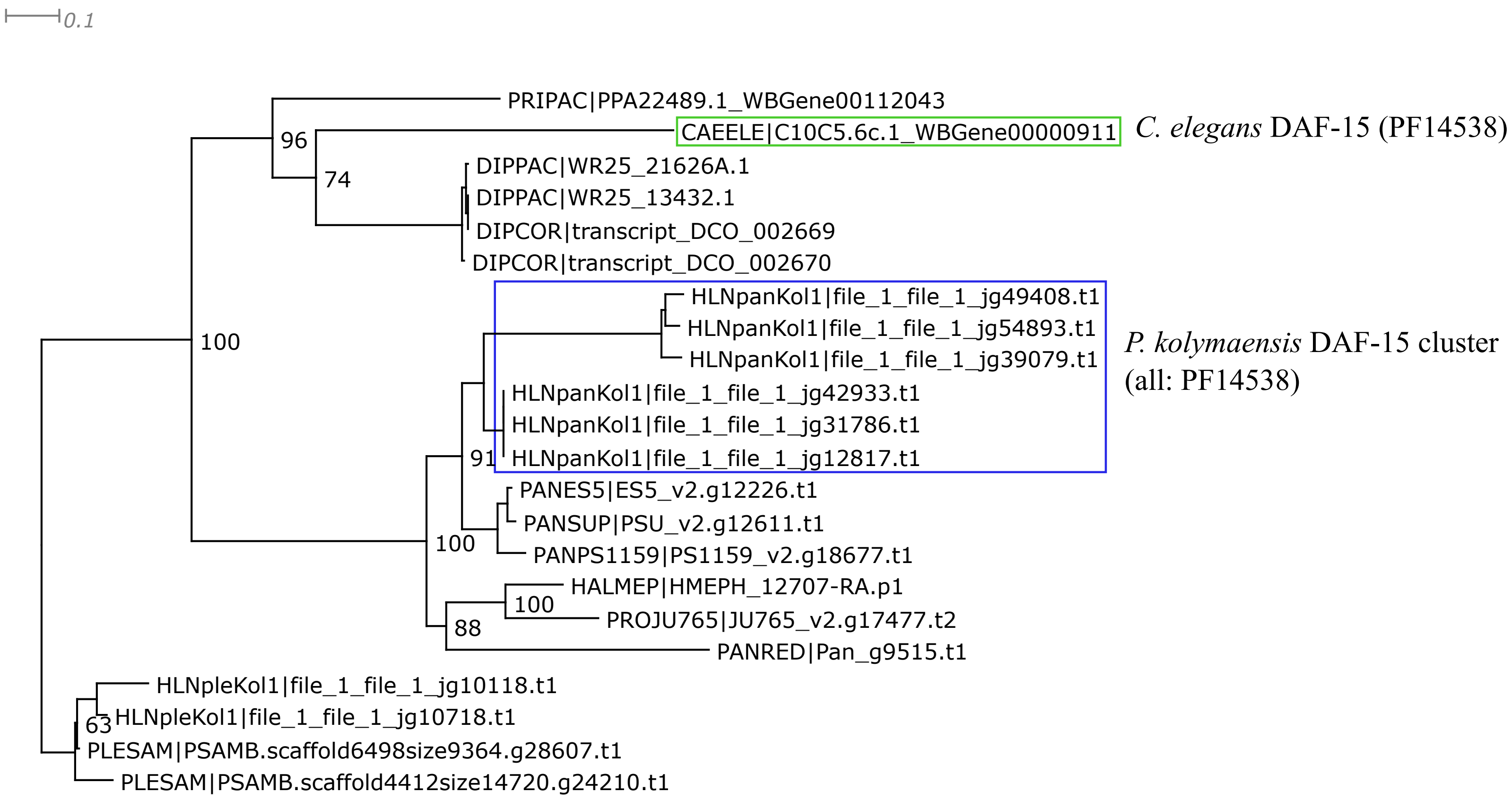

Trimal automated1 function; short and spurious sequences removed manually.
IQtree2 ML phylogeny best-fit model according to BIC: LG+G4.

DAF-16

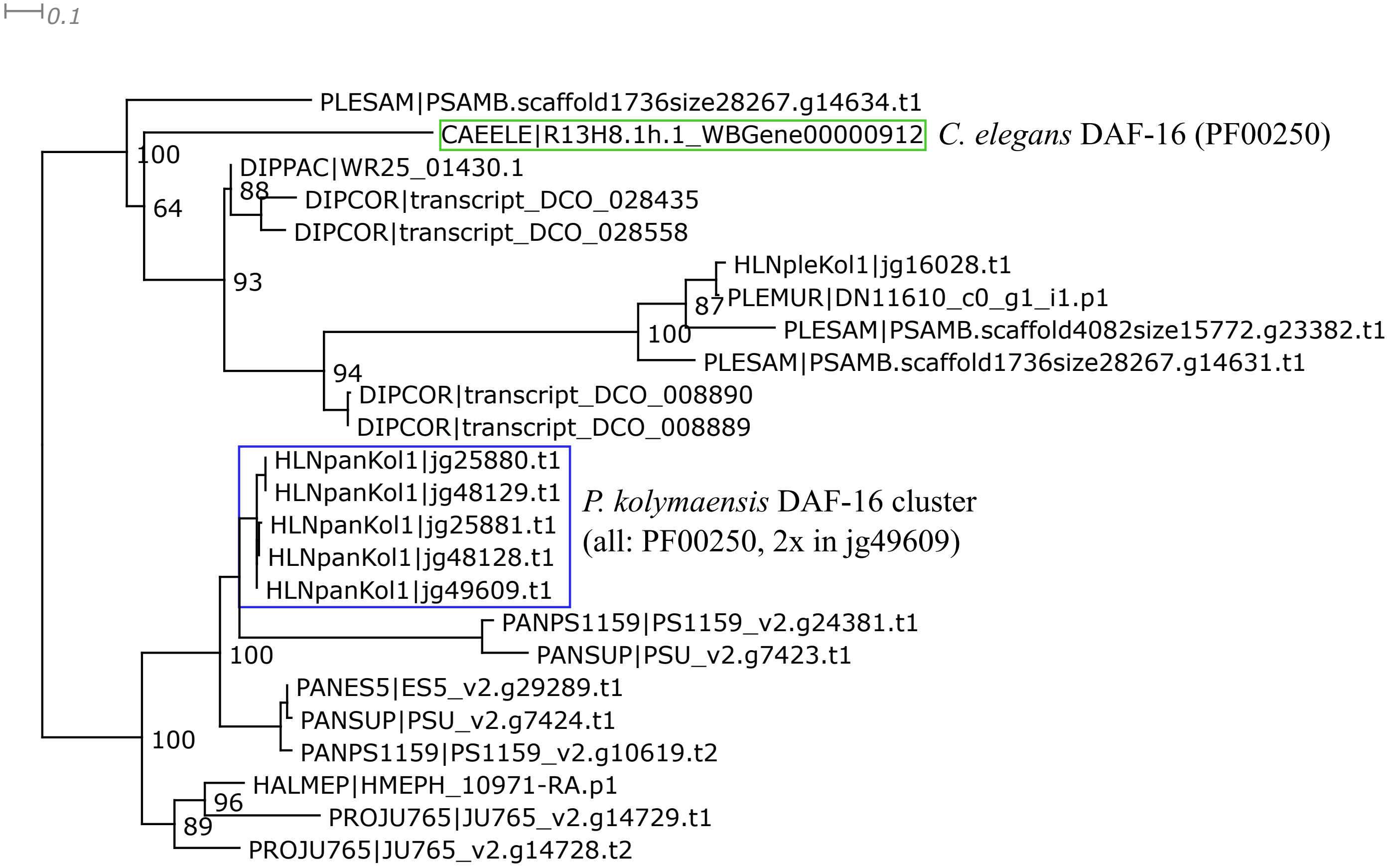

Short and spurious sequences removed manually.
IQtree2 ML phylogeny best-fit model according to BIC: VT+F+G4.

DAF-18

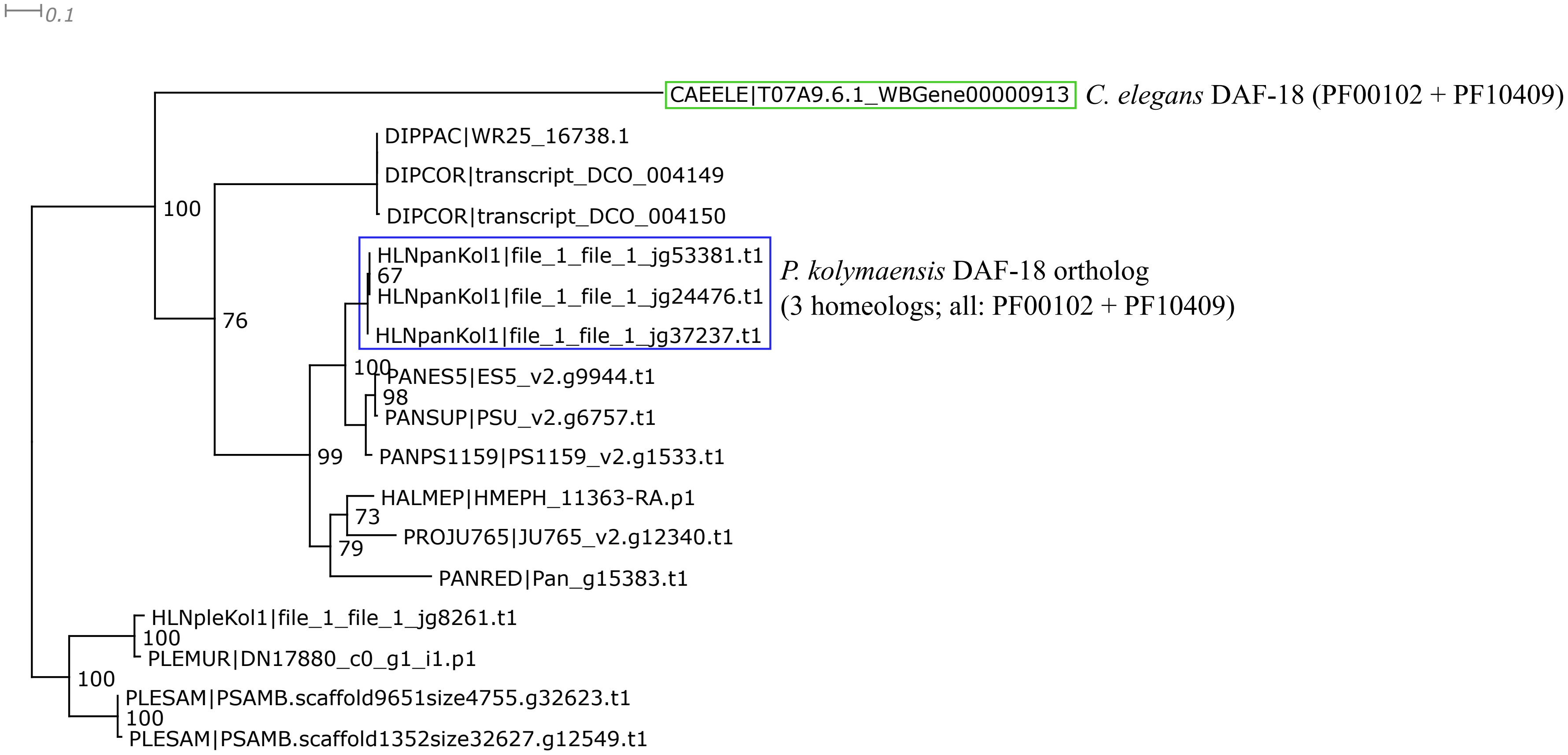

Trimal automated1 function: Trimal functions -resoverlap 0.75 -seqoverlap 75.
IQtree2 ML phylogeny best-fit model according to BIC: LG+I+G4.

DAF-19

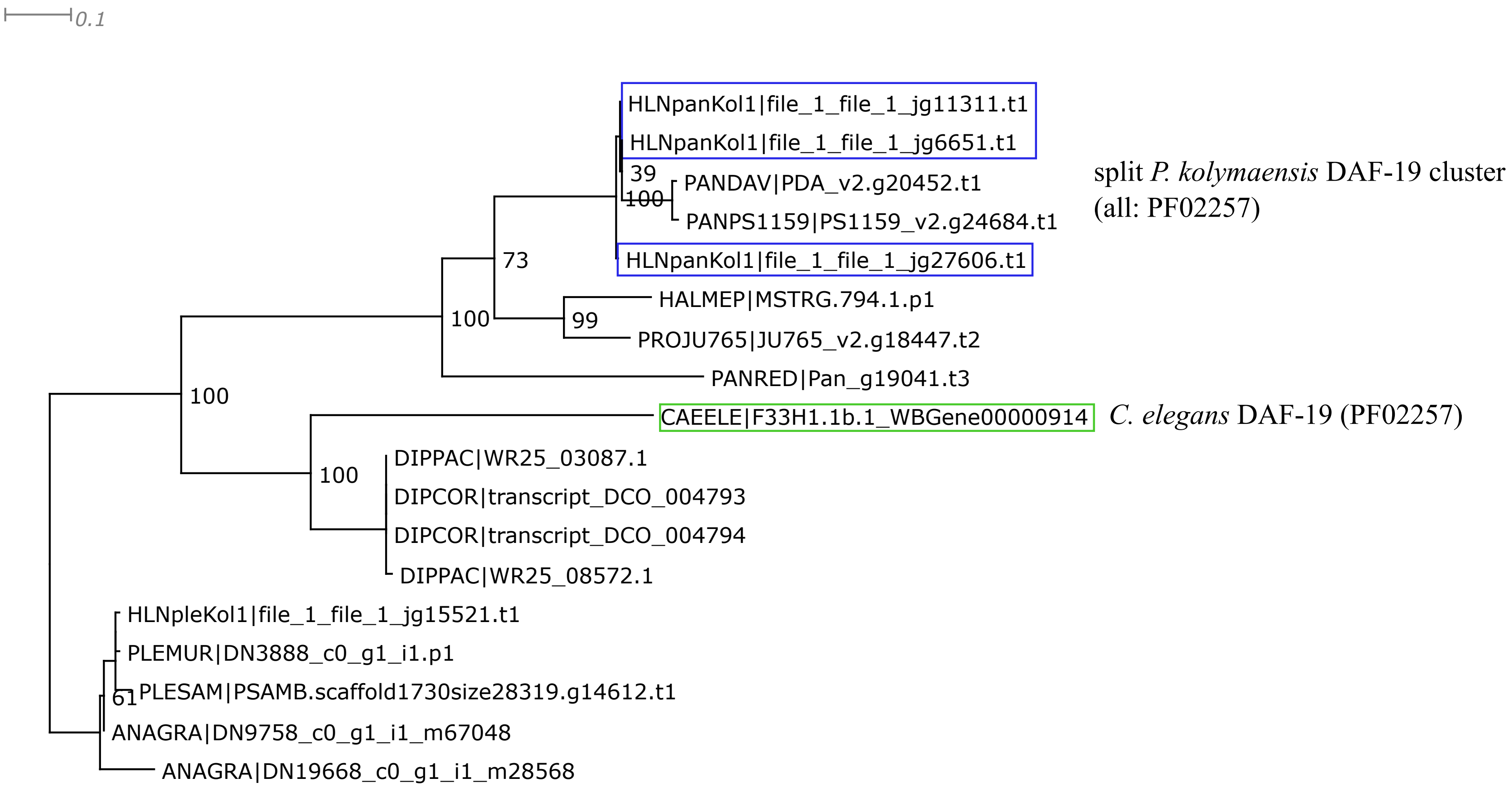

Trimal automated1 function; Short and spurious sequences removed manuallly.
IQtree2 ML phylogeny best-fit model according to BIC: LG+G4.

DAF-21

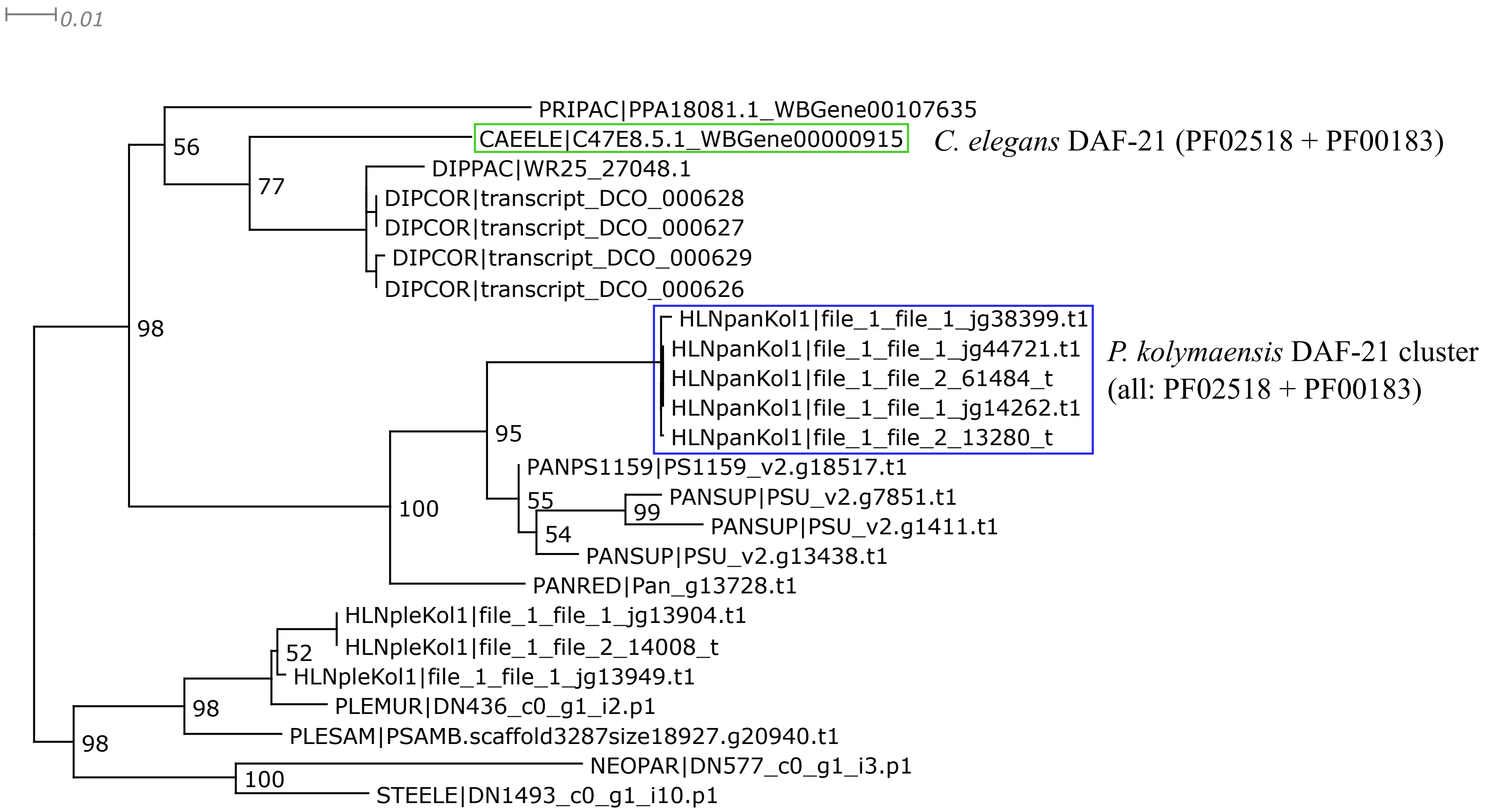

Trimal automated1 function; Short and spurious sequences removed manuallly.
IQtree2 ML phylogeny best-fit model according to BIC: LG+G4.

DAF-22

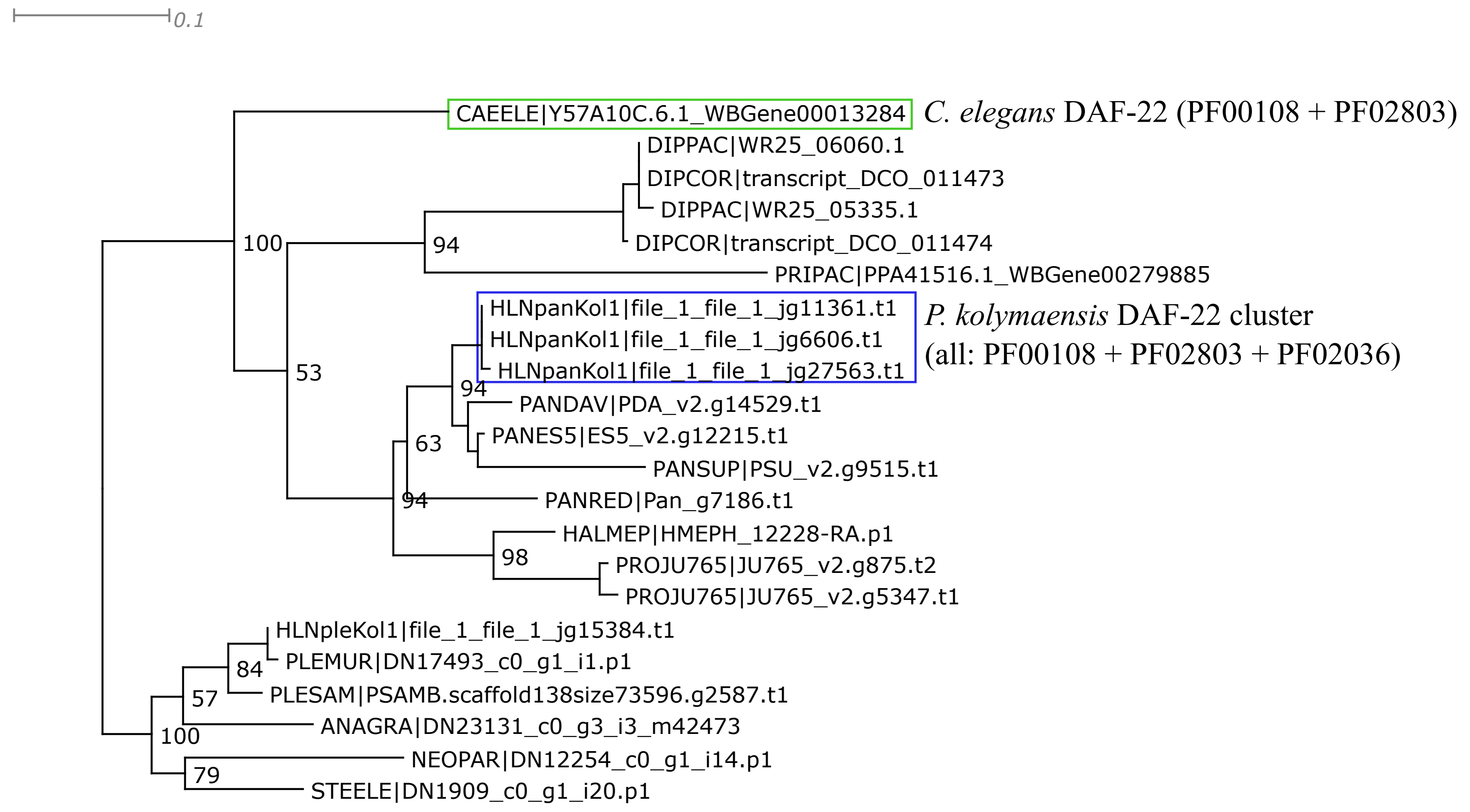

Trimal automated1 function; Short and spurious sequences removed manuallly.
IQtree2 ML phylogeny best-fit model according to BIC: LG+G4.

DAF-25

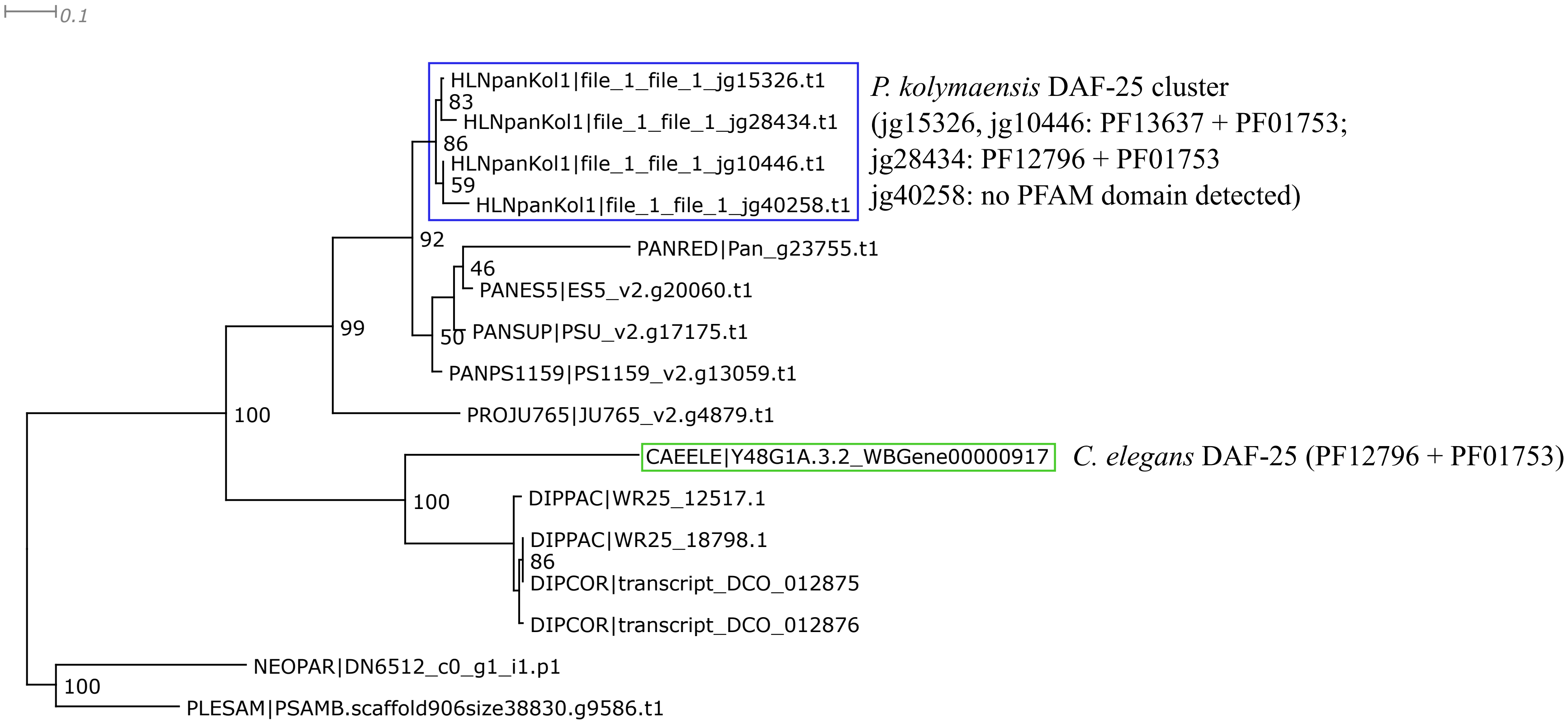

Trimal automated1 function; Short and spurious sequences removed manuallly.
IQtree2 ML phylogeny best-fit model according to BIC: LG+G4.

DAF-31

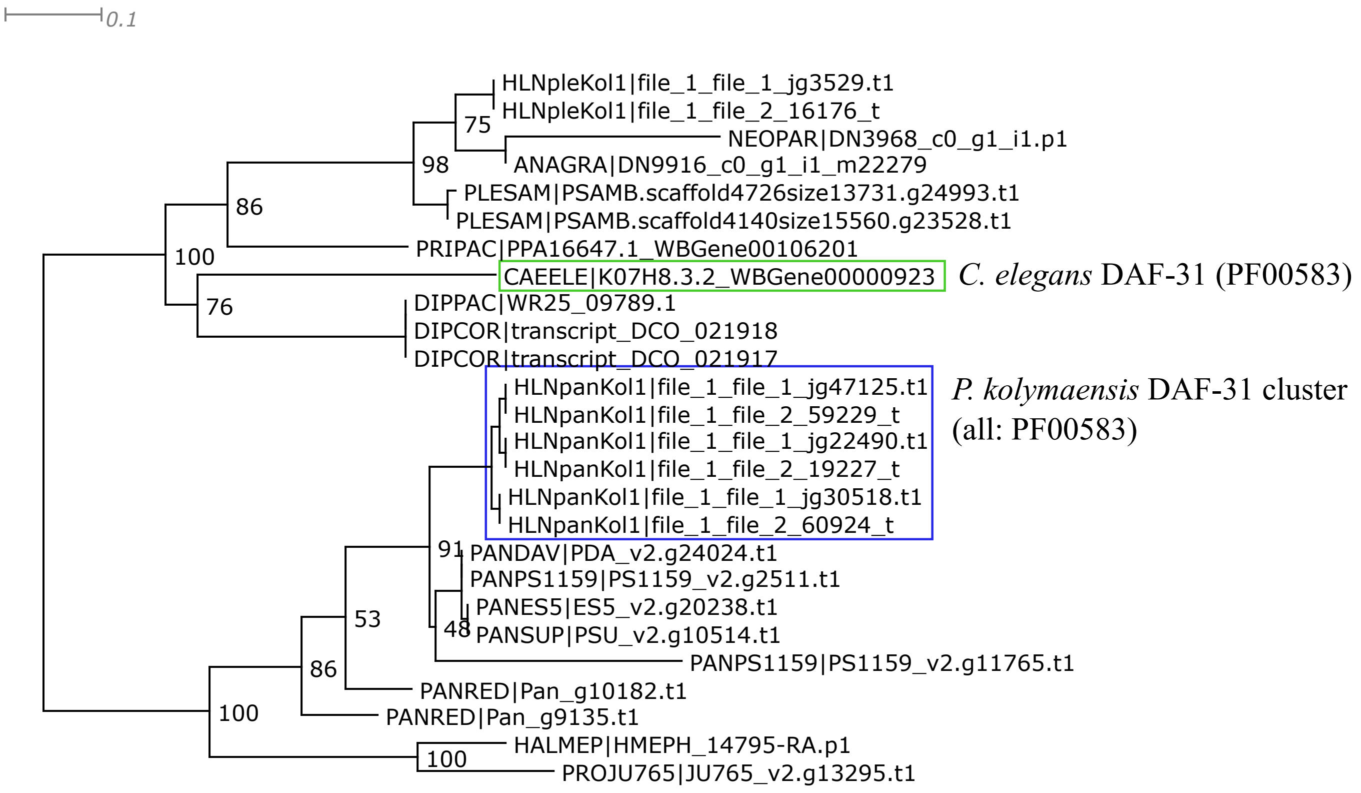

Trimal automated1 function; Short and spurious sequences removed manuallly.
IQtree2 ML phylogeny best-fit model according to BIC: LG+G4.

DAF-36

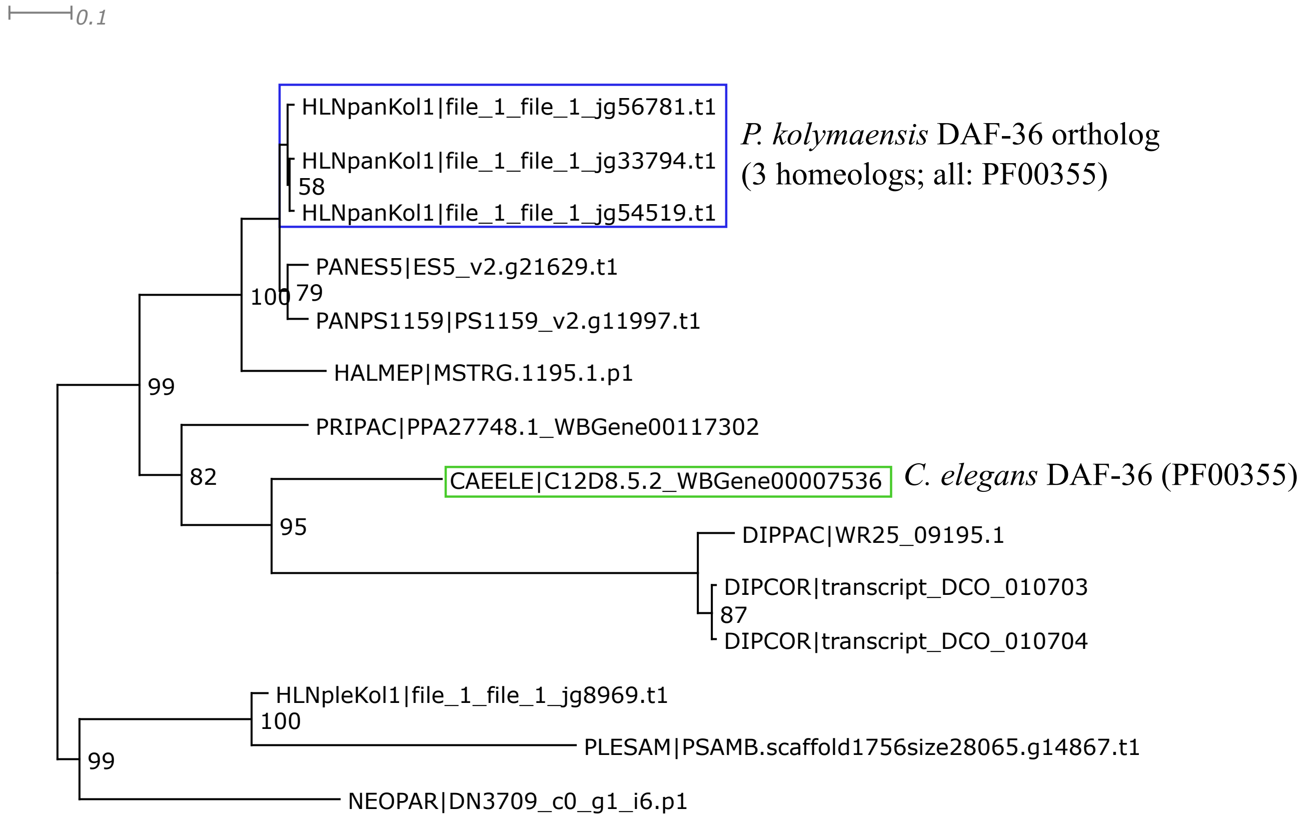

Trimal automated1 function; Short and spurious sequences removed manuallly.
IQtree2 ML phylogeny best-fit model according to BIC: LG+G4.

DAF-37

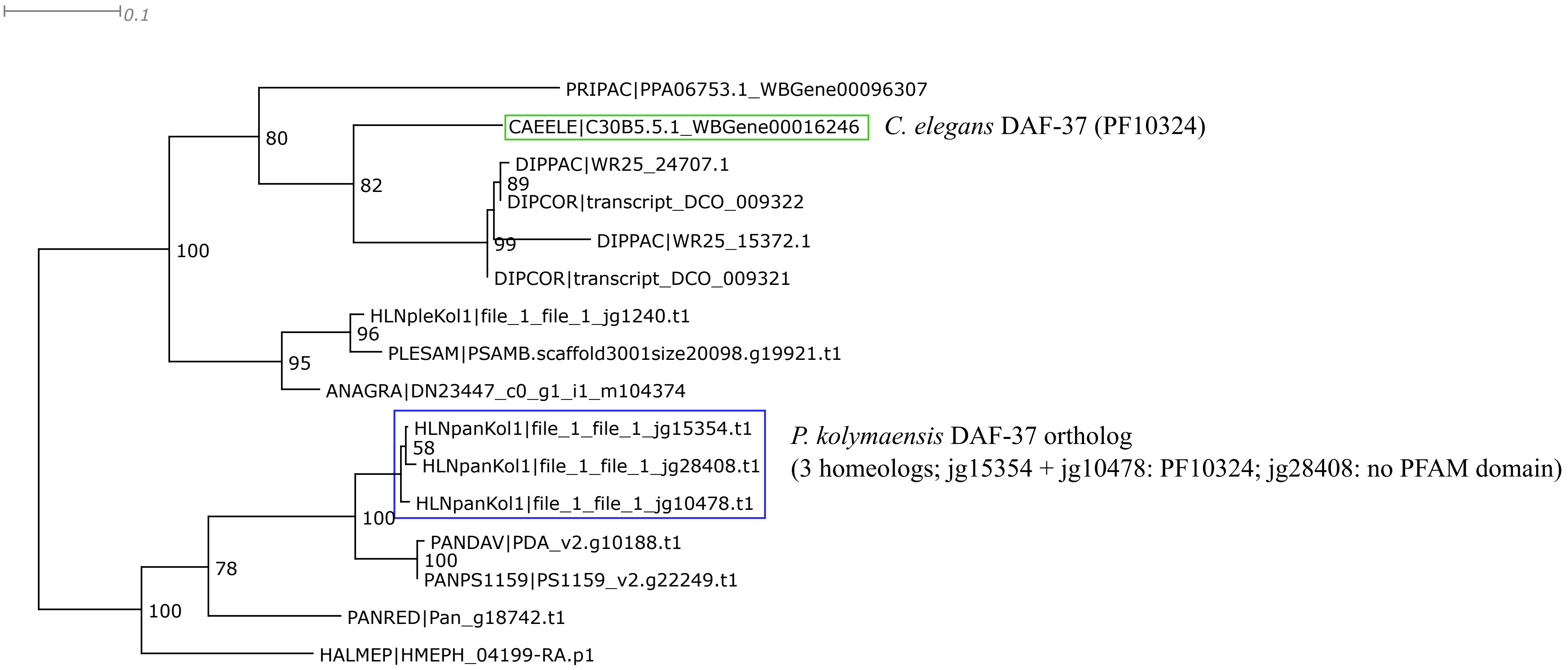

Trimal automated1 function; Short and spurious sequences removed manuallly.
IQtree2 ML phylogeny best-fit model according to BIC: LG+G4.

DAF-38

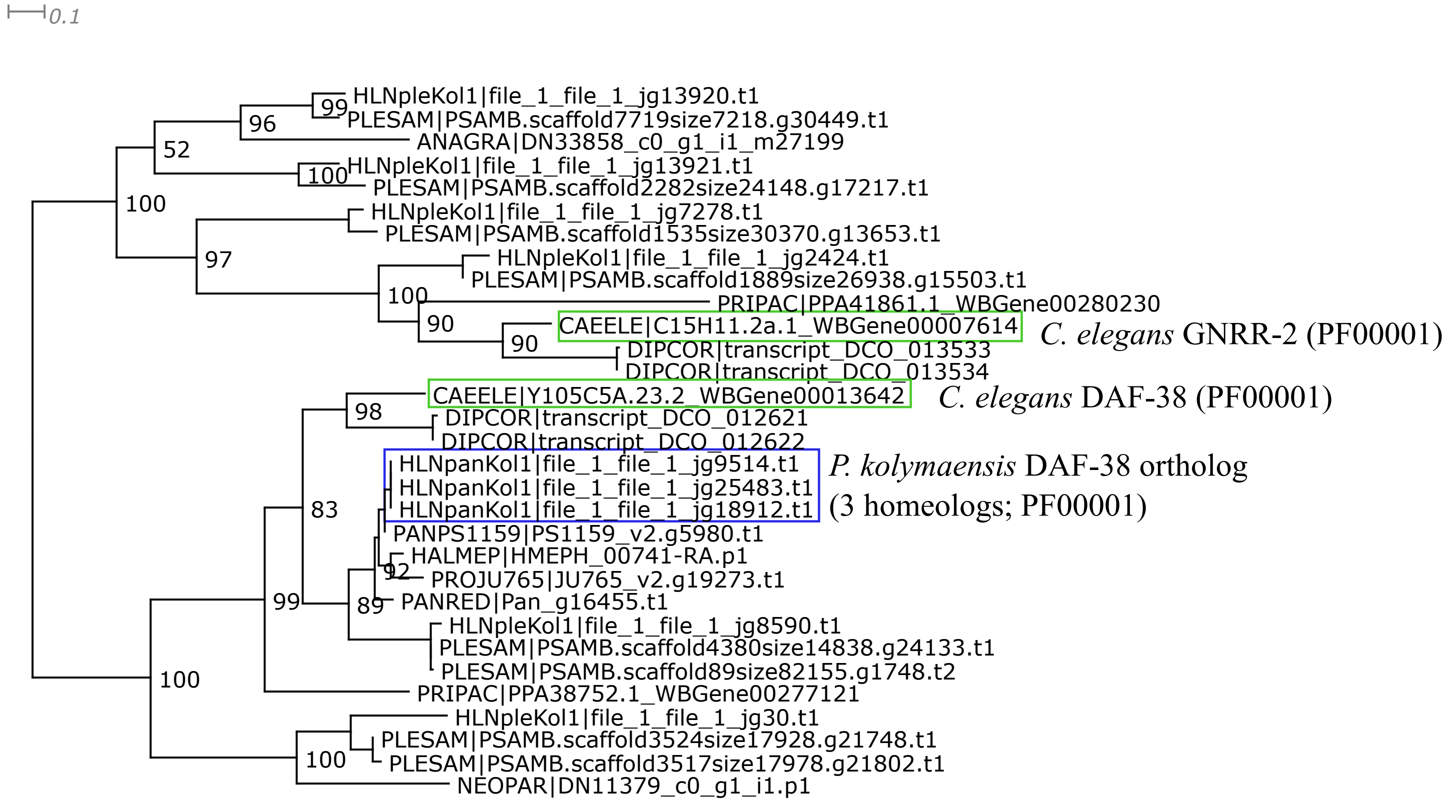

Trimal automated1 function; Short and spurious sequences removed manuallly.

IQtree2 ML phylogeny best-fit model according to BIC: mtInv+F+I+G4.

DAF-41

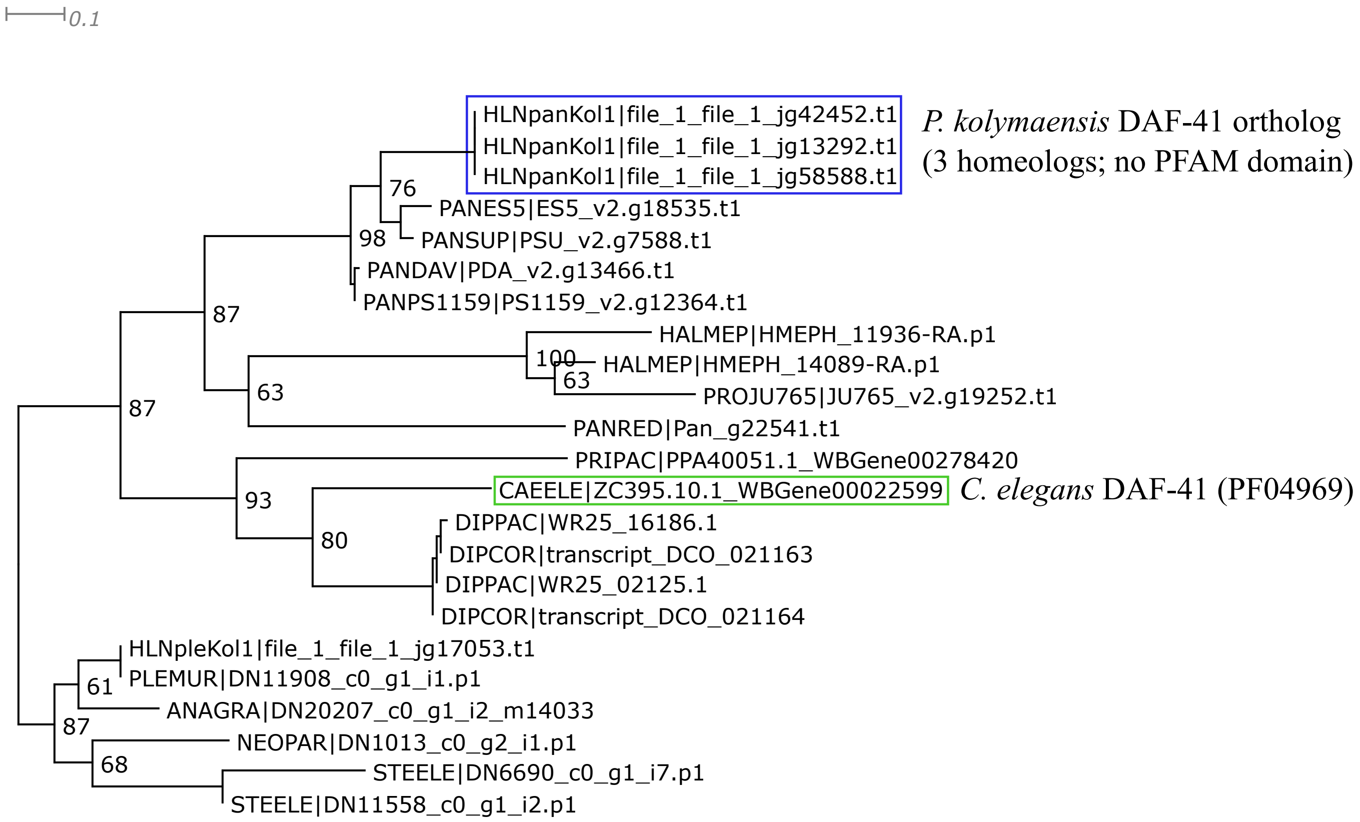

Trimal automated1 function; Trimal functions -resoverlap 0.8 -seqoverlap 80; Short and spurious sequences removed manuallly.
IQtree2 ML phylogeny best-fit model according to BIC: LG+G4.
Of all nematodes in this the phylogeny, the CS domain (PF04969) was only detected in *C. elegans* and some of the plectids.
