# Supplementary material for: A novel nematode species from the Siberian permafrost shares adaptive mechanisms for cryptobiotic survival with C. elegans dauer larva
Source: PLoS Genet. 2023 Jul 27;19(7):e1010798. doi: 10.1371/journal.pgen.1010798 (PMC10374039; doi:10.1371/journal.pgen.1010798)
Supplement: S3 Fig — A) The ploidy level was analysed with Smudgeplot v0.2.173. KMC version 3.1.074 was used to count the 21-mers in the PacBio CCS reads. Then we ran smudgeplot.py to determine the lower and upper coverage cut-offs. These were determined to be 14 and 380. 21-mers with a coverage between 14 and 380 were filtered with kmc_tools. Then, we computed the k-mer pairs from the filtered 21-mers by running smudgeplot.py hetkmers. Finally, the produced a smudgeplot shows an estimated ploidy of 3. B) Species tree inferred for 12,295 gene trees using coalescence approach. The species tree implemented using the coalescence approach with orthogroup gene tree is displayed. Novel species in this study are displayed in red. All nodes have a posterior probability of 1. C) Species tree inferred for 102 taxa. The maximum likelihood tree inferred using a concatenated supermatrix of 60 genes is displayed. Bootstrap values are only displayed for nodes with less than 100% support. The platyhelminth Macrostum lignano serves as an outgroup for rooting. The ancestral Panagrolaimus, sister to all others within the Panagrolaimus genus, is highlighted in red. (PDF) [file pgen.1010798.s003.pdf]

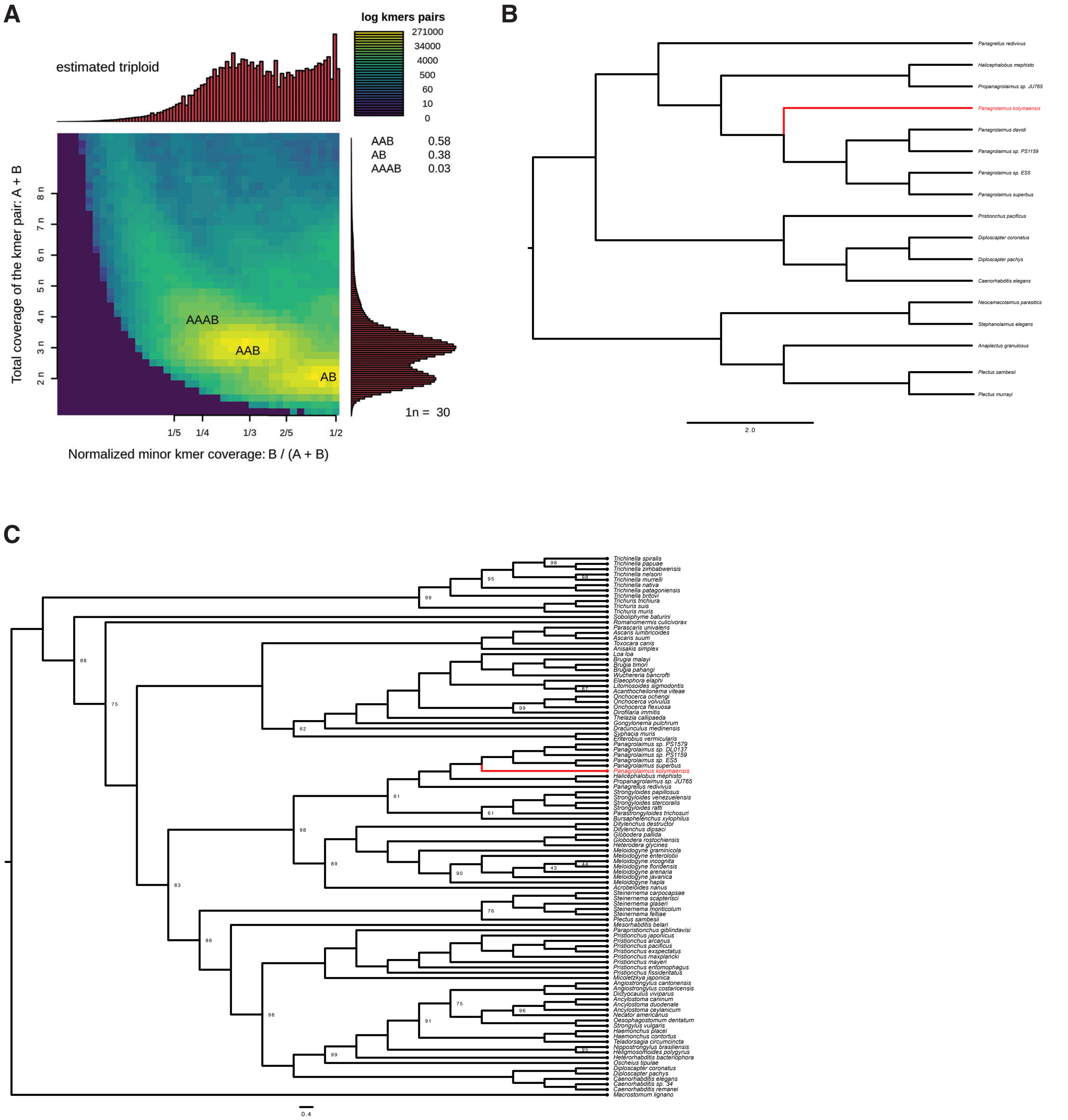
A
B
C
log kmers pairs
271000
34000
4000
500
60
10
0
estimated triploid
AAB 0.58
AB 0.38
AAAB 0.03
AAAB
AAB
AB
1n = 30
Total coverage of the kmer pair: A + B
Normalized minor kmer coverage: B / (A + B)
1/5
1/4
1/3
2/5
1/2
Panagrellus redivivus
Halicephalobus mephisto
Propanagrolaimus sp. JU765
Panagrolaimus kolymaensis
Panagrolaimus davidi
Panagrolaimus sp. PS1159
Panagrolaimus sp. ES5
Panagrolaimus superbus
Pristionchus pacificus
Diploscapter coronatus
Diploscapter pachys
Caenorhabditis elegans
Neocamacolaimus parasitics
Stephanolaimus elegans
Anaplectus granulosus
Plectus sambesii
Plectus murrayi
2.0
Trichinella spiralis
Trichinella papuae
Trichinella zimbabwensis
Trichinella nelsoni
Trichinella murrelli
Trichinella nativa
Trichinella patagoniensis
Trichinella britovi
Trichuris trichiura
Trichuris suis
Trichuris muris
Soboliphyme baturini
Romanomermis culicivorax
Parascaris univalens
Ascaris lumbricoides
Ascaris suum
Toxocara canis
Anisakis simplex
Loa loa
Brugia malayi
Brugia timori
Brugia pahangi
Wuchereria bancrofti
Elaeophora elaphi
Litomosoides sigmodontis
Acanthocheilonema viteae
Onchocerca ochengi
Onchocerca volvulus
Onchocerca flexuosa
Dirofilaria immitis
Thelazia callipaeda
Gongylonema pulchrum
Dracunculus medinensis
Syphacia muris
Enterobius vermicularis
Panagrolaimus sp. PS1579
Panagrolaimus sp. DL0137
Panagrolaimus sp. PS1159
Panagrolaimus sp. ES5
Panagrolaimus superbus
Panagrolaimus kolymaensis
Halicephalobus mephisto
Propanagrolaimus sp. JU765
Panagrellus redivivus
Strongyloides papillosus
Strongyloides venezuelensis
Strongyloides stercoralis
Strongyloides ratti
Parastrongyloides trichosuri
Bursaphelenchus xylophilus
Ditylenchus destructor
Ditylenchus dipsaci
Globodera pallida
Globodera rostochiensis
Heterodera glycines
Meloidogyne graminicola
Meloidogyne enterolobii
Meloidogyne incognita
Meloidogyne floridensis
Meloidogyne arenaria
Meloidogyne javanica
Meloidogyne hapla
Acrobeloides nanus
Steinernema carpocapsae
Steinernema scapterisci
Steinernema glaseri
Steinernema monticolum
Steinernema feltiae
Plectus sambesii
Mesorhabditis belari
Parapristionchus giblindavisi
Pristionchus japonicus
Pristionchus arcanus
Pristionchus pacificus
Pristionchus exspectatus
Pristionchus maxplancki
Pristionchus mayeri
Pristionchus entomophagus
Pristionchus fissidentatus
Micoletzkya japonica
Angiostrongylus cantonensis
Angiostrongylus costaricensis
Dictyocaulus viviparus
Ancylostoma caninum
Ancylostoma duodenale
Ancylostoma ceylanicum
Necator americanus
Oesophagostomum dentatum
Strongylus vulgaris
Haemonchus placei
Haemonchus contortus
Teladorsagia circumcincta
Nippostrongylus brasiliensis
Heligmosomoides polygyrus
Heterorhabditis bacteriophora
Oscheius tipulae
Diploscapter coronatus
Diploscapter pachys
Caenorhabditis elegans
Caenorhabditis sp. 34
Caenorhabditis remanei
Macrostomum lignano
0.4
