# Supplementary material for: A novel nematode species from the Siberian permafrost shares adaptive mechanisms for cryptobiotic survival with C. elegans dauer larva
Source: PLoS Genet. 2023 Jul 27;19(7):e1010798. doi: 10.1371/journal.pgen.1010798 (PMC10374039; doi:10.1371/journal.pgen.1010798)
Supplement: Attachment — Submitted filename: Response to the reviewers .pdf [file pgen.1010798.s016.pdf]

Dear Dr. Copenhaver,

Thank you very much for giving us the opportunity to reply to the reviewer's comments and to improve our manuscript. We submit the revised manuscript and provide a detailed point- by-point reply to the comments. As suggested, we now carefully checked and made sure Latin binomials throughout the manuscript are same. As requested, we provide all the numerical data and summary statistics in the form of spreadsheets in the supporting information. Large-scale datasets are already made available via a public repository (https://zenodo.org/record/7255559). We hope that we addressed all the comments of the reviewer, and the manuscript is now acceptable for publishing at PLOS Genetics.

With best wishes,

Temo Kurzchalia
(For all authors)

Comments to the Authors:

Please note here if the review is uploaded as an attachment.

Reviewer #1: The authors now provide accession numbers to sequences, thank you. A glaring error is failure to include the new species name in the manuscript. It is incorrect to name the species "n. sp." (as redundantly in line 163: "Box 1 Description of Panagrolaimus n. sp sp. nov." (also line 165) or line 189: Panagrolaimus sp. nov. Please also note that the "n. sp." should not be italicized. The authors already provide a species name near the bottom of box 1, "P. kolymaensis". Please use this name throughout the paper.

It is not correct to refer to a trait as "monophyletic". That is a term applied to taxa. What the authors mean is "apomorphic" (and instead of "polyphyletic", a trait would be "homoplastic").

Have all data underlying the figures and results presented in the manuscript been provided?
Large-scale datasets should be made available via a public repository as described in the PLOS Genetics data availability policy, and numerical data that underlies graphs or summary statistics should be provided in spreadsheet form as supporting information.

Reviewer #1: Yes

Response to reviewers:

Please note here if the review is uploaded as an attachment.

Reviewer #1:

1. The authors now provide accession numbers to sequences, thank you. A glaring error is failure to include the new species name in the manuscript. It is incorrect to name the species "n. sp." (as redundantly in line 163: "Box 1 Description of Panagrolaimus n. sp sp. nov." (also line 165) or line 189: Panagrolaimus sp. nov. Please also note that the "n. sp." should not be italicized. The authors already provide a species name near the bottom of box 1, "P. kolymaensis". Please use this name throughout the paper.

   We thank the reviewer for bringing this to our attention. We now changed the name of the species to *P. kolymaensis* throughout the manuscript. As our manuscript was reviewed at PCI and it is PCI policy not to provide the species name if the manuscript is in the form of preprint.

2. It is not correct to refer to a trait as "monophyletic". That is a term applied to taxa. What the authors mean is "apomorphic" (and instead of "polyphyletic", a trait would be "homoplastic").

   We thank the reviewer for this comment, and it is certainly right. We now changed the sentence accordingly. “Our data also contradicts the assumption that all parthenogenetic strains in the genus *Panagrolaimus* have a monophyletic origin” (Lines 176-177).

3. Have all data underlying the figures and results presented in the manuscript been provided? Large-scale datasets should be made available via a public repository as described in the PLOS Genetics data availability policy, and numerical data that underlies graphs or summary statistics should be provided in spreadsheet form as supporting information.

We now provided the spreadsheets and summary statistics for all the plots in the manuscript in the supporting information.
